# Supplementary material for: Association of Liver Damage and Quasispecies Maturity in Chronic HCV Patients: The Fate of a Quasispecies
Source: Microorganisms. 2024 Oct 31;12(11):2213. doi: 10.3390/microorganisms12112213 (PMC11596025; doi:10.3390/microorganisms12112213)
Supplement: Supplementary file 1 [file microorganisms-12-02213-s001.zip › Main Supplementary Materials.pdf]

Liver damage vs HCV quasispecies maturity

Supplementary material: Methods & Results

Josep Gregori*, Marta Ibañez-Lligoña, Sergi Colomer-Castell, Carolina Campos, Damir García-Cehic, and Josep Quer†

2024-10-30

Contents

S1 Supplementary methods **2**
S1.1 Patient data 2
S1.2 Indicators of quasispecies maturity 2
S1.3 Effect size metrics 3

S2 Supplementary results **6**
S2.1 Boxplots of selected indicators, by amplicon and fibrosis stage 6
S2.2 Variability of indicators by fibrosis stage 9
S2.3 Coverage and the need for rarefaction 12
S2.4 Results on non-rarefied (raw) quasispecies 14
S2.5 Rarefied vs raw non-rarefied quasispecies results 19

References **26**

*josep.gregori@gmail.com
†josep.quer@vhir.org

S1 Supplementary methods

S1.1 Patient data

Table S1: Patients with fibrosis scores used in the association study. PrevTrt: Any previouly failed treatment; FailedTrt: Last failed treatment; RBV: Last treatment with/without ribavirin.

ID	Gtp	Stage	PrevTrt	FailedTrt	RBV
Pt219	1a	F1	n.a.	PTV+OMV	YES
Pt221	1a	F1	n.a.	n.a.	n.a.
Pt099	1b	F2	NO	SMV+SOF	NO
Pt161	1b	F2	NO	PTV+OMV+DSV	NO
Pt193	1a	F2	n.a.	LDV+SOF	NO
Pt014	1a	F3	NO	PTV+OMV+DSV	YES
Pt207	4d	F3	PTV+OMV	LDV+SOF	YES
Pt176	3a	F3	NO	DCV+SOF	NO
Pt204	1a	F3	n.a.	n.a.	n.a.
Pt222	3a	F3	n.a.	n.a.	n.a.
Pt035	1b	F4+	NO	LDV+SOF	YES
Pt206	1b	F4+	Unk	LDV+SOF	NO
Pt090	1b	F4+	NO	SMV+SOF	NO
Pt091	1b	F4+	NO	SMV+SOF	NO
Pt079	3a	F4+	NO	SOF	YES
Pt037	3a	F4+	NO	DCV+SOF	YES
Pt034	1a	F4+	NO	SMV+SOF	YES
Pt038	1a	F4+	PegIFN+TPV+RBV	DCV+SOF	NO
Pt200	3a	F4+	NO	DCV+SOF	YES

Treatments: DCV daclatasvir, DSV dasabuvir, LDV ledipasvir, OMV ombitasvir, PegIFN peginterferon-alfa, PTV paritaprevir, RBV ribavirin, SMV simeprevir, SOF sofosbuvir, TVR telaprevir.

S1.2 Indicators of quasispecies maturity

The next table shows the expected level of each quasispecies maturity indicator in the two limiting cases, flat vs regular quasispecies (Gregori, Colomer-Castell, et al. 2024):

Table S2: Maturity indicators and expected level per type.

Feature	Description	Regular Qs	Flat Qs
Master	Dominant haplotype frequency	High	Low
Rare	Fraction of reads for hpl $\leq 1\%$	Low	High
Top25	Fraction of reads for top 25 hpl.	High	Low
Top25R	Ratio Top25 to master reads	Low	High
Singl	Fraction of singletons	Low	High
RLE_1	Relative logarithmic evennes at $q = 1$	Low	High
RLE_2	Relative logarithmic evennes at $q = 2$	Low	High
RLE_∞	Relative logarithmic evennes at $q = \infty$	Low	High
Rk	Evenness on top k haplotypes (k=10, 25)	Low	High

With

$$RLEq = RLE(p,q) = \log_{10}(D(p,q))/\log_{10}(D(p,0))$$

and $D(q,p)$ the Hill number of order q.

$$D(q,p) = \left(\sum_{i=1}^{H} p_i^q\right)^{1/(1-q)}$$

H number of haplotypes, $p = (p_1, p_2, \ldots, p_H)$ the set of haplotype frequencies.

On the set of haplotype frequencies sorted in decreasing order, $p_1 \geq p_2 \geq \cdots \geq p_H$, the following indicators are computed:

$$\text{Top25}(p) = \left(\sum_{i=1}^{m} p_i \;\middle|\; m = \min(25, H)\right)$$

$$\text{Top25R}(p) = \frac{\text{Top25}(p)}{p_1}$$

and an indicator of evenness on the set of k top haplotypes:

$$R_k(p) = \left(\frac{l \cdot p_l}{\sum_{i=1}^{l} p_i} \;\middle|\; l = \min(k, H)\right)$$

S1.3 Effect size metrics

S1.3.1 AUC

AUC, besides being the area under the ROC curve (Altman and Bland 1994; Zweig and Campbell 1993), is defined as the proportion of favorable evidence, i.e. the proportion of pairs $f(X) > f(Y)$ in our dataset, when the hypothesis is $f(X) > f(Y)$, and where X are the samples of one class (A) and Y samples of the second class (B) (Hanley and McNeil 1982).. Here $f(X)$ represents the value for the index f applied to the sample X.

$$AUC(f) = \frac{\sum_{X \in A} \sum_{Y \in B} I(f(X) > f(Y))}{n_A \cdot n_B}$$

with $I(\cdot)$ the characteristic function, resulting in 1 if the condition between the parenthesis is fulfilled, or 0 otherwise.

This expression is closely related to the Wilcoxon-Mann-Whitney test, from which statistic, U, may be obtained directly, as $AUC = U/(n_A \cdot n_B)$.

An AUC value of 0.5 represents a purely random classification, increasing values above 0.5 represent higher discrimination capacity, with perfect discrimination at AUC=1.

If $AUC(f)$ results in values below 0.5, by inverting the model's predictions (e.g., predicting class A when the model predicts class B and vice versa), the AUC could theoretically be improved to $1 - AUC$. In practice this represents inverting the relationship, form $f(A) \geq f(B)$ to $f(B) \geq f(A)$. We followed this rule when computing AUC values, so that in practice it represents how above random the indicator is able to discriminate between the two classes.

AUC is also known as the common language effect size, because its probabilistic terms allow a translation to common language more easily that with other statistics used in scoring the effect size, and because it provides a level of discrimination between the two classes.

Table S3: AUC effect magnitude.

AUC	Discrimination
1	Perfect
[0.9 - 1.0[	Very high
[0.8 - 0.9[	High
[0.7 - 0.8[	Moderate
[0-6 - 0-7[	Slight
[0.5 - 0.6[	Null

S1.3.2 Rank biserial correlation

The rank biserial correlation can be expressed as the difference between the proportion of favorable evidence f minus the proportion of unfavorable evidence u (Cureton 1956).

$$R_{RB} = f - u = 2f - 1$$

where $f = AUC$ is related to the Mann-Whitney statistics, as seen before, as:

$$f = \frac{U}{n_1 \, n_2}$$

Consequently,

$$R_{RB} = AUC - (1 - AUC) = 2\, AUC - 1$$

R_{RB} varies between 0 and 1, with 0 representing chance classification and 1 perfect discrimination.

Table S4: R_{BR} effect magnitude.

R_BR	Discrimination
1	Perfect
[0.8 - 1.0[	Very high
[0.6 - 0.8[	High
[0.4 - 0.6[	Moderate
[0.2 - 0.4[	Slight
[0.0 - 0.2[	Null

S1.3.3 Robust Cohen's delta

Next we compute another nonparametric effect size metric, $\gamma_{0.5}$, as a robust version of the traditional Cohen's δ, $\delta = (\bar{y} - \bar{x})/s$. The nonparametric version replaces the mean by the median, and the pooled standard deviation by the pooled median absolute deviation (Lötsch and Ultsch 2020; Akinshin 2020).

$$\gamma_{0.5} = \frac{Q_{0.5}(y) - Q_{0.5}(x)}{PMAD(x, y)}$$

$$PMAD(x,y) = \sqrt{\frac{(n_x - 1)\ MAD(x)^2 + (n_y - 1)\ MAD(y)^2}{n_x + n_y - 2}}$$

$$MAD(x) = 1.4826 \cdot Q_{0.5}(|x - Q_{0.5}(x)|)$$

Where $Q_{0.5}(x)$ is the median of x. The factor 1.4826 is taken for asymptotically normal consistency, from the equallity $\sigma = 1.4826 \cdot MAD$ for normally distributed data. Taking into account this factor, for normally distributed data we have the equality $\gamma_{0.5} = \delta$.

A rule-of-thumb for interpreting $\gamma_{0.5}$ values in effect size terms is as follows (Sawilowsky 2009):

Table S5: $\gamma_{0.5}$ effect magnitude.

Effect size	$\gamma_{0.5}$
Very small	0.01
Small	0.20
Medium	0.50
Large	0.80
Very large	1.20
Huge	2.00

S2 Supplementary results

S2.1 Boxplots of selected indicators, by amplicon and fibrosis stage

In an exploratory data analysis, boxplots of all quasispecies structure indicators per liver fibrosis level, represent a first visual assessment about the separability between liver damage stages provided by each indicator.

Table S6: Amplicons per fibrosis stage and genomic region.

Stage	NS3	NS5A	NS5B1	NS5B2	Globally
F1	2	2	1	1	6
F2	3	3	3	2	11
F3	4	5	5	2	16
F4+	8	9	9	8	34

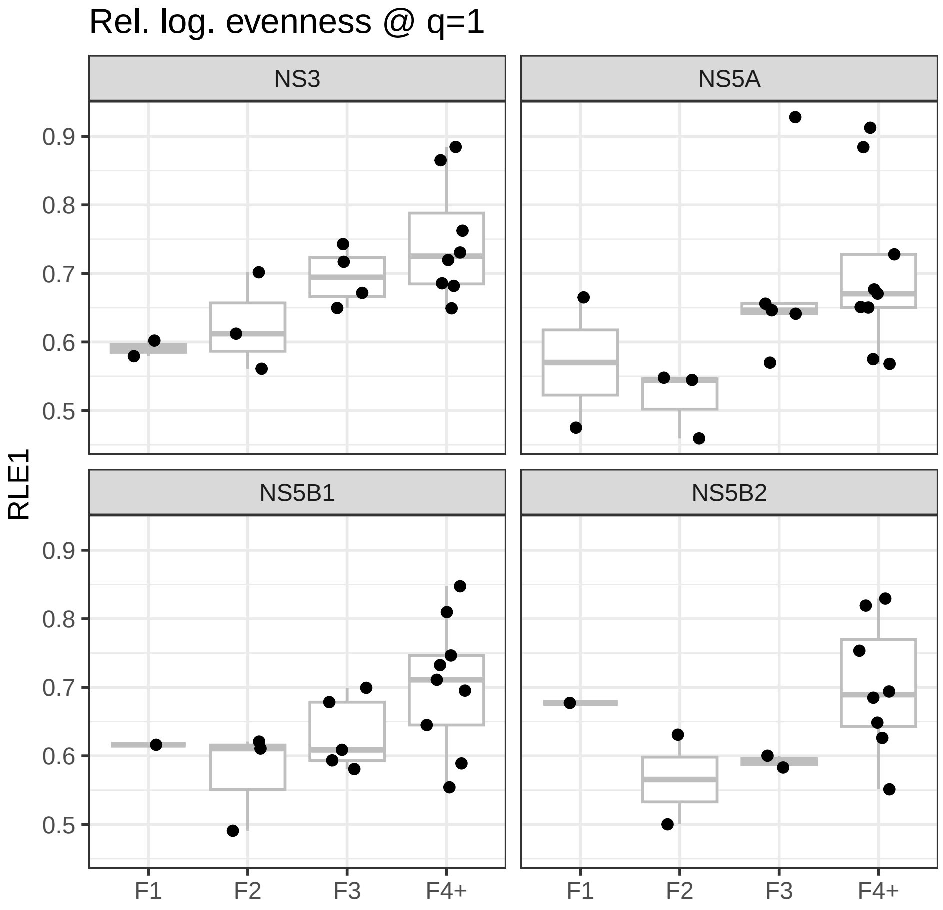

Figure S1: Boxplots showing indicator distribution per amplicon and fibrosis stage.

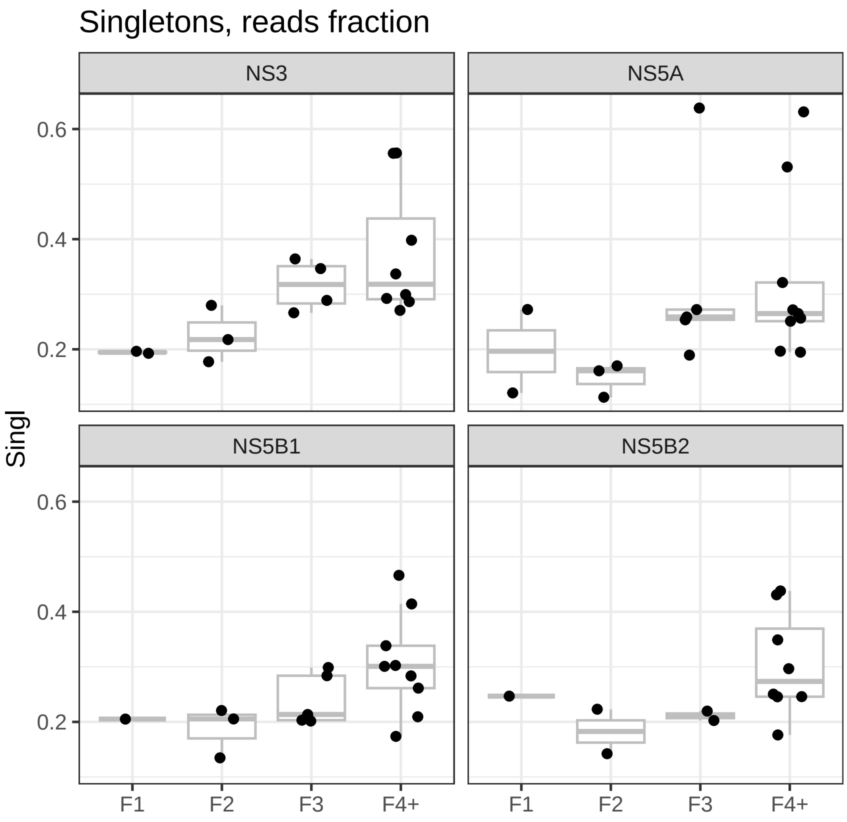
Singletons, reads fraction
NS3
NS5A
NS5B1
NS5B2
Singl
0.2
0.4
0.6
F1
F2
F3
F4+

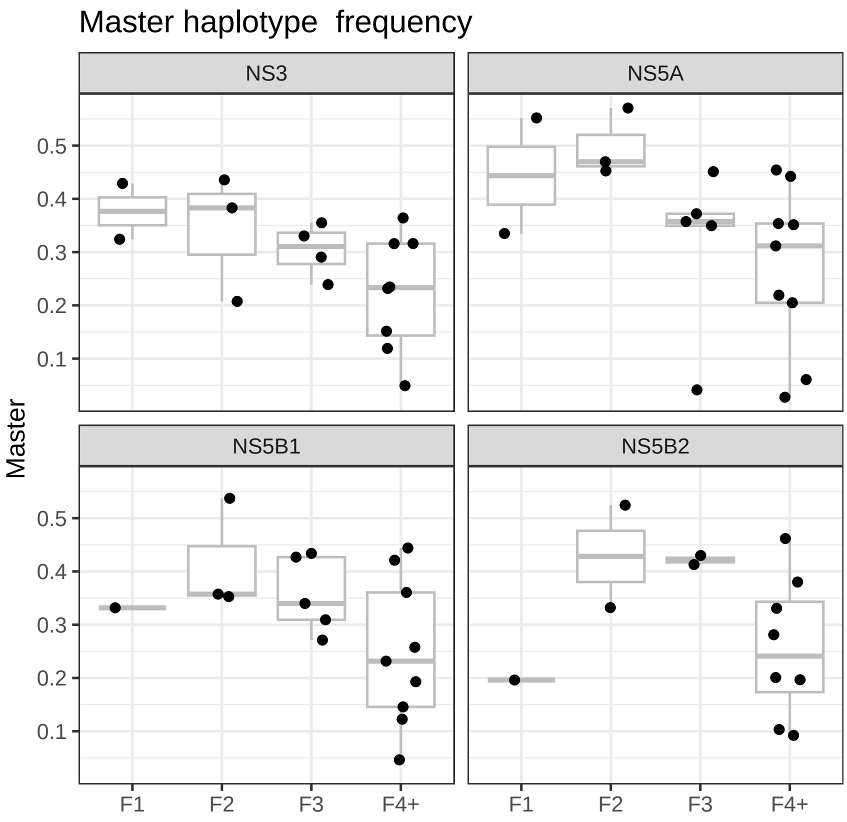
Master haplotype frequency
NS3
NS5A
NS5B1
NS5B2
Master
0.1
0.2
0.3
0.4
0.5
F1
F2
F3
F4+

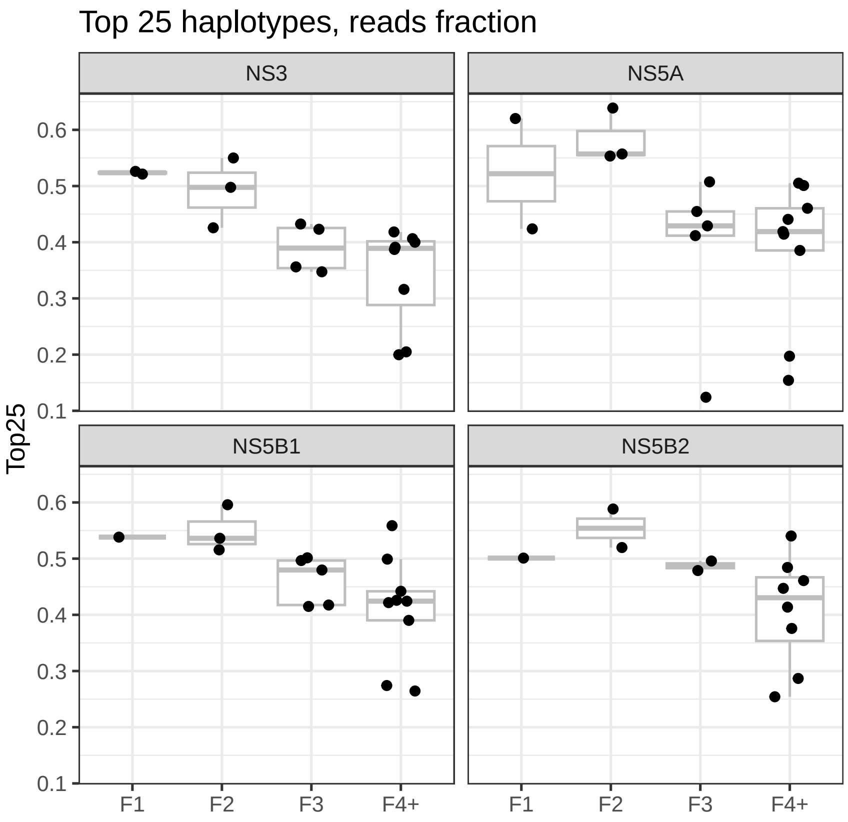
Top 25 haplotypes, reads fraction
NS3
NS5A
NS5B1
NS5B2
Top25
0.1
0.2
0.3
0.4
0.5
0.6
F1
F2
F3
F4+

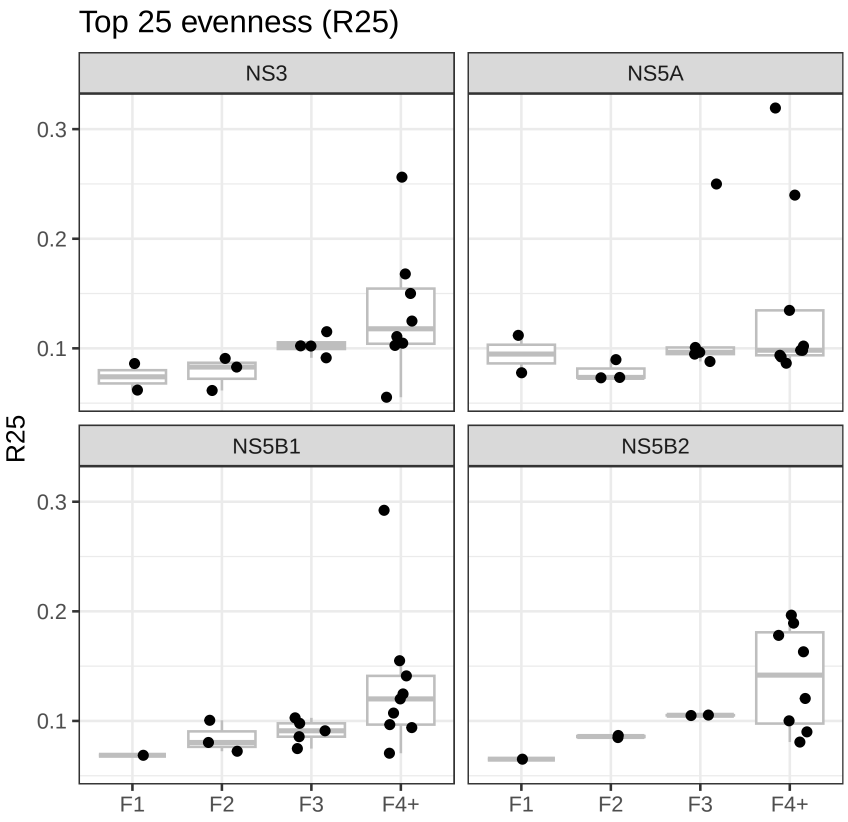
Top 25 evenness (R25)
NS3
NS5A
NS5B1
NS5B2
R25
0.1
0.2
0.3
F1
F2
F3
F4+

S2.2 Variability of indicators by fibrosis stage

S2.2.1 Normalized Median Absolute Deviation

Let's study the variability with which each indicator is observed, and if its is growing or schrinking with increasing fibrosis. The measure of variability selected with this purpose is the Normalized Median Absolute Deviation which is a robust coefficient of variation (RCV):

$$RCV(X) = \frac{MAD(X)}{Q_{0.5}(X)}; \quad \text{with} \quad MAD(X) = Q_{0.5}(|X - Q_{0.5}(X)|)$$

where $Q_{0.5}$ is the median value, and MAD is the Median Absolute Deviation (MAD). The variability is measured as the MAD, and is divided by the median, so that it becomes comparable between variables expressed in different ranges. The variability is expressed in units of median. Both MAD and $Q_{0.5}$ are robust to outliers.

RCV values:

Table S7: Normalized median absolute deviation of each quasispecies maturity indicator, per fibrois stage.

Stage	Master	Top25	Rare	Singl	RLE1	RLE2	RLEinf	R10	R25	Top25R
F1	15.72	3.561	1.851	13.47	7.044	10.78	12.76	34.36	13.200	14.120
F2	19.03	6.241	9.351	22.68	10.670	20.81	20.74	25.69	9.509	11.310
F3	17.35	11.270	7.304	17.50	7.641	14.37	14.36	11.64	5.892	6.464
F4+	48.17	10.210	10.250	15.73	7.307	24.30	27.82	52.27	21.070	29.690

S2.2.2 Wilcoxon test over all RCV

Table S8: Wilcoxon test results comparing the normalized median absolute deviation of all indicators between fibrosis levels.

Stg1	Stg2	pval	AUC
F1	F2	0.4812509	0.60
F2	F3	0.1903159	0.32
F3	F4+	0.0288056	0.79

The variability on F4+ is significantly higher than in F3 (p-values=0.029, AUC=0.79). Master, R10 and Top25R show big differences in variability in F4+ compared to F3.

S2.2.3 F3 to F2 RCV permutation test over each indicator

Permutation test

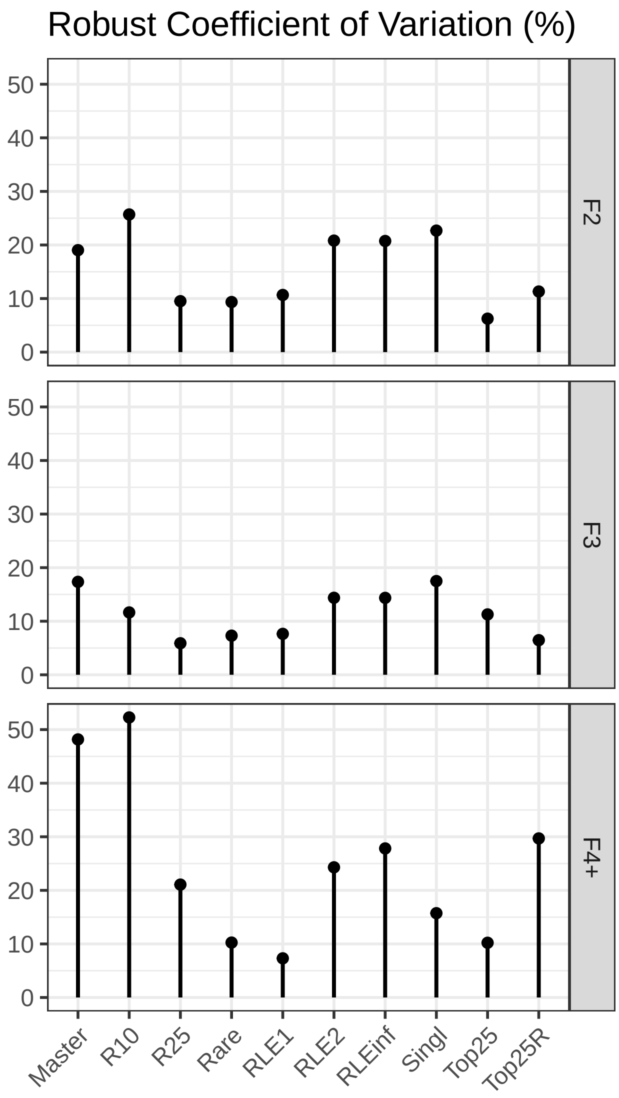

Figure S2: Normalized median absolute deviation of each quasispecies maturity indicator, per fibrois stage.

Table S9: Permutaion tests comparing the variability of each indicator between fibrosis stages F3 and F2. Sorted in decreasing order of RCV ratio.

	Diff	p_val_diff	Ratio	p_val_ratio
Top25	5.033442	0.2372	1.8064689	0.0912
Master	-1.681132	0.8332	0.9116569	0.6018
Rare	-2.046437	0.5404	0.7811449	0.7562
Singl	-5.183170	0.5178	0.7714671	0.7724
RLE1	-3.025460	0.3906	0.7163626	0.7764
RLEinf	-6.383338	0.2748	0.6922311	0.8332
RLE2	-6.435277	0.2870	0.6907596	0.8266
R25	-3.617149	0.4444	0.6196005	0.8626
Top25R	-4.846849	0.4716	0.5714761	0.7744
R10	-14.050654	0.0754	0.4531480	0.9674

S2.2.4 F4 to F3 RCV permutation test over each indicator

Permutation test

Table S10: Permutaion tests comparing the variability of each indicator between fibrosis stages F4+ and F3. Sorted in decreasing order of RCV ratio.

	Diff	p_val_diff	Ratio	p_val_ratio
Top25R	23.2289719	0.0176	4.5937474	0.0098
R10	40.6317113	0.0008	4.4897828	0.0002
R25	15.1808070	0.0520	3.5766574	0.0090
Master	30.8178811	0.0314	2.7764053	0.0290
RLEinf	13.4631848	0.0740	1.9377207	0.0560
RLE2	9.9225621	0.1058	1.6902811	0.0724
Rare	2.9443010	0.4252	1.4030963	0.2664
RLE1	-0.3338412	0.9374	0.9563103	0.5236
Top25	-1.0599382	0.9164	0.9059903	0.5282
Singl	-1.7629600	0.8842	0.8992423	0.5952

R10, R5, Top25R, and Master in F4+ vs F3, are the only statistically different robust coefficients of variation, with ratios:

- RCV(F4+,Top25) / RCV(F3,Top25) = 4.59.
- RCV(F4+,R10) / RCV(F3,R10) = 4.49.
- RCV(F4+,R25) / RCV(F3,R25) = 3.58.
- RCV(F4+,Master) / RCV(F3,Master) = 2.78

S2.3 Coverage and the need for rarefaction

S2.3.1 Coverage boxplots, non-rarefied quasispecies

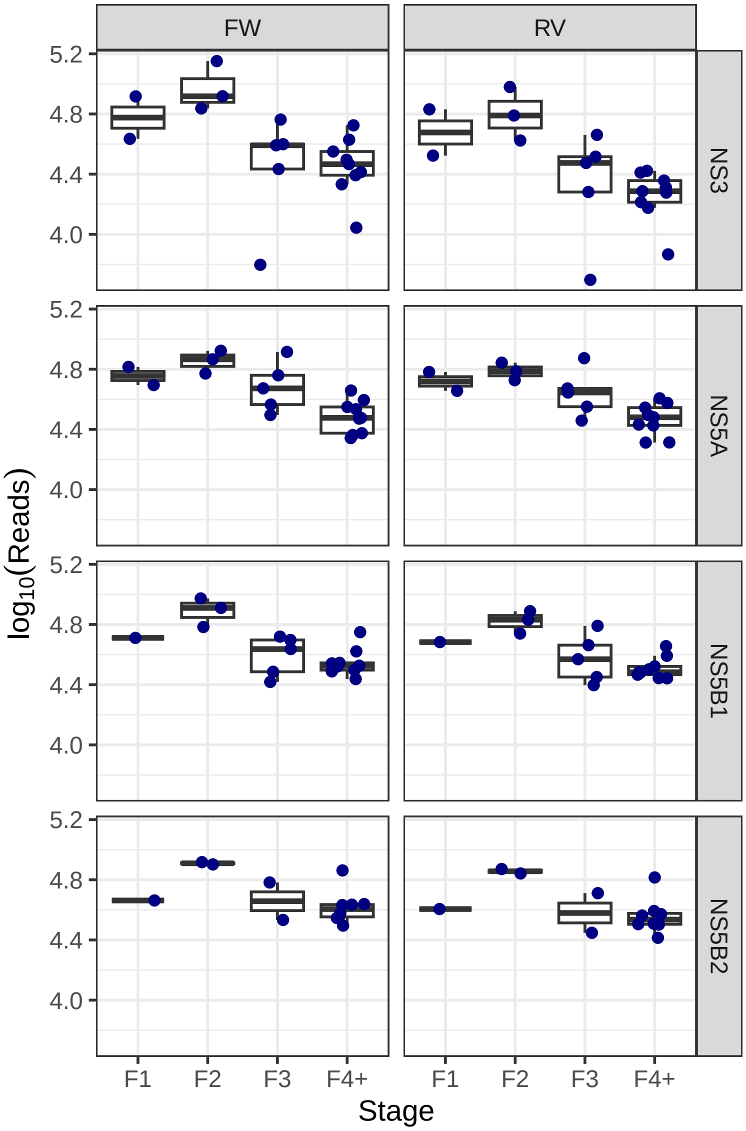

Figure S3: Coverage distribution as number of reads per amplicon/strand for each amplicon and fibrosis stage. Represented in log_{10} scale.

In all amplicons and strands we observe the same tendency to lower coverages as we go from F2 to F3, and from F3 to F4+. We showed in a previous work that the estimated value for evenness indices (RLE1, RLE2, RLEinf) and for singletons is slightly higher for lower coverages, and that other indices like Master, Top25 and Rare are relatively robust to coverage differences. The observed coverage differences will likely introduce some bias towards higher values in RLE1, RLE2, and RLEinf for growing fibrosis stages. This artificial bias is corrected by rarefaction

See below under *Results on non-rarefied quasispecies* and *Rarefied vs non-rarefied quasispecies results.*

Table S11: Median coverage as reads number per amplicon

Ampl	Stage	FW	RV	Total
NS3	F2	82732	61630	144362
NS3	F3	39071	29821	68892
NS3	F4+	29266	19348	48614
NS5A	F2	73427	61136	134563
NS5A	F3	47082	44202	91284
NS5A	F4+	29975	30236	60211
NS5B1	F2	81326	67872	149198
NS5B1	F3	43321	37092	80413
NS5B1	F4+	33570	30390	63960
NS5B2	F2	81234	71935	153169
NS5B2	F3	47330	39717	87047
NS5B2	F4+	40370	34358	74728

Table S12: Global distribution of coverage per fibrosis stage, non rarefied quasispecies.

Stage	Min	Median	Max
F1	33359	48871.0	82552
F2	42036	71584.0	141765
F3	4982	38081.5	82267
F4+	7355	31377.5	72783

S2.3.2 Coverage for rarefied quasispecies

Rarefaction process:

- Amplicons with a coverage below 18,000 are excluded from the computations.
- For amplicons with coverages above 18,000 and below 23,000, diversity values are computed with no rarefaction
- For amplicons with coverages above 23,000, diversity values are computer after quasispecies rarefaction to 20,000 reads.

Distribution of coverage for rarefied quasispecies

Stage	Min	Median	Max
F1	20000	20000	20000
F2	20000	20000	20000
F3	19112	20000	20000
F4+	18934	20000	22762

Table S14: Wilcoxon test results on raw quasispecies values. Only tests with p-values below 0.05 and AUC values above 0.7 are listed. alt.twosd: alternative H two sided; alt.less: alternative H Stg2<Stg1; alt.great: alternative H Stg2>Stg1.

Stg1	Stg2	Indicator	AUC	alt.twosd	alt.less	alt.great	Star
F2	F3	Master	0.7540	0.0252	0.0126	0.9890	*
F2	F3	Rare	0.8984	0.0002	0.9999	0.0001	***
F2	F3	Top25	0.9412	0.0000	0.0000	1.0000	***
F2	F3	Singl	0.9572	0.0000	1.0000	0.0000	***
F2	F3	RLE1	0.8449	0.0017	0.9993	0.0008	**
F2	F3	RLE2	0.7754	0.0146	0.9937	0.0073	*
F2	F3	RLEinf	0.7754	0.0146	0.9937	0.0073	*
F2	F3	R25	0.8556	0.0011	0.9995	0.0006	**
F3	F4+	Master	0.7008	0.0192	0.0096	0.9909	**
F3	F4+	RLE1	0.6807	0.0359	0.9829	0.0180	*
F3	F4+	RLE2	0.7193	0.0102	0.9952	0.0051	*
F3	F4+	RLEinf	0.7160	0.0115	0.9946	0.0058	*
F3	F4+	Top25R	0.7311	0.0066	0.9969	0.0033	**
F3	F4+	R10	0.7613	0.0020	0.9991	0.0010	**
F3	F4+	R25	0.7059	0.0162	0.9923	0.0081	*

S2.4 Results on non-rarefied (raw) quasispecies

We refer to the diversities computed on haplotype quasispecies frequencies as observed, without normalization, as *raw diversity values*. This means the diversity values are calculated directly from the observed haplotype frequencies without the rarefaction process to a common reference size. Normalization through rarefaction is necessary when comparing samples with unbalanced sequencing depths to avoid differential biases caused by dissimilar coverages (Gregori, Ibañez-Lligoña, et al. 2024). We refer to the diversities computed on rarefied quasispecies to a common reference size as *rarefied values*.

S2.4.1 Results of tests on raw quasispecies values

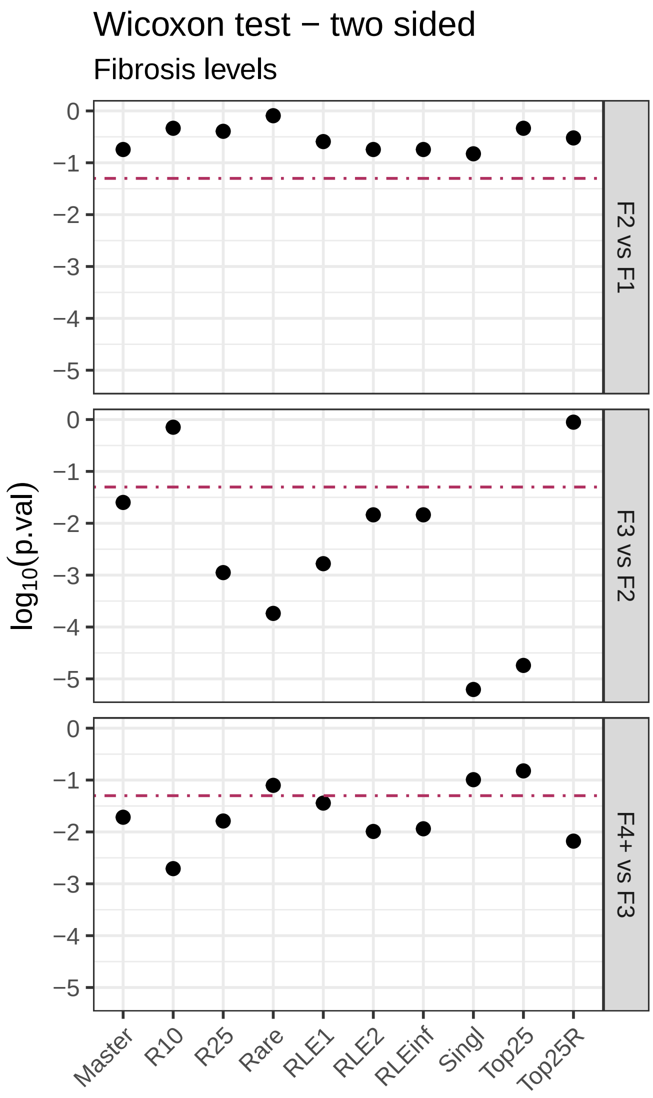

Figure S4: Wilcoxon test on raw quasispecies values, p-values plotted in log_{10} scale.

S2.4.2 Effect size, computed on raw values

S2.4.2.1 Area under the ROC curve

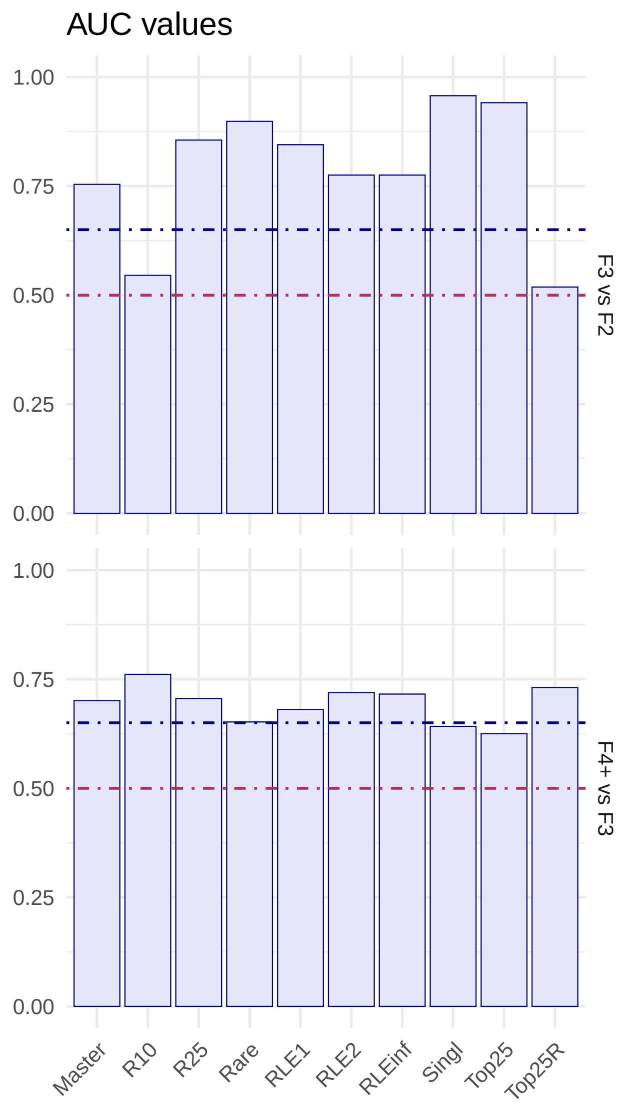

Figure S5: AUC values resulting from the Wilcoxon test on raw quasispecies values

S2.4.2.2 Robust Cohen's delta, $\gamma_{0.5}$

Indicator	F3 vs F2	F4+ vs F3
Master	-0.8281115	-0.7821054
Rare	1.5692197	0.3168496
Top25	-1.8191523	-0.2493701
Singl	1.3945772	0.4462934
RLE1	1.2787484	0.6834546
RLE2	0.7664937	0.9226603
RLEinf	0.8212242	0.7442197
Top25R	-0.2745017	0.7427543
R10	-0.2333480	0.6609650
R25	1.9716895	0.6376181

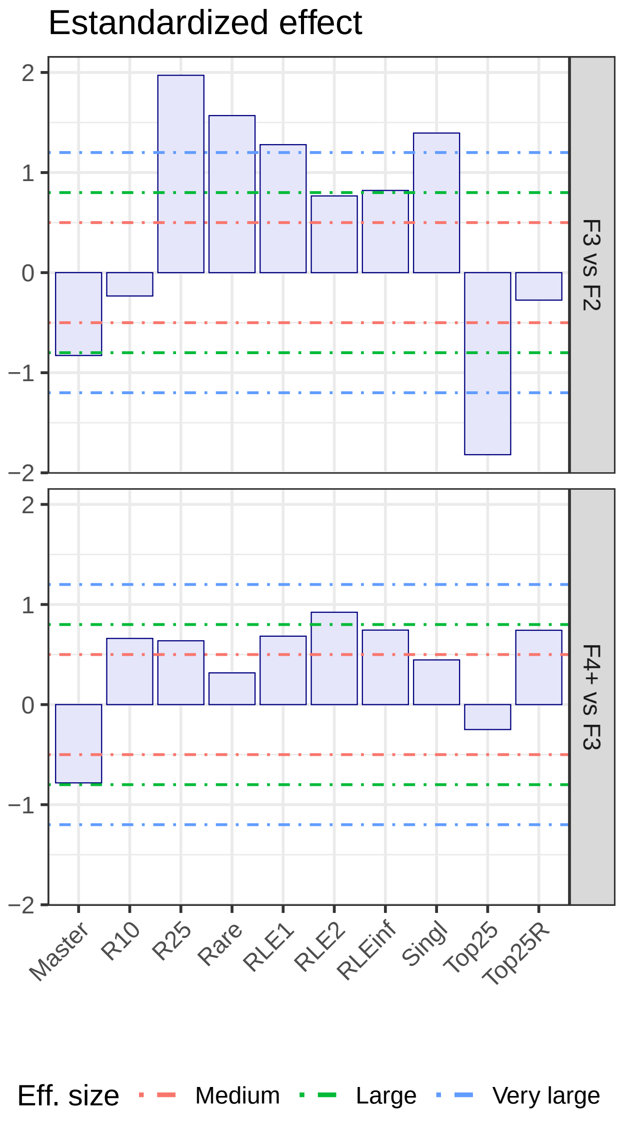

Figure S6: Standardized effect size gamma, with magnitude borders.

S2.4.3 Collected metrics of all comparisons on raw quasispecies

Quasispecies structure indicators sorted by decreasing AUC value, and collecting *p-value*, *AUC*, R_{BR} and $\gamma_{0.5}$.

Table S16: Collected metrics of all tests on raw values. Wilcoxon test p-values, and effect sizes as AUC, RBR and Gamma. Sorted in decreasing order of AUC value. Results of tests F2 vs F1 are not shown.

lbl	Indicator	p.val	AUC	RRB	Gamma	Signif
F3 vs F2	Singl	6.20e-06	0.957	0.9140	1.390	***
F3 vs F2	Top25	1.82e-05	0.941	0.8820	-1.820	***
F3 vs F2	Rare	1.83e-04	0.898	0.7970	1.570	***
F3 vs F2	R25	1.12e-03	0.856	0.7110	1.970	**
F3 vs F2	RLE1	1.66e-03	0.845	0.6900	1.280	**
F3 vs F2	RLE2	1.46e-02	0.775	0.5510	0.766	*
F3 vs F2	RLEinf	1.46e-02	0.775	0.5510	0.821	*
F4+ vs F3	R10	1.96e-03	0.761	0.5230	0.661	**
F3 vs F2	Master	2.52e-02	0.754	0.5080	-0.828	*
F4+ vs F3	Top25R	6.64e-03	0.731	0.4620	0.743	**
F4+ vs F3	RLE2	1.02e-02	0.719	0.4390	0.923	*
F4+ vs F3	RLEinf	1.15e-02	0.716	0.4320	0.744	*
F4+ vs F3	R25	1.62e-02	0.706	0.4120	0.638	*
F4+ vs F3	Master	1.92e-02	0.701	0.4020	-0.782	*
F4+ vs F3	RLE1	3.59e-02	0.681	0.3610	0.683	*
F4+ vs F3	Rare	7.91e-02	0.652	0.3040	0.317	
F4+ vs F3	Singl	1.02e-01	0.642	0.2840	0.446	
F4+ vs F3	Top25	1.50e-01	0.625	0.2500	-0.249	
F3 vs F2	R10	7.11e-01	0.546	0.0910	-0.233	
F3 vs F2	Top25R	8.90e-01	0.519	0.0374	-0.275	

S2.5 Rarefied vs raw non-rarefied quasispecies results

S2.5.1 Median values compared, rarefied versus raw.

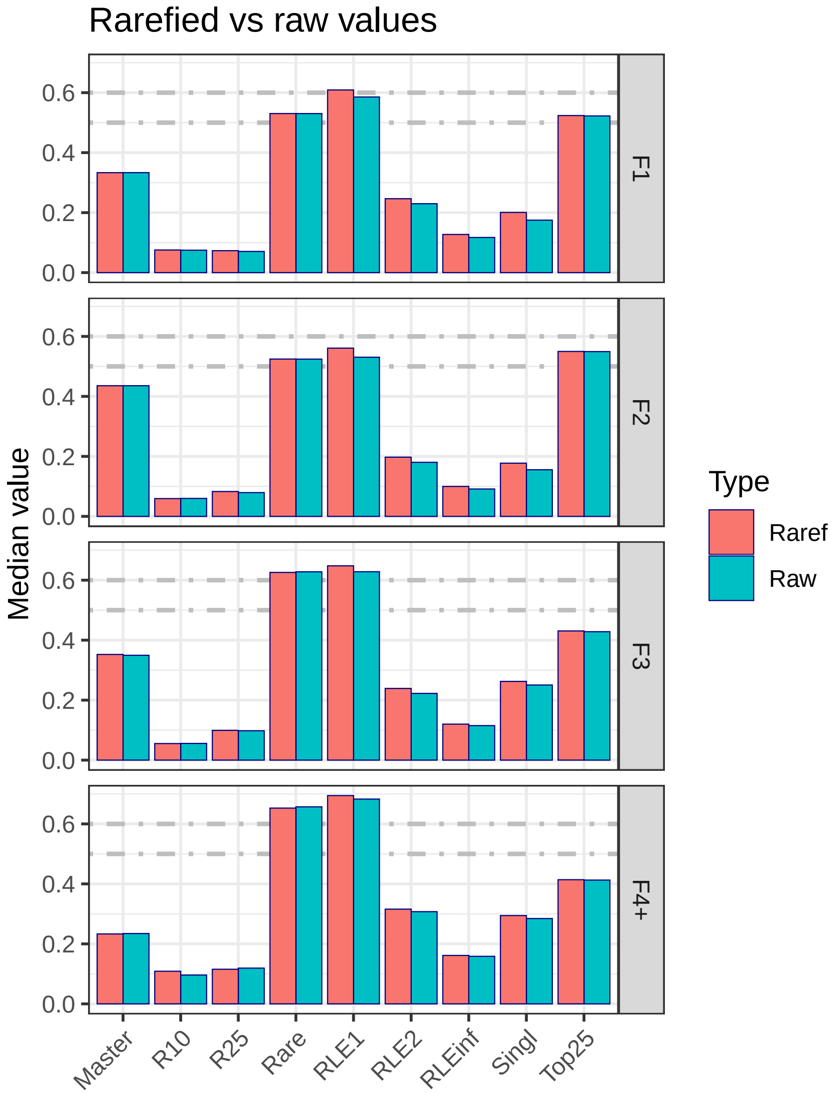

Figure S7: Median values of each indicator per fibrosis level. Plot comparing, rarefied versus raw values.

Relative differences in percentage (Rarefied − Raw)/Rarefied $*$ 100

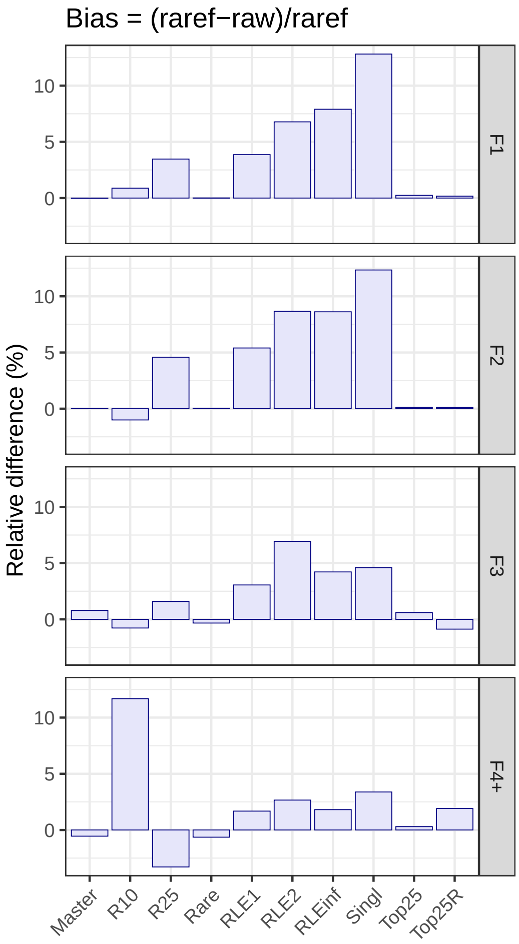

Figure S8: Bias caused by differential coverage, incurred in each indicator, computed as the relative difference (raref-raw)/raref, and expressed as %.

Rarefied values for the indicators Singletons, RLE1, RLE2 and RLE inf are higher than for non rarefied quasispecies. This is the observed effect which causes higher values for lower coverages. Top25, Master, and Rare are almost insensitive to rarefaction, within the limits of this work. The fraction of singletons, despite representing a prominent fraction, is affected by a significant inflation under rarefaction. This is because any resampled singleton in the original sample will be observed as a singleton in the subsample. Additionally, a number of rare haplotypes with more than one read in the original sample will be observed as singletons in the subsampling, causing this inflation. This effect will be more prominent as the higher the number of rare haplotypes observed with few reads in the original sample, and as lower the reference size for rarefaction. See more results about rarefaction impact in the supplementary material in document Suppl_Fibrosis_p0.p100.p200-v10-SortedHplTest+AvM.pdf.

S2.5.2 AUC as effect size. Rarefied versus raw quasispecies.

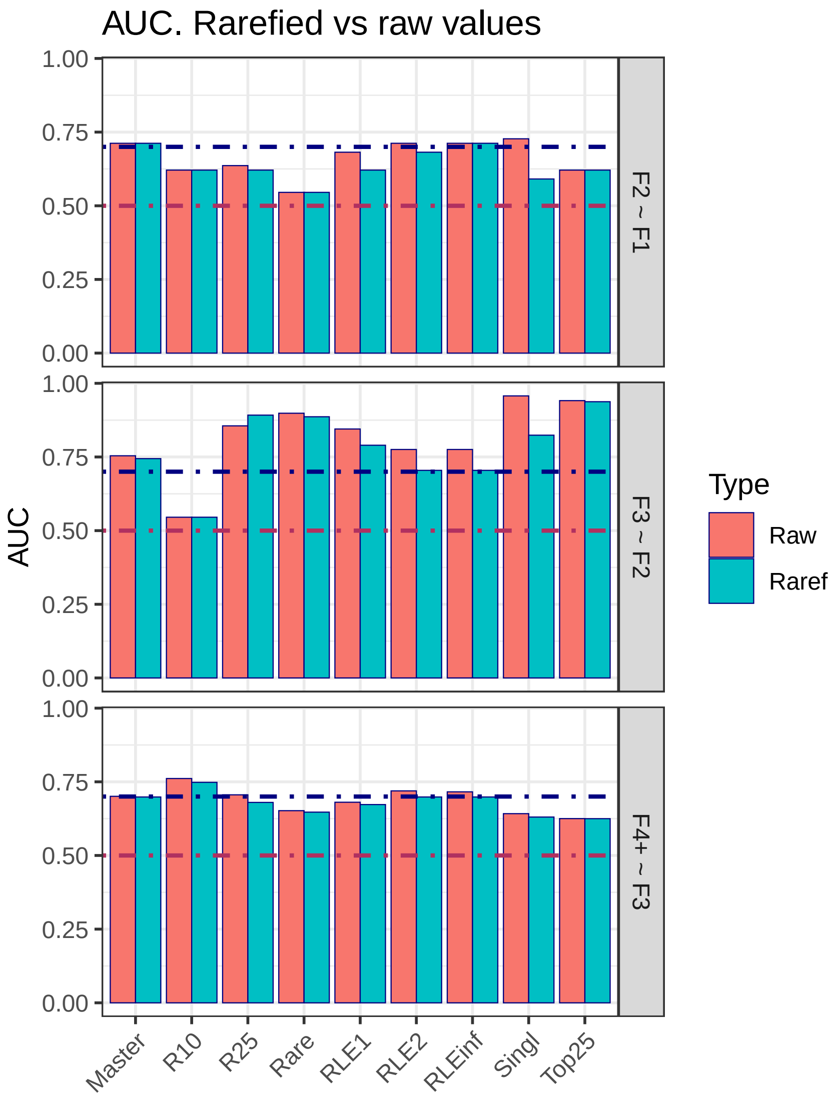

Figure S9: AUC values compared, raw versus rafefied.

The red dash-dot shows the border of aleatority at 0.5. The blue dash-dot line shows the border to a moderate effect, at 0.7.

Table S17: Impact of rarefaction on AUC values. Relative differences expressed in percentage, (raref-raw)/raref.

Test	Indicator	Raw.AUC	Raref.AUC	RelDif
F2 ~ F1	Master	0.7121	0.7121	0.00
F2 ~ F1	Rare	0.5455	0.5455	0.00
F2 ~ F1	Top25	0.6212	0.6212	0.00
F2 ~ F1	Singl	0.7273	0.5909	-23.08
F2 ~ F1	RLE1	0.6818	0.6212	-9.76
F2 ~ F1	RLE2	0.7121	0.6818	-4.44
F2 ~ F1	RLEinf	0.7121	0.7121	0.00
F2 ~ F1	Top25R	0.6667	0.6667	0.00
F2 ~ F1	R10	0.6212	0.6212	0.00
F2 ~ F1	R25	0.6364	0.6212	-2.45
F3 ~ F2	Master	0.7540	0.7443	-1.30
F3 ~ F2	Rare	0.8984	0.8864	-1.35
F3 ~ F2	Top25	0.9412	0.9375	-0.39
F3 ~ F2	Singl	0.9572	0.8239	-16.18
F3 ~ F2	RLE1	0.8449	0.7898	-6.98
F3 ~ F2	RLE2	0.7754	0.7045	-10.06
F3 ~ F2	RLEinf	0.7754	0.7045	-10.06
F3 ~ F2	Top25R	0.5187	0.5170	-0.33
F3 ~ F2	R10	0.5455	0.5455	0.00
F3 ~ F2	R25	0.8556	0.8920	4.08
F4+ ~ F3	Master	0.7008	0.6985	-0.33
F4+ ~ F3	Rare	0.6521	0.6471	-0.77
F4+ ~ F3	Top25	0.6252	0.6250	-0.03
F4+ ~ F3	Singl	0.6420	0.6305	-1.82
F4+ ~ F3	RLE1	0.6807	0.6728	-1.17
F4+ ~ F3	RLE2	0.7193	0.6985	-2.98
F4+ ~ F3	RLEinf	0.7160	0.6985	-2.51
F4+ ~ F3	Top25R	0.7311	0.7298	-0.18
F4+ ~ F3	R10	0.7613	0.7482	-1.75
F4+ ~ F3	R25	0.7059	0.6801	-3.79

Relative differences in AUC, as percentage $(Rarefied - Raw)/Rarefied * 100$

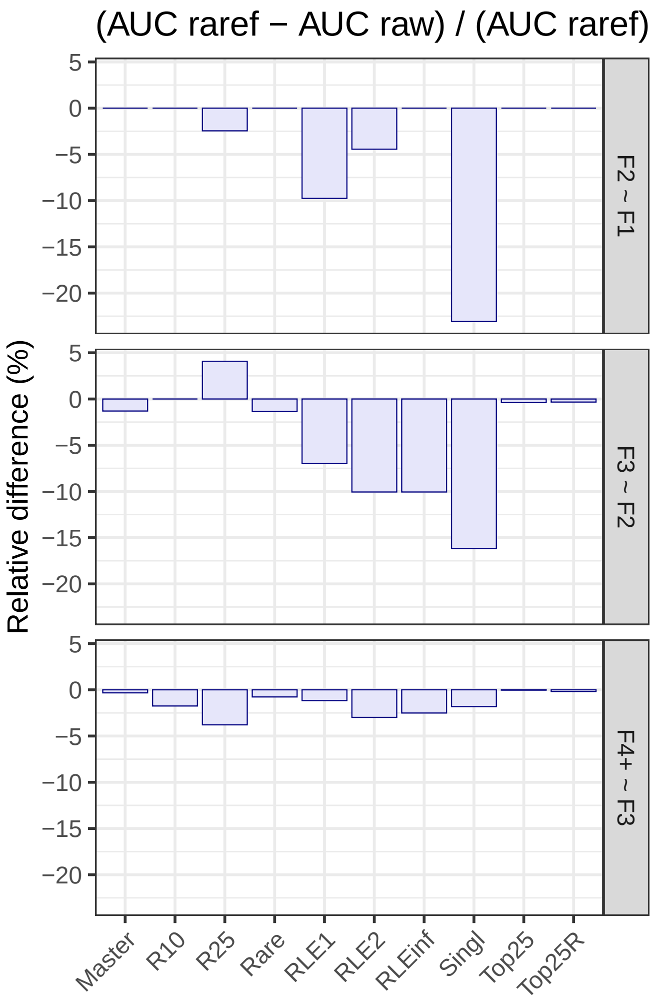

Figure S10: Impact of rarefaction on AUC values. Relative differences expressed in percentage, (raref-raw)/raref.

AUC values computed on rarefied quasispecies show relative lower values with respect to those on raw non-rarefied quasispecies, specially for the evenness indices (RLE1, RLE2, and RLEinf) and for the fraction og singletons. Master, Top25 and Rare show a limited bias below 2% in AUC.

S2.5.3 P-values compared. Rarefied vs raw quasispecies.

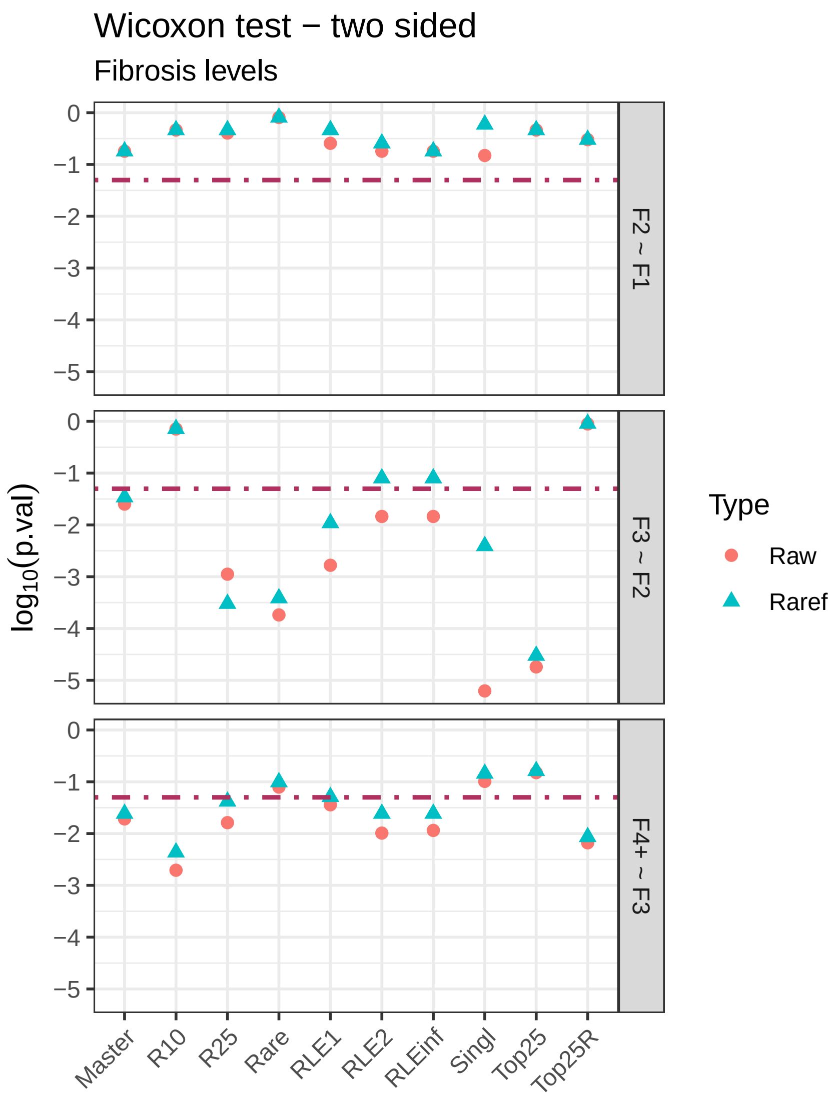

Figure S11: Wilcoxon test p-values on raw and rarefied quasispecies values, p-values plotted in log_{10} scale.

As expected from the coverage differences between fibrosis stages, p-values for the tests on rarefied quasispecies are higher that for non-rarefied quasispecies. With rarefaction, the effect caused by coverage differences is corrected. This is particularly visible in the tests F2 versus F3 fibrosis stages.

Without rarefaction the uncontrolled variable given by coverage takes part in favor of the formulated hypothesis, possibly by chance, and the statistic results obtained are biased towards the hypothesis.

Several indicators are statisticaly significant on rarefied quasispecies, with AUC values over 0.7, specially in F3 vs F2, but also in F4+ vs F3.

Table S18: Raw and rarefied values. Wilcoxon tests results compared through p-values and statistical significance.

Test	Indicator	Raw.p.val	RawSignif	Raref.p.val	RarefSignif
F2 ~ F1	Master	0.1802000		0.1802000	
F2 ~ F1	Rare	0.8075000		0.8075000	
F2 ~ F1	Top25	0.4623000		0.4623000	
F2 ~ F1	Singl	0.1490000		0.5908000	
F2 ~ F1	RLE1	0.2561000		0.4623000	
F2 ~ F1	RLE2	0.1802000		0.2561000	
F2 ~ F1	RLEinf	0.1802000		0.1802000	
F2 ~ F1	Top25R	0.3011000		0.3011000	
F2 ~ F1	R10	0.4623000		0.4623000	
F2 ~ F1	R25	0.4043000		0.4623000	
F3 ~ F2	Master	0.0252200	*	0.0343000	*
F3 ~ F2	Rare	0.0001829	***	0.0003855	***
F3 ~ F2	Top25	0.0000182	***	0.0000299	***
F3 ~ F2	Singl	0.0000062	***	0.0039220	**
F3 ~ F2	RLE1	0.0016630	**	0.0108600	*
F3 ~ F2	RLE2	0.0145600	*	0.0796700	
F3 ~ F2	RLEinf	0.0145600	*	0.0796700	
F3 ~ F2	Top25R	0.8897000		0.9035000	
F3 ~ F2	R10	0.7113000		0.7160000	
F3 ~ F2	R25	0.0011190	**	0.0003007	***
F4+ ~ F3	Master	0.0191900	*	0.0242500	*
F4+ ~ F3	Rare	0.0790600		0.0985900	
F4+ ~ F3	Top25	0.1502000		0.1620000	
F4+ ~ F3	Singl	0.1017000		0.1439000	
F4+ ~ F3	RLE1	0.0359400	*	0.0511300	
F4+ ~ F3	RLE2	0.0102100	*	0.0242500	*
F4+ ~ F3	RLEinf	0.0115000	*	0.0242500	*
F4+ ~ F3	Top25R	0.0066380	**	0.0085990	**
F4+ ~ F3	R10	0.0019600	**	0.0043420	**
F4+ ~ F3	R25	0.0162400	*	0.0417100	*

References

Akinshin, A. 2020. "Nonparametric Cohen's d-Consistent Effect Size." Blog posted 2021-06-08. Last accessed 2024-06-03. https://aakinshin.net/posts/nonparametric-effect-size2/.

Altman, D. G., and J. M. Bland. 1994. "Diagnostic Tests 3: Receiver Operating Characteristic Plots." *BMJ* 309 (6948): 188. https://doi.org/10.1136/bmj.309.6948.188.

Cureton, E. E. 1956. "Rank-Biserial Correlation." *Psychometrika* 21 (3). https://doi.org/10.1007/BF02289138.

Gregori, J., S. Colomer-Castell, M. Ibañez-Lligoña, D. Garcia-Cehic, C. Campos, M. Buti, M. Riveiro-Barciela, et al. 2024. "In-Host Flat-Like Quasispecies: Characterization Methods and Clinical Implications." *Microorganisms* 12 (5): 1011. https://doi.org/10.3390/microorganisms12051011.

Gregori, J., M. Ibañez-Lligoña, S. Colomer-Castell, C. Campos, and J. Quer. 2024. "Virus Quasispecies Rarefaction: Subsampling with or Without Replacement?" *Viruses* 16 (5): 710. https://doi.org/10.3390/v16050710.

Hanley, J. A., and B. J. McNeil. 1982. "The Meaning and Use of the Area Under a Receiver Operating Characteristic (ROC) Curve." *Radiology* 143 (1): 29–36. https://doi.org/10.1148/radiology.143.1.7063747.

Lötsch, J., and A. Ultsch. 2020. "A Non-Parametric Effect-Size Measure Capturing Changes in Central Tendency and Data Distribution Shape." *PLoS One* 15 (9). https://doi.org/10.1371/journal.pone.0239623.

Sawilowsky, S. 2009. "New Effect Size Rules of Thumb." *Journal of Modern Applied Statistical Methods*, no. 2. https://doi.org/10.22237/jmasm/1257035100.

Zweig, M. H., and G. Campbell. 1993. "Receiver-Operating Characteristic (ROC) Plots: A Fundamental Evaluation Tool in Clinical Medicine." *Clin Chem.* 39 (4). https://doi.org/10.1093/clinchem/39.4.561.
