# Supplementary material for: Association of Liver Damage and Quasispecies Maturity in Chronic HCV Patients: The Fate of a Quasispecies
Source: Microorganisms. 2024 Oct 31;12(11):2213. doi: 10.3390/microorganisms12112213 (PMC11596025; doi:10.3390/microorganisms12112213)
Supplement: Supplementary file 1 [file microorganisms-12-02213-s001.zip › Supplementary material cell culture data.pdf]

Association of liver damage and quasispecies maturity in chronic HCV patients: The fate of a quasispecies

Suppl. Material: Quasispecies maturity in cell culture

Josep Gregori[*], Marta Ibañez-Lligoña, Sergi Colomer-Castell, Carolina Campos, Damir García-Cehic, and Josep Quer[†]

2024-10-08

Introduction and background

The data used in this study is taken from (Gregori et al. 2018) and is core data for a set of publications leaded by Prof. Esteban Domingo's team in a research line that, based on cell culture results, uncovered quasispecies fitness as a determinant of treatment response, including classical treatments (IFN-α, telaprevir, daclatasvir, cyclosporine, and ribavirin) (Sheldon et al. 2014), mutagenic agents (ribavirin and favipiravir) (Gallego et al. 2018), and high phenotypic barrier to resistance DAAs such as Sofosbuvir (Gallego et al. 2016). The following is a non-exhaustive list of these publications:

- *Increased Replicative Fitness Can Lead to Decreased Drug Sensitivity of Hepatitis C Virus* (Sheldon et al. 2014)
- *Barrier-Independent, Fitness-Associated Differences in Sofosbuvir Efficacy against Hepatitis C Virus* (Gallego et al. 2016)
- *Internal Disequilibria and Phenotypic Diversification during Replication of Hepatitis C Virus in a Noncoevolving Cellular Environment* (Moreno et al. 2017)
- *Resistance of high fitness hepatitis C virus to lethal mutagenesis* (Gallego et al. 2018)
- *Broad and Dynamic Diversification of Infectious Hepatitis C Virus in a Cell Culture Environment* (Gallego et al. 2020)
- *A new implication of quasispecies dynamics: Broad virus diversification in absence of external perturbations* (Domingo et al. 2020)
- *Population Disequilibrium as Promoter of Adaptive Explorations in Hepatitis C Virus* (García-Crespo et al. 2021)
- *Historical Perspective on the Discovery of the Quasispecies Concept* (Domingo, García-Crespo, and Perales 2021)

[*]josep.gregori@gmail.com
[†]josep.quer@vhir.org

- *Viral Fitness, Population Complexity, Host Interactions, and Resistance to Antiviral Agents* (Domingo et al. 2023)

The experimental designs of serial virus passages in cell culture (HCV replicating in human hepatoma Huh-7.5 cells) imposed the exclusion of external selective constraints, thus allowing unperturbed and prolonged virus multiplication. Despite no selective pressure, evolution is granted by two factors: new mutations produced by the low replicating fidelity of NS5B, and continuous selection of variants driven by quasispecies internal fitness gradients.

Analysis of mutant spectrum composition, quasispecies diversity and fitness after 45, 100 and 200 passages brought evidence of an adaptive feature of RNA viruses that was termed *"broadly diversifying selection"* (Domingo et al. 2020), it constitutes a new type of positive selection without participation of any external selective agent, built upon a progressive increase of the number of different genomes that dominate the population coupled with an increase in quasispecies fitness (Domingo et al. 2020). Resistance to the different treatments was observed despite no prior exposure to these drugs, and no drug resistance mutations found. This discovery underlines the fate of a quasispecies

This supplementary material revisits the data from passages 0, 100 and 200 (p0, p100, p200) in light of the indicators used in the main text (Gregori, Ibañez-Lligoña, Colomer-Castell, Garcia-Cehic, et al. 2024) with patient data, using a NS5B amplicon spanning positions 8552:8869. The results of the analyses on the p0, p100, and p200 data are presented and discussed in the context of the findings of the above cited works, (Gregori, Colomer-Castell, et al. 2024), and from the main text. The association between infection time and quasispecies diversification and fitness is explored under the hypothesis that a quasispecies will tend to a flat-like quasispecies structure in its exploration of the genetic space.

A quasispecies matures as it explores the genetic space, primarily due to the low replication fidelity of RNA-dependent RNA polymerases. This process involves the incorporation of new, better-fitted variants into the quasispecies while less-fitted variants are purged, both driven by fitness gradients, even in the absence of external selective pressures (Domingo et al. 2020; Gallego et al. 2020). As the quasispecies 'matures,' it grows in complexity, following a natural tendency to evolve towards flat-like fitness landscapes with higher mean quasispecies fitness. This evolution is facilitated by the vast number of synonymous substitutions and neutral mutations, which provide the pathways along which the quasispecies traverses the genetic space. Ultimately, this maturation process enables the quasispecies to dominate a broader range of the genetic landscape (Gregori, Colomer-Castell, et al. 2024).

Methods

Quasispecies maturity indicators

The next table shows the expected level of each selected quasispecies indicator in the two limiting cases, regular vs flat-like quasispecies (Gregori, Colomer-Castell, et al. 2024):

Indicator	Description	Regular Qs	Flat-like Qs
Master	Dominant haplotype frequency	High	Low
Top25	Fraction of reads for top 25 hpl.	High	Low
Rare1	Fraction of reads for hpl $\leq 1\%$	Low	High
Rare2	Fraction of reads for hpl $\leq 0.1\%$	Low	High
Singl	Fraction of singletons	Low	High
RLE_1	Relative logarithmic evennes at $q = 1$	Low	High
RLE_2	Relative logarithmic evennes at $q = 2$	Low	High
RLE_∞	Relative logarithmic evennes at $q = \infty$	Low	High
R5	Evenness among top 5 haplotypes	Low	High
R10	Evenness among top 10 haplotypes	Low	High
R25	Evenness among top 25 haplotypes	Low	High

Relative logarithmic evenness

$$RLEq = RLE(p,q) = \log_{10}(D(p,q))/\log_{10}(D(p,0)) \tag{1}$$

and $D(q,p)$ the Hill number of order q.

$$D(p,q) = \left(\sum_{i=1}^{H} p_i^q\right)^{1/(1-q)} \tag{2}$$

H number of haplotypes, $p = (p_1, p_2, \ldots, p_H)$ the set of haplotype frequencies.

Fraction of top haplotypes

On the set of haplotype frequencies sorted in decreasing order, $p_1 \geq p_2 \geq \cdots \geq p_H$, the following indicators are computed:

$$\text{Top25}(p) = \left(\sum_{i=1}^{m} p_i \;\middle|\; m = \min(25, H)\right) \tag{3}$$

$$\text{Top25R}(p) = \frac{\text{Top25}(p)}{p_1} \tag{4}$$

Evenness on the set of top haplotypes

An indicator of evenness on the set of k top haplotypes is computed as:

$$R_k(p) = \left(\frac{l \cdot p_l}{\sum_{i=1}^{l} p_i} \;\middle|\; l = \min(k, H)\right) \tag{5}$$

Permutation tests

To test the differences in quasispecies maturity indicators between two quasispecies, a permutation test (Moore 1999) is coded encompassing the following steps:

1. Calculate the indicators for the original two quasispecies samples, rarefying the bigger sample to the size of the smaller. Compute the difference between these indicators as the observed test statistic.
2. Inside a resampling loop for 1000 permutations to generate a null distribution of differences in diversity:
    i. Get rarefied haplotype counts of the bigger sample
    ii. Assign labels to the haplotypes in each quasispecies based on their abundance, in descending order, independently for each quasispecies regardless of whether the haplotypes are the same or different between the samples. The focus is on the abundance patterns rather than the specific haplotype identities.
    iii. Pool together the reads of both quasispecies.
    iv. Randomly shuffle the pooled reads and reassign half of them to each quasispecies.
    v. Calculate the diversity indices for these permuted groups and their difference.
3. Compare the observed difference to this null distribution to obtain a p-value.
4. Calculate the Standardized Permutation Statistic (SPS), Z, as the observed difference divided by the standard deviation of the permuted differences.

$$Z = \frac{\text{Observed Difference}}{\text{SD(Permuted Differences)}} \tag{6}$$

The Z statistic expresses the effect size in terms of the standard deviation of the permuted differences, offering a standardized measure that helps to interpret permutation p-values which resulted as 0, and shows a measure of effect size associated with the null distribution. Finally, the observed permutation *p.value*. .

The SPS (Standardized Permutation Statistic) is a statistic analogous to a Cohen's d effect size, in terms of the null distribution standard deviation, that provides a measure of the magnitude of the effect. Given the inherent limitations in the calculation of permutation p-values, which are bounded by the number of permutations and may result in values of 0, the SPS offers a continuous and more precise measure of the effect's magnitude and direction, allowing for easier interpretation. P-values of 0 can occur in permutation tests when the observed statistic is more extreme than all permuted statistics, a situation where the SPS remains informative. When 0, these p-values are reported as $< 1/n_p$, with n_p the number of permutations.

This analysis employs permutation tests to compare selected indicators between rarefied quasispecies samples: p0.a2.fw vs. p100.a2.fw, and p100.a2.fw vs. p200.a2.fw. The observed values for each sample are derived from 200 rarefaction cycles, with the median value selected. The permutation process involves 1000 reshuffling cycles to obtain an empirical null distribution. In each cycle, a single rarefaction is performed to pool the reads from both quasispecies to be reshuffled. This approach ensures that the permutation test accounts for potential biases due to varying sequencing depths while maintaining the overall characteristics of the data. By comparing rarefied samples and employing a large number of permutations, this method provides a robust statistical framework for assessing differences in quasispecies populations across different time points or conditions.

Th R code (R Core Team 2024) is given in the appendix.

Results

We report the characterization of an HCV evolving quasispecies in cell culture with relative quasispecies fitness increases (p0 1., p100, 2.3 and p200 2.3) (Domingo et al. 2023).

Samples, amplicons, and coverage

Basal samples are p0, p100 and p200. To account for variations two samples for each basal level are analyzed, at pass 0 and at pass 3 (p0, p3). This p3 means three extra passes after the basal level. One NS5B amplicon spanning positions 8552:8869 is analyzed for each sample.

Despite some variations, the coverage of all samples is largely consistent.

Table 2: Reads per amplicon sample and strand

Basal	Pass	FW	RV
p0	p0	124253	120939
p0	p3	186398	182387
p100	p0	179401	176168
p100	p3	203067	193848
p200	p0	123807	123946
p200	p3	120611	119136

Quasispecies maturity indicators, raw values

Values of these indicators are first computed on the quasispecies as they are observed, without size normalization. The values shown are averaged over the two strands of each amplicon.

Table 3: Quasispecies maturity indicators. Mean raw values per amplicon.

ID	nHpl	Master	Top25	Rare1	Rare2	Singl
p0.p0	5720	0.728	0.801	0.242	0.198	0.0362
p0.p3	8350	0.730	0.786	0.270	0.191	0.0375
p100.p0	11700	0.491	0.744	0.331	0.230	0.0506
p100.p3	14000	0.430	0.742	0.308	0.230	0.0520
p200.p0	11300	0.435	0.708	0.370	0.257	0.0682
p200.p3	11700	0.481	0.698	0.385	0.252	0.0765

ID	R5	R10	R25	RLE1	RLE2	RLEinf
p0.p0	0.0252	0.0238	0.0323	0.278	0.0731	0.0366
p0.p3	0.0217	0.0293	0.0482	0.274	0.0698	0.0349
p100.p0	0.0603	0.0675	0.0643	0.382	0.1440	0.0758
p100.p3	0.2110	0.0678	0.0545	0.405	0.1680	0.0885
p200.p0	0.1900	0.0761	0.0711	0.436	0.1710	0.0892
p200.p3	0.1000	0.0911	0.0831	0.429	0.1540	0.0781

Maturity indicator values on rarefied quasispecies

Next, quasispecies are submmitted to a size normalization by rarefaction (Gregori, Ibañez-Lligoña, Colomer-Castell, Campos, et al. 2024). Each combination of amplicon/strand, representing a quasispecies, will be submitted to a rarefaction process to compute all indicators to a common reference size of 100,000 reads. 500 cycles of rarefaction are executed, for each amplicon/strand, and at each cycle all indicators are computed. Finally the rarefied values are taken as the median of the 500 values. For each amplicon the mean of the two strand values is computed.

Table 5: Quasispecies maturity indicators. Mean rarefied values per amplicon.

ID	nHpl	Master	Top25	Rare1	Rare2	Singl
p0.p0	4890	0.728	0.801	0.242	0.197	0.0371
p0.p3	5090	0.730	0.786	0.270	0.190	0.0398
p100.p0	7610	0.491	0.744	0.331	0.229	0.0562
p100.p3	8440	0.430	0.742	0.308	0.230	0.0614
p200.p0	9620	0.435	0.708	0.370	0.257	0.0716
p200.p3	10100	0.481	0.698	0.385	0.252	0.0791

ID	R5	R10	R25	RLE1	RLE2	RLEinf
p0.p0	0.0253	0.0238	0.0321	0.282	0.0745	0.0373
p0.p3	0.0219	0.0296	0.0475	0.286	0.0738	0.0369
p100.p0	0.0602	0.0668	0.0637	0.396	0.1510	0.0795
p100.p3	0.2110	0.0677	0.0546	0.423	0.1780	0.0934
p200.p0	0.1900	0.0763	0.0717	0.441	0.1740	0.0908
p200.p3	0.1000	0.0914	0.0829	0.434	0.1560	0.0793

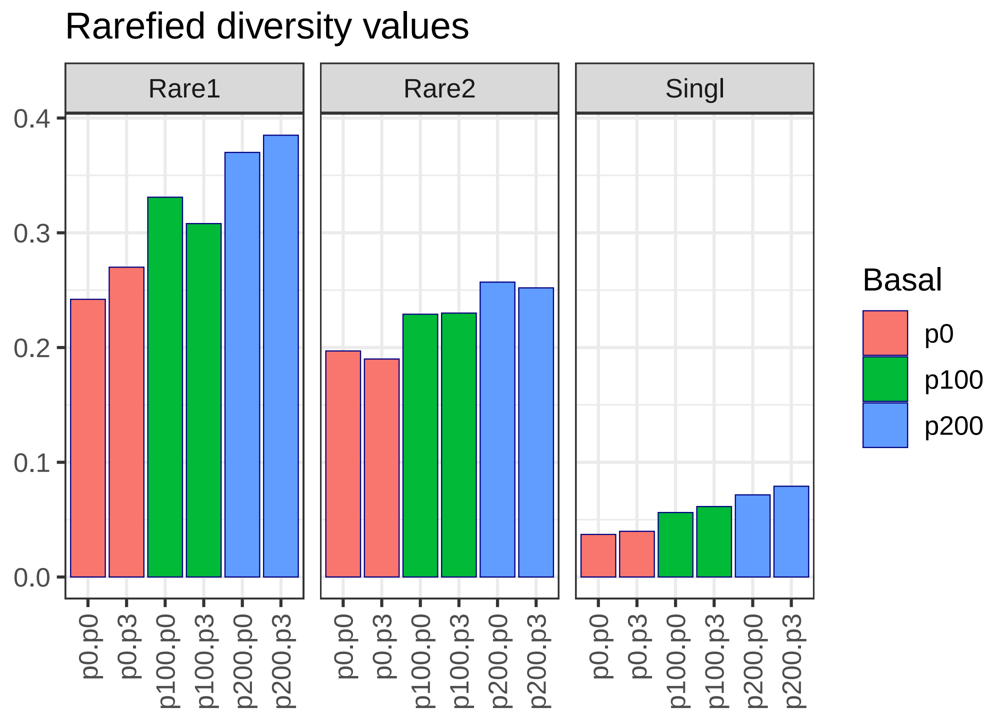

Figure 1: Rarefied values of the fractions of reads for rare haplotypes. Rare1 (<1%), Rare2 (<0.01%) and Singletons.

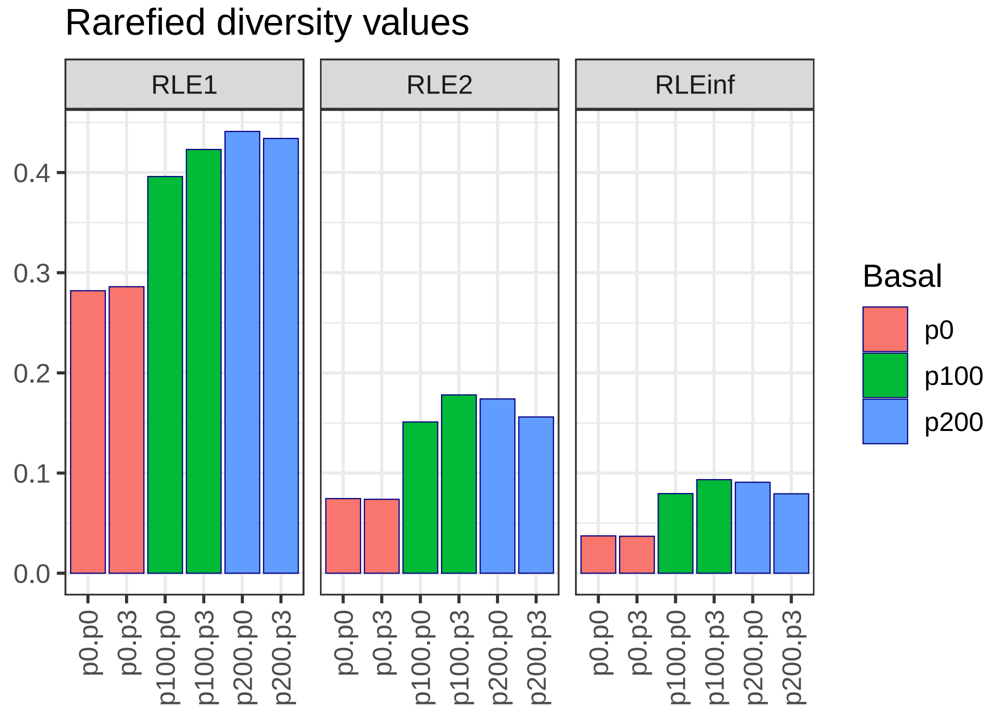

Figure 2: Rarefied values of evenness indices, RLE1, RLE2, and RLEinf.

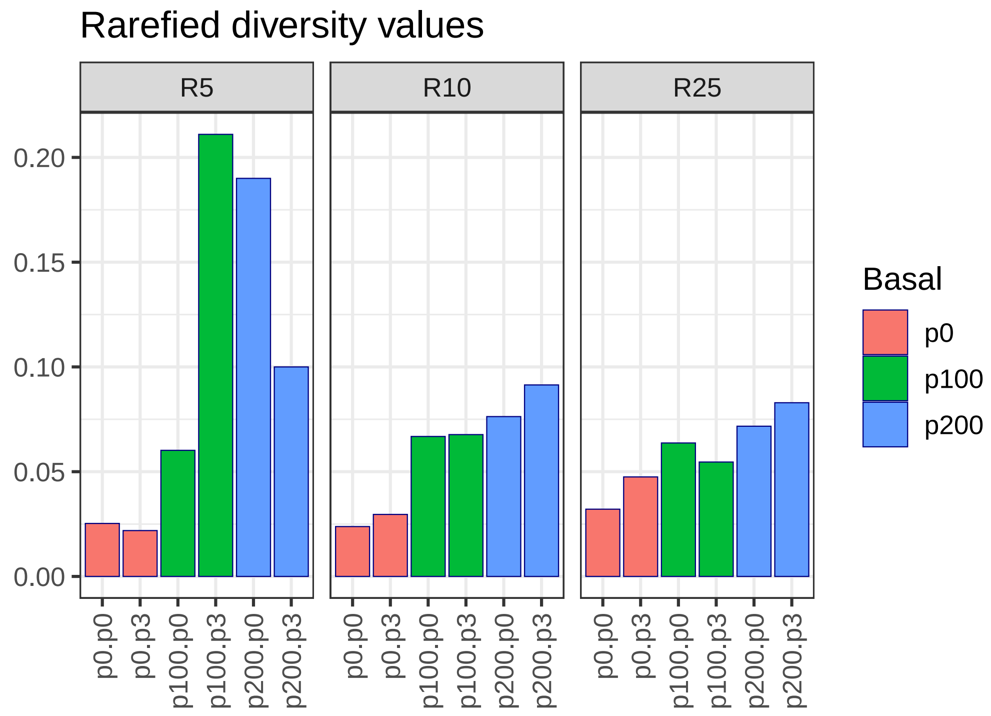

Figure 3: Rarefied values of top haplotypes evenness, R10 and R25.

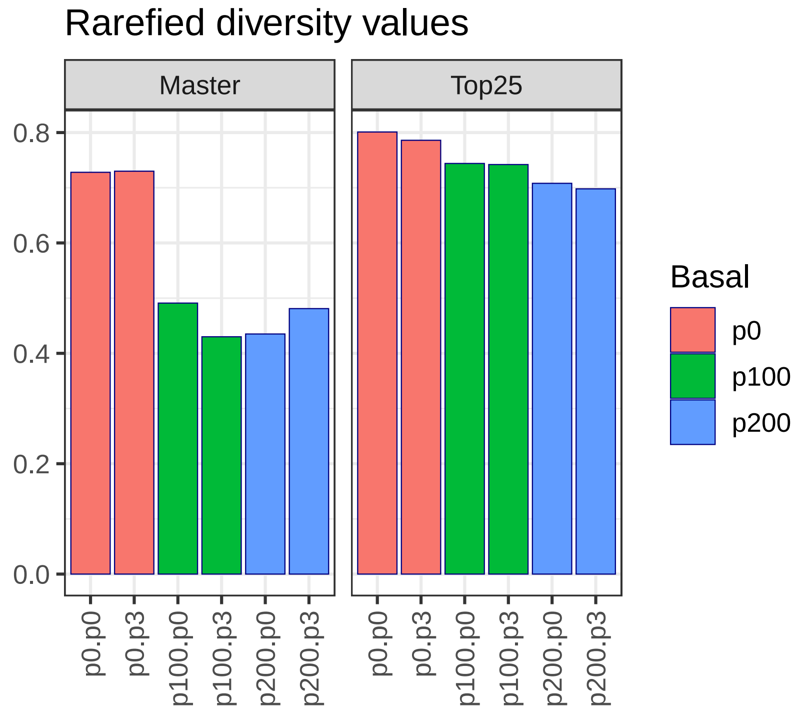

Figure 4: Rarefied values of Master and Top25 haplotypes fraction.

Mean values per basal level (p0, p100 and p200).

To smooth the variations caused by multiple uncontrolled factors from wet-lab to sequencing, the values of passes p0 and p3 for each basal p0, p100 and p200 are averaged.

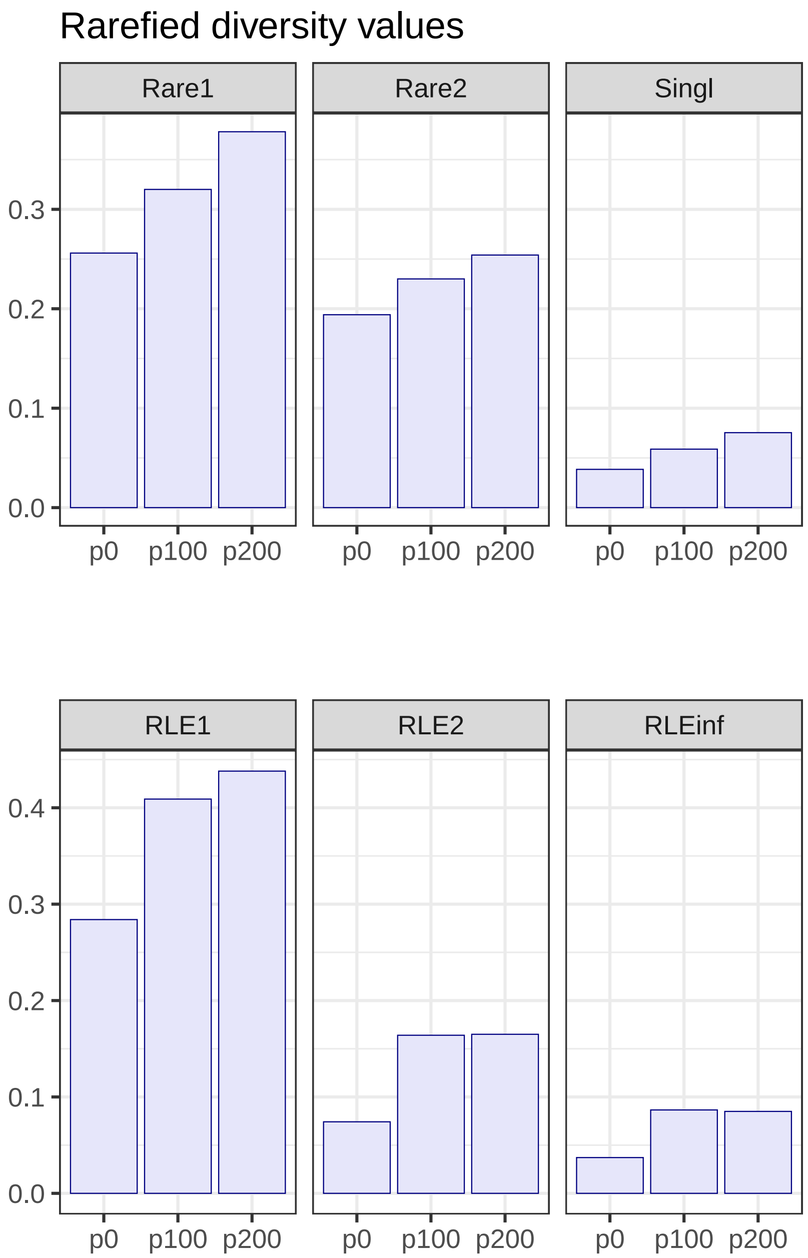

Figure 5: Averaged p0 and p3 rarefied values of the fractions of reads for rare haplotypes, and relative logarithmic evenness (RLE) rarefied values, averaged for p0 and p3 of each sample.

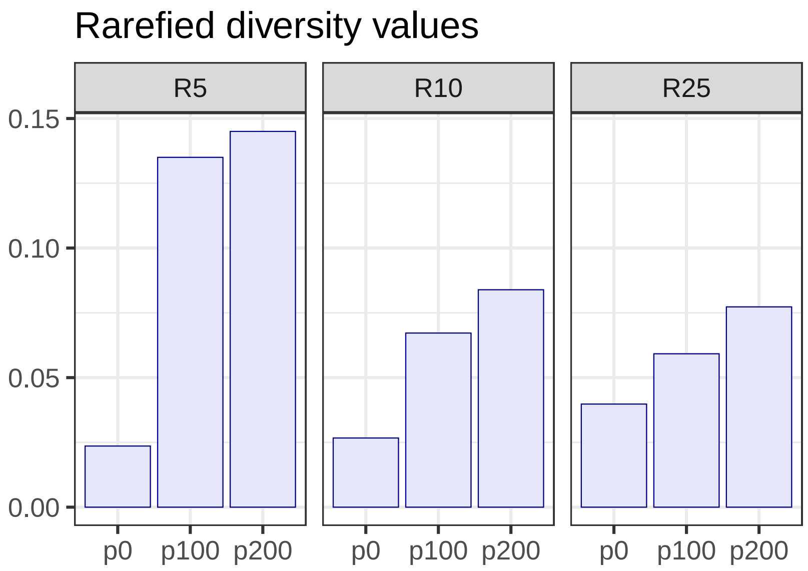

Figure 6: Top haplotypes evenness rarefied values, averaged for p0 and p3 of each sample.

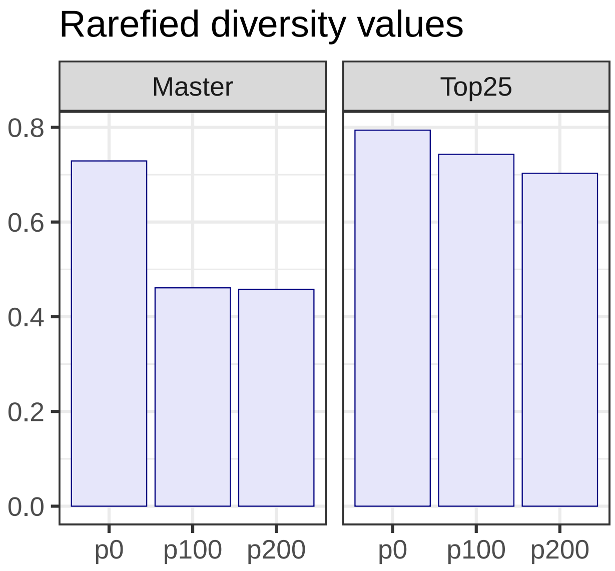

Figure 7: Master and Top25 haplotypes fraction rarefied values, averaged for p0 and p3 of each sample.

Impact of rarefaction on each index

Heterogeneity in sample sizes can introduce significant biases in the downstream analysis, as the observed diversity and abundance patterns may be influenced more by the differences in sequencing depth rather than the actual biological differences between the samples. Rarefaction is a widely used normalization method in such cases (Gregori, Ibañez-Lligoña, Colomer-Castell, Campos, et al. 2024). It involves subsampling each sample to a common, lower sequencing depth, effectively equalizing the number of reads across all samples. It helps to mitigate the differential biases.

Next table and figure show the observed impact of rarefaction on each studied index, which help in the interpretation of results.

Table 7: Relative difference (raw-raref)/raref as %

Ind	Min	Median	Max
nHpl	14.65025	35.73011	67.74527
Singl	-16.09160	-5.22804	-2.39710
RLE2	-5.53604	-3.24400	-1.46317
RLEinf	-5.53660	-3.24329	-1.46313
RLE1	-4.50108	-2.57280	-1.07823
R25	-2.61561	0.34583	2.94790
R10	-1.23896	-0.07434	1.91581
R5	-1.59607	-0.05123	0.10213
Top25	-0.03566	-0.00708	0.00363
Rare2	-0.44109	-0.00657	1.01551
Master	-0.00957	0.00193	0.03025
Rare1	-0.02734	0.00156	0.02168

Different types of effects are observed:

- Haplotypes: Is the most heavily affected index, with a 35.7% median relative difference, and a maximum of 67.7%. Rare haplotypes in highly diverse quasispecies are severely affected by subsampling.
- Prominent fractions: The prominent fractions of the quasispecies, represented by "Master", "Top25", and "Rare1", are almost unaffected by rarefaction. This is because they constitute a significant portion of the overall population and are not significantly impacted by the subsampling process.
- Positive and negative differences: The "Rare2", "R10", and "R25" indices show a mix of positive and negative differences under rarefaction. This indicates that the impact of rarefaction on these components of the quasispecies is more variable and can result in both increases and decreases in their relative abundances.
- Evenness indices: The evenness indices "RLE1", "RLE2", and "RLEinf" show higher values under rarefaction. This can be explained by the significant impact of rarefaction on the number of haplotypes, which decreases by a median of 35.7%. As the number of haplotypes is reduced, the evenness of the remaining haplotypes increases, leading to the observed higher evenness index values.

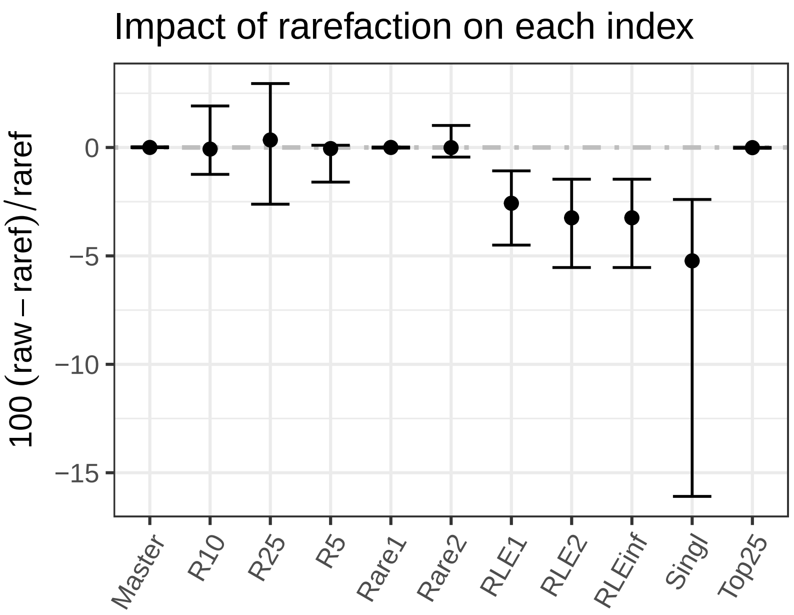

Figure 8: Effects of rarefaction on each index. Min - Median - Max relative differences (%)

- Singletons: The fraction of singletons, despite representing a prominent fraction, is affected by a significant inflation under rarefaction. This is because any resampled singleton in the original sample will be observed as a singleton in the subsample. Additionally, a number of rare haplotypes with more than one read in the original sample will be observed as singletons in the subsampling, causing this inflation. This effect will be more prominent as the higher the number of rare haplotypes observed with few reads in the original sample, and as lower the reference size for rarefaction.

Rarefaction, as a normalization technique, simulates the effects of incomplete sampling by subsampling the original data to a lower sequencing depth. This process mimics the scenario where the sample size is not large enough to capture the full diversity of the population, and the resulting patterns observed under rarefaction can be considered analogous to the biases and limitations encountered in real-world situations with insufficient sampling.

While rarefaction enables fair comparisons, it does come with a trade-off. By subsampling the larger samples to match the sequencing depth of the smaller samples, rarefaction effectively “penalizes” the larger samples in favor of the less represented ones.

Establishing a minimum acceptable sequencing coverage, or depth, is a crucial step in the experimental design process for studies involving the analysis of quasispecies. By setting the minimum acceptable coverage, researchers can effectively define the level of information that the experiment will convey. This minimum coverage threshold ensures that the samples collected and analyzed provide a sufficient level of information to draw meaningful conclusions from the data. Samples that fall below the minimum acceptable coverage should either be repeated to increase the sequencing depth or rejected from the analysis altogether.

Principal Components Analysis

Plotting a multidimensional dataset with class labels on the PC1/PC2 plane from Principal Component Analysis (PCA) provides a powerful visualization tool, and results highly informative when the variance explained by these two components accounts for a high fraction of the total variation. In our case PC1 accounts for 89.1% of the total variance, and PC2 for an extra 8.6%. The high correlation between quasispecies maturity indicators ensures this result.

Table 8: Proportion of variance for top components in PCA.

	PC1	PC2	PC3
Standard deviation	3.130803	0.9744634	0.399974
Proportion of Variance	0.891080	0.0863300	0.014540
Cumulative Proportion	0.891080	0.9774100	0.991950

The scatterplot shows how each sample is placed with respect to each other in terms of quasispecies diversification.

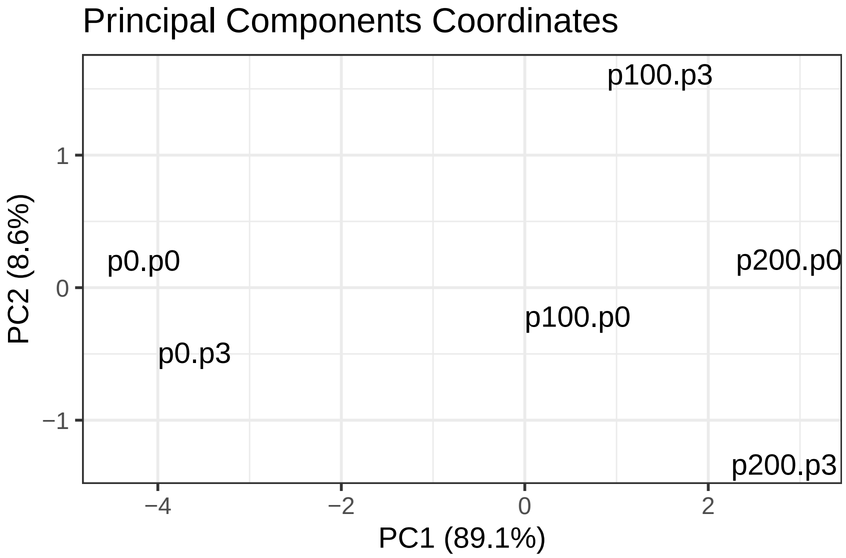

Figure 9: Scatterplot of samples on the PC1/PC2 plane.

The PC1 coefficients align with expected trends. Higher Master and Top25 values characterize regular quasispecies profiles, typically indicating viral populations with one or few dominant haplotypes. Conversely, higher values in diversity, evenness, and rare variant load metrics are associated with highly diverse quasispecies profiles, reflecting more heterogeneous viral populations.

Table 9: Coefficients of first two principal components.

	PC1	PC2
Master	-0.3066	-0.2459
Top25	-0.3111	0.2149
Rare1	0.3002	-0.3377
Rare2	0.3116	-0.0848
Singl	0.3097	-0.1865
R5	0.2467	0.5882
R10	0.3129	-0.1575
R25	0.2865	-0.4341
RLE1	0.3177	0.0910
RLE2	0.3053	0.2786
RLEinf	0.3018	0.3036

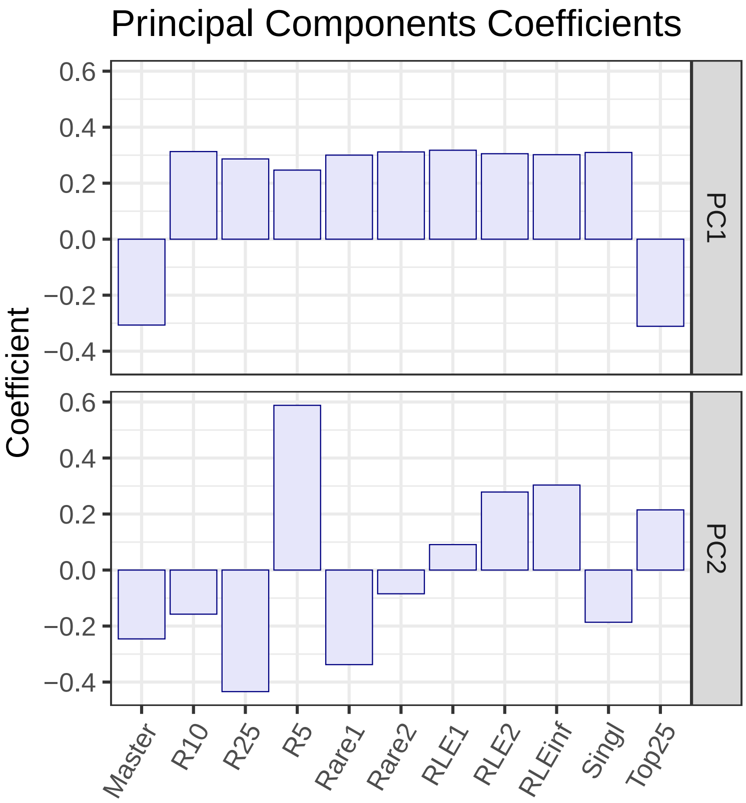

Figure 10: Coefficients of first two principal components.

Permutation tests

Test p0 vs p100

In a concise summary, the results for each tested indicator encompass several key statistics. The observed values for the two populations are denoted as $p0$ and $p100$, with their difference represented by *Obs.Diff*. To contextualize these observations within the permutation framework, *Perm.Med* provides the median of the differences between the indicators across all permutations under the null hypothesis of equality. The Z statistic expresses the effect size in terms of the standard deviation of the permuted differences, offering a standardized measure that helps to interpret permutation p-values which resulted as 0, and shows a measure of effect size associated with the null distribution. Finally, the permutation *p.value*. When 0, these p-values are reported as $< 1/n_p$, whith n_p the number of permutations. That is $< 1.e - 3$. Together, these statistics offer a comprehensive assessment of the differences between the two quasispecies and their statistical relevance.

	p0	p100	Obs.Diff	Perm.Med	Z	p.value
Master	0.72884	0.49213	-0.23671	0.00014	-122.4	0
Top25	0.80131	0.74291	-0.05840	0.00020	-35.2	0
Rare2	0.19969	0.23110	0.03141	-0.00028	13.0	0
R10	0.02458	0.06609	0.04151	0.00010	18.0	0
RLE1	0.27677	0.39084	0.11406	-0.00014	75.1	0
RLEinf	0.03646	0.07783	0.04137	-0.00002	118.1	0

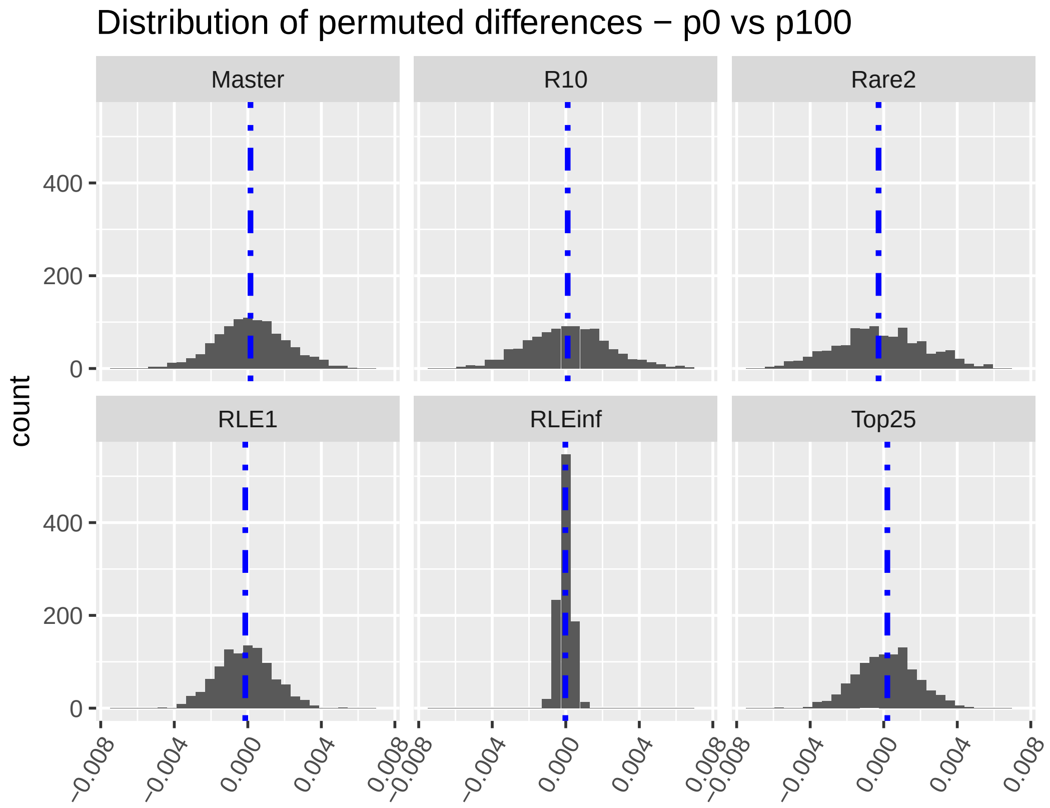

Test p100 vs p200

	p100	p200	Obs.Diff	Perm.Med	Z	p.value
Master	0.49211	0.43506	-0.05705	0.00000	-28.2	0
Top25	0.74291	0.70577	-0.03715	-0.00002	-20.6	0
Rare2	0.23073	0.25735	0.02662	-0.00013	9.4	0
R10	0.06619	0.08438	0.01819	0.00011	4.8	0
RLE1	0.39095	0.43750	0.04656	0.00004	30.6	0
RLEinf	0.07786	0.08902	0.01116	0.00000	23.7	0

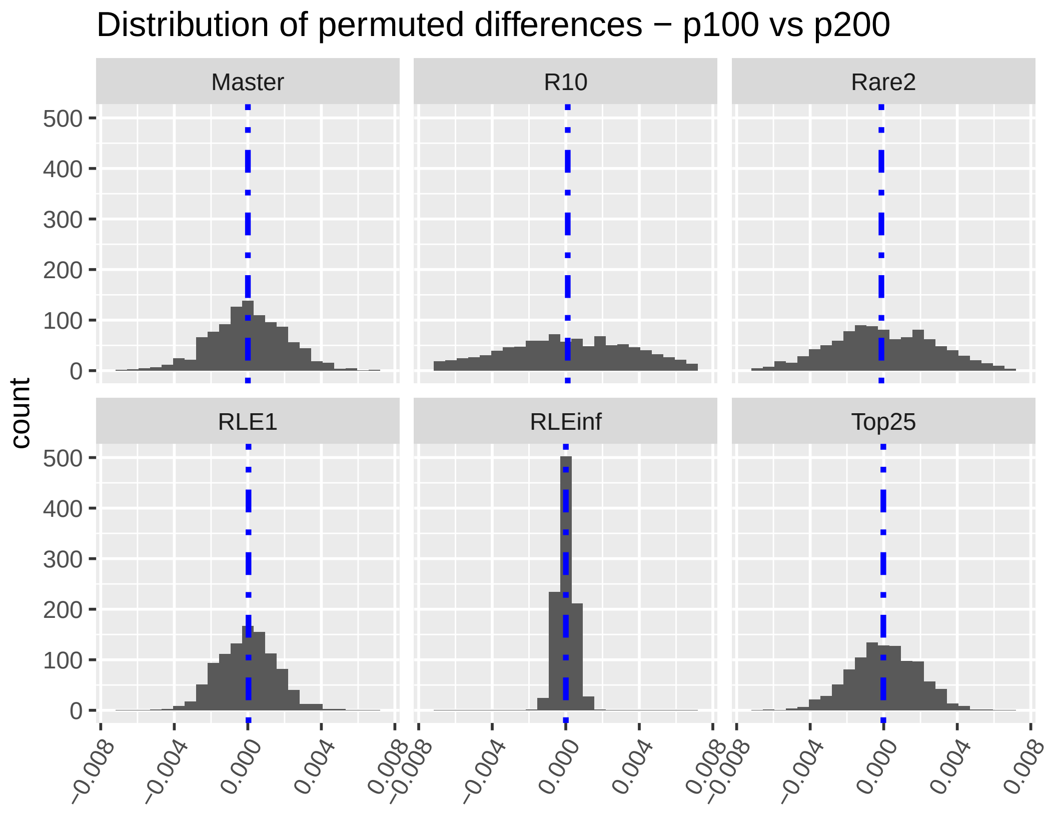

As a guide to interpret the SPS Z statistic, we can draw an analogy to the normal distribution: a z-score of 5 in a normal test corresponds to a single-tail p-value of 2.87e-07, and a z-score of 10 to a p-value of 7.62e-24. While the permutation distribution may not exactly follow a normal distribution, this comparison provides a useful reference.

Synonymity

The maturation process implies the incorporation of several haplotypes expressing a reduced set of functional phenotypes, in order to maintain the virus functionality within a high genomic diversity. This feature corresponds to a high level of haplotype synonymity in the quasispecies. This characteristic may be measured by the ratio of the number of haplotypes to the number of phenotypes, Rn, and the ratio of the frequencies of the master phenotype to the master haplotype, RMstr.

Table 12: Quasispecies synonymity

Pat.ID	nHpl	nPhn	Rn	HplMstr	PhnMstr	RMstr
p0.Ctl.p0	5290	2799	1.890	0.736	0.824	1.120
p0.Ctl.p3	7622	3816	1.997	0.739	0.838	1.135
p100.Ctl.p10	9068	3448	2.630	0.410	0.567	1.385
p100.Ctl.p3	13306	5095	2.612	0.432	0.593	1.371
p200.Ctl.p10	8623	3352	2.572	0.561	0.746	1.329
p200.Ctl.p3	10456	4092	2.555	0.504	0.701	1.393

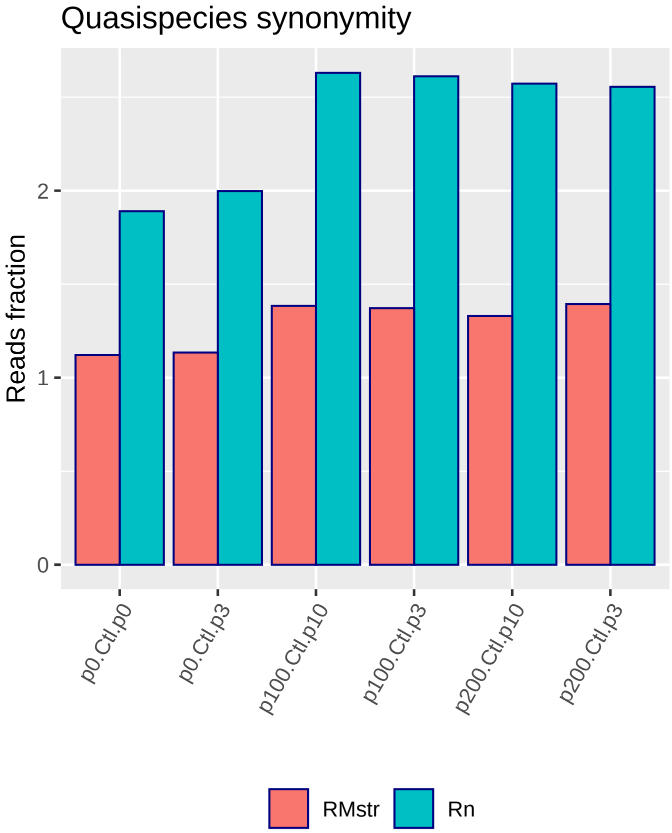

Figure 11: Quasispecies synonymity. Rn, ratio haplotypes/phenotypes; RMstr, ratio master phenotype to master haplotype frequency.

Explored genetic space

If the center of a quasispecies genome cloud is the master haplotype, which is the currently most frequent haplotype in the quasispecies, the amount of genetic space explored by the quasispecies can be evaluated by the mean number of differences (substitutions) per read with respect to the master haplotype in each amplicon. This is observed in a pairwise alignment of all haplotypes relative to the master. This approach provides a measure of genetic distance from the center, illustrating how far the quasispecies has expanded across the genetic space. It's important to note that this does not imply all substitutions were produced from the current master haplotype; rather, it's an evaluation of the genetic diversity generated around the current master haplotype.

Table 13: Mean substitutions per read vs master haplotype

ID	AvM
p0.p0	0.3278
p0.p3	0.3248
p100.p0	0.7103
p100.p3	0.8826
p200.p0	1.3773
p200.p3	1.0512

A more comprehensive evaluation of the explored genetic space can be provided by analyzing the set of read fractions corresponding to increasing differences relative to the current master haplotype. This approach offers a nuanced view of the quasispecies' genetic diversity, allowing for a more detailed understanding of its population structure. This method provides a more granular assessment of the genetic landscape, complementing the mean genetic distance approach.

Table 14: Fraction of reads at increasing substitutions vs master haplotype

ID	m00	m01	m02	m03	m04	m05	m06	Ov6
p0.p0	0.7338	0.2188	0.0370	0.0052	0.0011	0.0004	0.0002	0.0035
p0.p3	0.7367	0.2160	0.0369	0.0051	0.0010	0.0004	0.0003	0.0036
p100.p0	0.4943	0.3507	0.1221	0.0260	0.0042	0.0010	0.0004	0.0012
p100.p3	0.4326	0.3244	0.1918	0.0414	0.0068	0.0013	0.0004	0.0014
p200.p0	0.4379	0.2452	0.0878	0.0540	0.1157	0.0449	0.0100	0.0045
p200.p3	0.5026	0.2572	0.0891	0.0441	0.0707	0.0268	0.0059	0.0036

Note that, additionally, different subpopulations may coexist within the quasispecies, evolving around different subdominant haplotypes, and contributing to its overall genetic diversity.

In the table above, m00 is the fraction of reads corresponding to the current master, m01 the fraction of reads corresponding to all haplotypes showing a single difference relative to the master and so on. With Ov6 the fraction of reads corresponding to haplotypes with more than 6 differences relatives to the master.

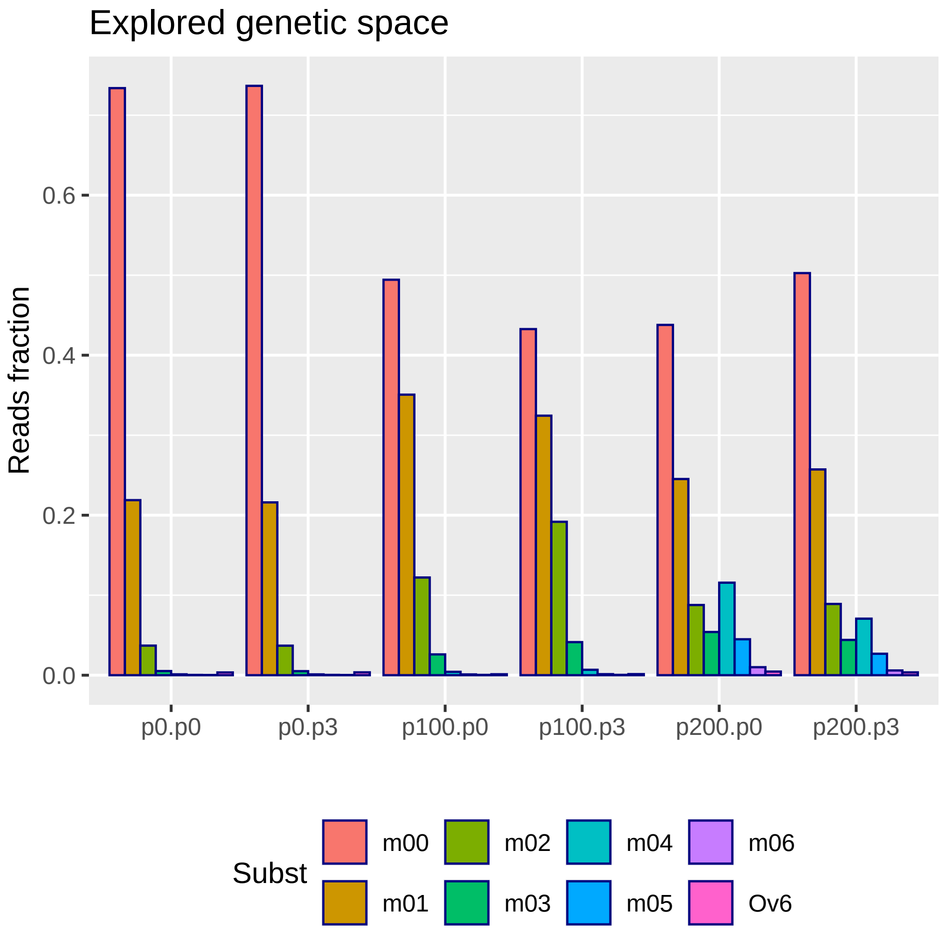

Figure 12: Fraction of reads at increasing substitutions, relative to the master haplotype.

Discussion

The evolution in quasispecies structure shows initially a prominent dominant haplotype with high values for Master and Top25, whereas the values of the fractions of reads accounting for low frequency haplotypes -Rare1, Rare2 and Singl- remain low. The indices of evenness -RLE1, RLE2, RLEinf, R5, R10 and R25- show low values as the quasispecies is dominated by a single, or very few haplotypes. As the quasispecies explores the genetic space, thanks to the low level fidelity of NS5B, new mutants are produced. Defective mutants and variants of low replication efficiency will be purged, while variants with replication efficiency will be promoted to higher frequencies according to their relative fitness in competition with existing genomes. In this respect as the quasispecies matures it will be adopting the structure of a flat-like quasispecies (Wilke et al. 2001; Wilke 2005; Lauring and Andino 2010), with a significant number of different genomes of similar fitness coexisting. Most will express the same phenotype -synonymous haplotypes to the master phenotype-, while others will express alternative functional phenotypes with different levels of efficiency (Gregori, Colomer-Castell, et al. 2024). Synonymous coding and other neutral mutations allow for a huge number of variants with the same or similar fitness. In the late evolution stages, a high number of haplotypes is expected, with a very smooth distribution of frequencies, and a high level of evenness.

The observed results, presented through graphics, tables, and statistical tests, corroborate the anticipated changes in the viral population as it transitions from p0 to p100, and subsequently from p100 to p200. These findings demonstrate an evolutionary trajectory from a more peaked fitness landscape towards a flatter, more diverse landscape. This pattern is consistent with the expected diversification of the viral quasispecies over time or under changing selective pressures. That is:

- Decrease in master frequency.
- Decrease in the fraction of top 25 haplotypes (Top25).
- Increase in general evenness values RLE1, RLE2 and RLEinf.
- Increase in top haplotypes evenness values R5, R10, and R25.
- Increase in the fraction of rare haplotypes represented by Rare1, Rare2 and Singl.
- Higher quasispecies synonymity.
- Larger portion of filled genetic space.

The changes are bigger in passing from p0 to p100, than in passing from p100 to p200. A reason may be a restriction in the available genetic space to explore after p100. As more space is explored the less degrees of freedom are left. On the other hand some indices, particularly those restricted in the range 0-1, may show an asymptotic behavior at increasing values.

Prolonged replication in this cell culture experiment meant 200 serial passages, which are equivalent to about 700 days of continuous HCV replication (García-Crespo et al. 2021). This is just a fraction of the time of chronic infections in patients with liver damage diagnosed, nevertheless we observed the same behavior, as with long chronic infections, although at a lower level.

References

Domingo, E., C. García-Crespo, and C. Perales. 2021. "Historical Perspective on the Discovery of the Quasispecies Concept." *Annu Rev Virol* 8 (1): 51–72. https://doi.org/10.1146/annurev-virology-091919-105900.

Domingo, E., C. García-Crespo, M. E. Soria, and C. Perales. 2023. "Viral Fitness, Population Complexity, Host Interactions, and Resistance to Antiviral Agents." In *Viral Fitness and Evolution*, 197–235. Current Topics in Microbiology and Immunology 439. Cham: Springer International Publishing. https://doi.org/10.1007/978-3-031-15640-3_6.

Domingo, E., M. E. Soria, I. Gallego, A. I. De Ávila, C. García-Crespo, B. Martínez-González, J. Gómez, and et al. 2020. "A New Implication of Quasispecies Dynamics: Broad Virus Diversification in Absence of External Perturbations." *Infection, Genetics and Evolution* 82: 104278. https://doi.org/10.1016/j.meegid.2020.104278.

Gallego, I., J. Gregori, M. E. Soria, C. García-Crespo, M. García-Álvarez, A. Gómez-González, R. Valiergue, et al. 2018. "Resistance of High Fitness Hepatitis c Virus to Lethal Mutagenesis." *Virology* 523: 100–109. https://doi.org/10.1016/j.virol.2018.07.030.

Gallego, I., J. Sheldon, E. Moreno, J. Gregori, J. Quer, J. I. Esteban, C. M. Rice, E. Domingo, and C. Perales. 2016. "Barrier-Independent, Fitness-Associated Differences in Sofosbuvir Efficacy Against Hepatitis c Virus." *Antimicrob Agents Chemother* 60 (6): 3786–93. https://doi.org/10.1128/AAC.00581-16.

Gallego, I., M. E. Soria, C. García-Crespo, Q. Chen, P. Martínez-Barragán, S. Khalfaoui, B. Martínez-González, et al. 2020. "Broad and Dynamic Diversification of Infectious Hepatitis c Virus in a Cell Culture Environment." *J Virol* 94 (6). https://doi.org/10.1128/JVI.01856-19.

García-Crespo, C., I. Gallego, M. E. Soria, A. I. de Ávila, B. Martínez-González, L. Vázquez-Sirvent, R. Lobo-Vega, et al. 2021. "Population Disequilibrium as Promoter of Adaptive Explorations in Hepatitis c Virus." *Viruses* 13 (4): 616. https://doi.org/10.3390/v13040616.

Gregori, J., S. Colomer-Castell, M. Ibañez-Lligoña, D. Garcia-Cehic, C. Campos, M. Buti, M. Riveiro-Barciela, et al. 2024. "In-Host Flat-Like Quasispecies: Characterization Methods and Clinical Implications." *Microorganisms* 12 (5): 1011. https://doi.org/10.3390/microorganisms12051011.

Gregori, J., M. Ibañez-Lligoña, S. Colomer-Castell, C. Campos, and J. Quer. 2024. "Virus Quasispecies Rarefaction: Subsampling with or Without Replacement?" *Viruses* 16 (5): 710. https://doi.org/10.3390/v16050710.

Gregori, J., M. Ibañez-Lligoña, S. Colomer-Castell, D. Garcia-Cehic, C. Campos, and J. Quer. 2024. "Association of Liver Damage and Quasispecies Maturity in Chronic HCV Patients: The Fate of a Quasispecies." *Microorganisms* submitted (to appear).

Gregori, J., M. E. Soria, I. Gallego, M. Guerrero-Murillo, J. I. Esteban, J. Quer, C. Perales, and E. Domingo. 2018. "Rare Haplotype Load as Marker for Lethal Mutagenesis." *PLoS One* 13 (10): e0204877. https://doi.org/10.1371/journal.pone.0204877.

Lauring, A. S., and R. Andino. 2010. "Quasispecies Theory and the Behavior of RNA Viruses." *PLoS Pathog.* 6 (7): e1001005. https://doi.org/10.1371/journal.ppat.1001005.

Moore, Jason H. 1999. "Bootstrapping, Permutation Testing and the Method of Surrogate Data." *Physics in Medicine & Biology* 44 (6): L11. https://doi.org/10.1088/0031-9155/44/6/101.

Moreno, E., I. Gallego, J. Gregori, A. Lucía-Sanz, M. E. Soria, V. Castro, N. M. Beach, et al. 2017. "Internal Disequilibria and Phenotypic Diversification During Replication of Hepatitis c Virus in a Noncoevolving Cellular Environment." *J Virol* 91 (10): e02505–16. https://doi.org/10.1128/JVI.02505-16.

R Core Team. 2024. *R: A Language and Environment for Statistical Computing*. Vienna, Austria: R Foundation for Statistical Computing. https://www.R-project.org/.

Sheldon, J., N. M. Beach, E. Moreno, I. Gallego, D. Piñeiro, E. Martínez-Salas, J. Gregori, et al. 2014. "Increased Replicative Fitness Can Lead to Decreased Drug Sensitivity of Hepatitis c Virus." *J Virol* 88 (20): 12098–111. https://doi.org/10.1128/JVI.01860-14.

Wilke, C. O. 2005. "Quasispecies Theory in the Context of Population Genetics." *BMC Evol Biol* 5 (44). https://doi.org/10.1186/1471-2148-5-44.

Wilke, C. O., J. L. Wang, C. Ofria, R. E. Lenski, and C. Adami. 2001. "Evolution of Digital Organisms at High Mutation Rates Leads to Survival of the Flattest." *Nature* 412 (6844). https://doi.org/10.1038/35085569.

Appendix

R code used for the permutation test on quasispecies diversity values.

The only required data is a vector of haplotype read counts for each quasispecies *rds1* and *rds2*.

```
###  Function computing selected diversity indices
diversity <- function(nr)
{
   p <- nr/sum(nr)
   q_0 <- length(p)              # Hill number for q=0
   q_1 <- exp(-sum(p*log(p)))    # Hill number for q=1
   q_2 <- 1/sum(p^2)             # Hill number for q=2
   q_inf <- 1/max(p)             # Hill number for q=Infinity
   RLE1 <- log10(q_1)/log10(q_0)      # Rel. log. evennes for q=1
   RLEinf <- log10(q_inf)/log10(q_0)  # Rel. log. evennes for q=Infinity

   p <- sort(p,decreasing=TRUE)  # Frequencies in decreasing order
   Master <- p[1]                # Master frequency
   Top25 <- sum(p[1:25])         # Top 25 haplotypes, reads fraction
   Rare2 <- sum(p[p<0.001])      # Fraction of reads for all Hpl <0.1%
   R10 <- p[10]/mean(p[1:10])    # Evenness in top 10 haplotypes

   vdiv <- c(Master,Top25,Rare2,R10,RLE1,RLEinf)
   names(vdiv) <- c('Master','Top25','Rare2','R10','RLE1','RLEinf')
   return(vdiv)
}

# Function to perform rarefaction
rarefy_counts <- function(counts, size) {
  raref <- sample( rep(seq_along(counts), counts),
                 size, replace = FALSE )
  tb <- tabulate(raref)
  return(tb[tb!=0]) # purge hpl with 0 counts
}

# One cycle of rarefaction
rarefy_sample <- function(counts,size)
{
   if(sum(counts) < size*1.05)
   {  return(counts)
   } else {  # Rarefy only if difference in size > 5%
     return(rarefy_counts(counts, size))
   }
}
```

```r
# Diversity with rarefaction, return median values.
observed_rarefied <- function(counts,size,n_raref=200)
{
  if(sum(counts) < size*1.05)
     return( diversity(counts) )

  rep.div <- replicate(n_raref,{ diversity(rarefy_sample(counts,size)) })
  apply(rep.div,1,median)
}

# Function to perform permutation test
permutation_test <- function(counts1, counts2, n_permutations = 1000)
{
  # Calculate observed diversity with rarefaction
  H1 <- observed_rarefied(counts1,min(sum(counts1),sum(counts2)))
  H2 <- observed_rarefied(counts2,min(sum(counts1),sum(counts2)))

  # Calculate observed difference in diversity indices with rarefaction
  observed_diff <- H2 - H1

  # Initialize a vector to store the permutation differences
  inms <- c('Master','Top25','Rare2','R10','RLE1','RLEinf')
  perm_diffs <- matrix(0,n_permutations,length(inms))
  colnames(perm_diffs) <- inms

  # Perform permutations
  for (i in 1:n_permutations) {

    # Rarefy the bigger sample
    raref_counts1 <- rarefy_sample(counts1,min(sum(counts1),sum(counts2)))
    raref_counts2 <- rarefy_sample(counts2,min(sum(counts1),sum(counts2)))

    # Combine the reads from both samples to a pool of reads
    combined_reads <- c(rep(seq_along(raref_counts1),raref_counts1),
                        rep(seq_along(raref_counts2),raref_counts2))
    n1 <- sum(raref_counts1)
    n2 <- sum(raref_counts2)

    # Randomly shuffle the pooled reads
    permuted_reads <- sample(combined_reads)

    # Split the permuted counts into two new groups
    perm_reads1 <- permuted_reads[1:n1]
    perm_counts1 <- tabulate(perm_reads1)
    perm_counts1 <- perm_counts1[perm_counts1!=0]
    perm_reads2 <- permuted_reads[(n1+1):(n1+n2)]
    perm_counts2 <- tabulate(perm_reads2)
```

```r
    perm_counts2 <- perm_counts2[perm_counts2!=0]

    # Calculate the diversity indices for the permuted groups
    perm_H1 <- diversity(perm_counts1)
    perm_H2 <- diversity(perm_counts2)

    # Calculate the difference in diversity indices for the permuted groups
    perm_diffs[i,] <- perm_H2 - perm_H1
  }

  # Return the test results
  list(
    H1=H1, H2=H2,
    observed_diff = observed_diff,
    perm_diffs = perm_diffs
  )
}

# Execute the permutations and collect results
ptest.res <- permutation_test(rds1, rds2, n_permutations = 1000)

# Calculate the p-value as the proportion of permuted differences
# that are greater than or equal to the observed difference
# p_value <- mean(abs(perm_diffs) >= abs(observed_diff))
p.vals <- sapply(1:ncol(ptest.res$perm_diffs), function(j)
              mean(abs(ptest.res$perm_diffs[,j]) >=
                   abs(ptest.res$observed_diff[j]) ))

### Data frame with all statistics
Perm.test.res <- data.frame(
           p0=ptest.res$H1,     # Quasispecies 1, observed diversity values
           p100=ptest.res$H2,   # Quasispecies , observed diversity values
           Obs.Diff=ptest.res$observed_diff,   # Observed differences
           # Median of the permuted differences
           Perm.Med=apply(ptest.res$perm_diffs,2,median),
           # Standardized Permutation Statistic
           Z=round(ptest.res$observed_diff/
                             apply(ptest.res$perm_diffs,2,sd),1),
           p.value=p.vals)
knitr::kable(Perm.test.res,digits=5)

### Plot histograms of permuted differences
perm.meds <- apply(ptest.res$perm_diffs,2,median)
lns <- data.frame(Ind=names(perm.meds),PermMed=perm.meds,
                  ObsDiff=ptest.res$observed_diff)
ptest.res$perm_diffs %>%
  data.frame() %>%
```

```r
  pivot_longer(everything(),names_to='Ind',values_to='Vals') %>%
  ggplot() +
  geom_histogram(aes(x=Vals),bins=30) +
  geom_vline(aes(xintercept=PermMed),lty=4,lwd=1,col='blue',data=lns) +
  labs(x='',title='Distribution of permuted differences') +
  facet_wrap(Ind~.) +
     theme(axis.text.x = element_text(angle = 60, vjust = 1, hjust=1))
```

Follows the session information:

```r
devtools::session_info()
```

```
## - Session info ---------------------------------------------------------------
##  setting  value
##  version  R version 4.3.3 (2024-02-29 ucrt)
##  os       Windows 11 x64 (build 22631)
##  system   x86_64, mingw32
##  ui       RTerm
##  language (EN)
##  collate  Catalan_Spain.utf8
##  ctype    Catalan_Spain.utf8
##  tz       Europe/Madrid
##  date     2024-10-08
##  pandoc   3.1.11 @ C:/Program Files/RStudio/resources/app/bin/quarto/bin/tools/ (via rmarkdo
##
## - Packages -------------------------------------------------------------------
##  package          * version  date (UTC) lib source
##  BiocGenerics     * 0.48.1   2023-11-01 [2] Bioconductor
##  Biostrings       * 2.70.3   2024-03-13 [2] Bioconductor 3.18 (R 4.3.3)
##  bitops             1.0-7    2021-04-24 [2] CRAN (R 4.3.1)
##  cachem             1.0.8    2023-05-01 [2] CRAN (R 4.3.3)
##  cli                3.6.2    2023-12-11 [2] CRAN (R 4.3.3)
##  colorspace         2.1-0    2023-01-23 [2] CRAN (R 4.3.3)
##  crayon             1.5.2    2022-09-29 [2] CRAN (R 4.3.3)
##  devtools           2.4.5    2022-10-11 [2] CRAN (R 4.3.3)
##  digest             0.6.35   2024-03-11 [2] CRAN (R 4.3.3)
##  dplyr            * 1.1.4    2023-11-17 [2] CRAN (R 4.3.3)
##  dqrng            * 0.3.2    2023-11-29 [2] CRAN (R 4.3.3)
##  ellipsis           0.3.2    2021-04-29 [2] CRAN (R 4.3.3)
##  evaluate           0.23     2023-11-01 [2] CRAN (R 4.3.3)
##  fansi              1.0.6    2023-12-08 [2] CRAN (R 4.3.3)
##  farver             2.1.1    2022-07-06 [2] CRAN (R 4.3.3)
##  fastmap            1.1.1    2023-02-24 [2] CRAN (R 4.3.3)
##  forcats          * 1.0.0    2023-01-29 [2] CRAN (R 4.3.3)
##  fs                 1.6.3    2023-07-20 [2] CRAN (R 4.3.3)
##  generics           0.1.3    2022-07-05 [2] CRAN (R 4.3.3)
##  GenomeInfoDb     * 1.38.8   2024-03-15 [2] Bioconductor 3.18 (R 4.3.3)
##  GenomeInfoDbData   1.2.11   2024-04-20 [2] Bioconductor
```

```
##  ggplot2         * 3.5.0     2024-02-23 [2] CRAN (R 4.3.3)
##  glue              1.7.0     2024-01-09 [2] CRAN (R 4.3.3)
##  gridExtra       * 2.3       2017-09-09 [2] CRAN (R 4.3.3)
##  gtable            0.3.4     2023-08-21 [2] CRAN (R 4.3.3)
##  highr             0.10      2022-12-22 [2] CRAN (R 4.3.3)
##  hms               1.1.3     2023-03-21 [2] CRAN (R 4.3.3)
##  htmltools         0.5.8.1   2024-04-04 [2] CRAN (R 4.3.3)
##  htmlwidgets       1.6.4     2023-12-06 [2] CRAN (R 4.3.3)
##  httpuv            1.6.15    2024-03-26 [2] CRAN (R 4.3.3)
##  IRanges         * 2.36.0    2023-10-24 [2] Bioconductor
##  knitr             1.46      2024-04-06 [2] CRAN (R 4.3.3)
##  labeling          0.4.3     2023-08-29 [2] CRAN (R 4.3.1)
##  later             1.3.2     2023-12-06 [2] CRAN (R 4.3.3)
##  lifecycle         1.0.4     2023-11-07 [2] CRAN (R 4.3.3)
##  lubridate       * 1.9.3     2023-09-27 [2] CRAN (R 4.3.3)
##  magrittr          2.0.3     2022-03-30 [2] CRAN (R 4.3.3)
##  memoise           2.0.1     2021-11-26 [2] CRAN (R 4.3.3)
##  mime              0.12      2021-09-28 [2] CRAN (R 4.3.1)
##  miniUI            0.1.1.1   2018-05-18 [2] CRAN (R 4.3.3)
##  munsell           0.5.1     2024-04-01 [2] CRAN (R 4.3.3)
##  pillar            1.9.0     2023-03-22 [2] CRAN (R 4.3.3)
##  pkgbuild          1.4.4     2024-03-17 [2] CRAN (R 4.3.3)
##  pkgconfig         2.0.3     2019-09-22 [2] CRAN (R 4.3.3)
##  pkgload           1.3.4     2024-01-16 [2] CRAN (R 4.3.3)
##  profvis           0.3.8     2023-05-02 [2] CRAN (R 4.3.3)
##  promises          1.3.0     2024-04-05 [2] CRAN (R 4.3.3)
##  purrr           * 1.0.2     2023-08-10 [2] CRAN (R 4.3.3)
##  R6                2.5.1     2021-08-19 [2] CRAN (R 4.3.3)
##  Rcpp              1.0.12    2024-01-09 [2] CRAN (R 4.3.3)
##  RCurl             1.98-1.14 2024-01-09 [2] CRAN (R 4.3.2)
##  readr           * 2.1.5     2024-01-10 [2] CRAN (R 4.3.3)
##  remotes           2.5.0     2024-03-17 [2] CRAN (R 4.3.3)
##  rlang             1.1.3     2024-01-10 [2] CRAN (R 4.3.3)
##  rmarkdown         2.26      2024-03-05 [2] CRAN (R 4.3.3)
##  rstudioapi        0.16.0    2024-03-24 [2] CRAN (R 4.3.3)
##  S4Vectors       * 0.40.2    2023-11-23 [2] Bioconductor
##  scales            1.3.0     2023-11-28 [2] CRAN (R 4.3.3)
##  sessioninfo       1.2.2     2021-12-06 [2] CRAN (R 4.3.3)
##  shiny             1.8.1.1   2024-04-02 [2] CRAN (R 4.3.3)
##  stringi           1.8.3     2023-12-11 [2] CRAN (R 4.3.2)
##  stringr         * 1.5.1     2023-11-14 [2] CRAN (R 4.3.3)
##  tibble          * 3.2.1     2023-03-20 [2] CRAN (R 4.3.3)
##  tidyr           * 1.3.1     2024-01-24 [2] CRAN (R 4.3.3)
##  tidyselect        1.2.1     2024-03-11 [2] CRAN (R 4.3.3)
##  tidyverse       * 2.0.0     2023-02-22 [2] CRAN (R 4.3.3)
##  timechange        0.3.0     2024-01-18 [2] CRAN (R 4.3.3)
##  tzdb              0.4.0     2023-05-12 [2] CRAN (R 4.3.3)
##  urlchecker        1.0.1     2021-11-30 [2] CRAN (R 4.3.3)
```

```
##  usethis            2.2.3     2024-02-19 [2] CRAN (R 4.3.3)
##  utf8               1.2.4     2023-10-22 [2] CRAN (R 4.3.3)
##  vctrs              0.6.5     2023-12-01 [2] CRAN (R 4.3.3)
##  withr              3.0.0     2024-01-16 [2] CRAN (R 4.3.3)
##  xfun               0.43      2024-03-25 [2] CRAN (R 4.3.3)
##  xtable             1.8-4     2019-04-21 [2] CRAN (R 4.3.3)
##  XVector          * 0.42.0    2023-10-24 [2] Bioconductor
##  yaml               2.3.8     2023-12-11 [2] CRAN (R 4.3.2)
##  zlibbioc           1.48.2    2024-03-13 [2] Bioconductor 3.18 (R 4.3.3)
##
##  [1] C:/Users/Admin/AppData/Local/R/win-library/4.3
##  [2] C:/Program Files/R/R-4.3.3/library
##
## ------------------------------------------------------------------------------
```
