# Supplementary material for: Association of Liver Damage and Quasispecies Maturity in Chronic HCV Patients: The Fate of a Quasispecies
Source: Microorganisms. 2024 Oct 31;12(11):2213. doi: 10.3390/microorganisms12112213 (PMC11596025; doi:10.3390/microorganisms12112213)
Supplement: Supplementary file 1 [file microorganisms-12-02213-s001.zip › Supplementary material full cohort study with QS maturity indicators.pdf]

Association of liver damage and quasispecies maturity in chronic HCV patients: The fate of a quasispecies

Suppl. Material: Full Cohort Classification by Quasispecies Maturity Indicators

Josep Gregori*, Marta Ibañez-Lligoña, Sergi Colomer-Castell, Carolina Campos, Damir García-Cehic, and Josep Quer†

2024-09-26

Introduction

This is a supplementary material to the article "Association of liver damage and quasispecies maturity in chronic HCV patients: The fate of a quasispecies" (Gregori, Ibañez-Lligoña, et al. 2024) were we extend the results observed on patients with fibrosis data, to the full cohort of chronically infected HCV patients who failed previous treatments, and with no liver damage data available. Our objective in this supplementary material is to compare the quasispecies data of patients in the cohort without liver damage data to those with fibrosis scores. In the main text we established an association between liver damage progression and quasispecies maturity. See the main text for further details.

Methods

Assigning patients to groups

From the full set of 75 patients, with quasispecies rarefied to 20,000 reads per amplicon and strand, we excluded patients with available fibrosis data. The remaining 56 patients were ranked by their median RLEinf value per patient, and distributed in three groups. The 19 top-ranked patients were labeled 'Top', representing those with flatter quasispecies profiles in the cohort. The 18 bottom-ranked patients were labeled 'Reg', indicating those with more regular quasispecies profiles typical of acute infections with a highly prevalent master genome. The remaining 19 patients were labeled 'Mid'. This categorization resulted in 19 Top, 19 Mid, and 18 Reg patients, none of whom had liver damage data. Among the 19 patients with fibrosis scores in the cohort, the two patients with F1 scores were excluded from this study. RLEinf is defined as the ratio $\log_{10}(1/\text{Master})/\log_{10}(H)$, where H represents the number of haplotypes. A high RLEinf value implies a highly diverse quasispecies with no dominant haplotype (flat-like quasispecies). Conversely, a low RLEinf value indicates a quasispecies with limited diversity and a highly dominant master haplotype (regular quasispecies).

In summary, our dataset consists in 17 patients with fibrosis scores, and 56 with no liver damage data, labeled Reg (18), Mid (19) and Top (19) according to the rank of the median RLEinf per patient.

*josep.gregori@gmail.com
†josep.quer@vhir.org

Table 1: Number of patients per group

Group	N
F2	3
F3	5
F4+	9
Mid	19
Reg	18
Top	19

This translates to the following number of amplicons per group:

Table 2: Number of amplicons per group

Group	NS3	NS5A	NS5B1	NS5B2
F2	3	3	3	2
F3	4	5	5	2
F4+	8	9	9	8
Reg	17	16	15	12
Mid	19	19	18	18
Top	17	19	16	16

Indicators of quasispecies maturity

Several quasispecies maturity indicators were selected, based on a previous study (Gregori, Colomer-Castell, et al. 2024). The next table shows the expected level of each indicator in the two limiting cases, flat vs regular quasispecies:

Indicator	Description	Regular Qs	Flat Qs
Master	Dominant haplotype frequency	High	Low
Rare	Fraction of reads for hpl $\leq 1\%$	Low	High
Top25	Fraction of reads for top 25 hpl.	High	Low
Top25R	Ratio Top25 to master reads	Low	High
Singl	Fraction of singletons	Low	High
RLE_1	Relative logarithmic evennes at $q = 1$	Low	High
RLE_2	Relative logarithmic evennes at $q = 2$	Low	High
RLE_∞	Relative logarithmic evennes at $q = \infty$	Low	High
Rk	Evenness on top k haplotypes (k=10, 25)	Low	High

With

$$RLEq = RLE(p, q) = \log_{10}(D(p, q))/\log_{10}(D(p, 0))$$

and $D(q, p)$ the Hill number of order q.

$$D(q, p) = \left(\sum_{i=1}^{H} p_i^q\right)^{1/(1-q)}$$

H number of haplotypes, $p = (p_1, p_2, \ldots, p_H)$ the set of haplotype frequencies.

On the set of haplotype frequencies sorted in decreasing order, $p_1 \geq p_2 \geq \cdots \geq p_H$, the following indicators are computed:

$$\text{Top25}(p) = \left(\sum_{i=1}^{m} p_i \;\middle|\; m = \min(25, H) \right)$$

$$\text{Top25R}(p) = \frac{\text{Top25}(p)}{p_1}$$

and an indicator of evenness on the set of k top haplotypes:

$$R_k(p) = \left(\frac{l \cdot p_l}{\sum_{i=1}^{l} p_i} \;\middle|\; l = \min(k, H) \right)$$

Results: Full cohort quasispecies characteristics

Maturity scores distribution

Amplicon-based quasispecies maturity indicators distribution for the full cohort.

Table 4: Distribution of indicator values in the full cohort

Ind	Min	Q1	Q2	Q3	Max
Top25	0.0061	0.1468	0.3035	0.4328	0.6388
Master	0.0009	0.0464	0.1383	0.3105	0.5706
Rare	0.4289	0.6259	0.7677	0.9187	1.0000
Singl	0.1129	0.2713	0.4127	0.6276	0.9840
R5	0.0228	0.0912	0.1859	0.3364	0.8466
R10	0.0342	0.0724	0.1698	0.3087	0.7693
R25	0.0534	0.0982	0.1603	0.2706	0.6124
RLE1	0.4593	0.6710	0.8031	0.9121	0.9992
RLE2	0.1410	0.2637	0.4068	0.5838	0.9961
RLEinf	0.0706	0.1334	0.2178	0.3222	0.7090

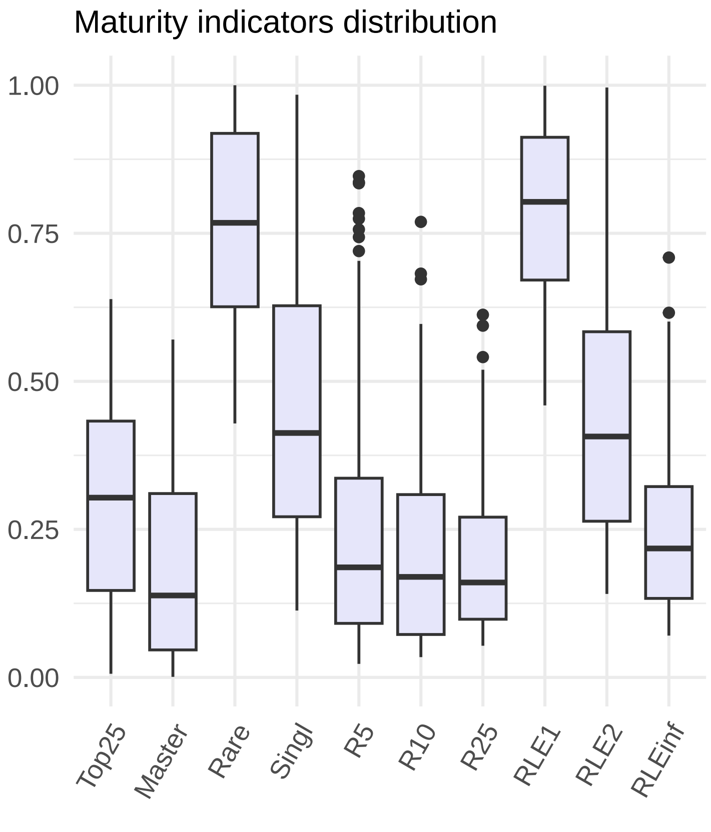

Figure 1: Full cohort maturity indicators distribution.

Amplicon-based quasispecies maturity indicators distribution by group

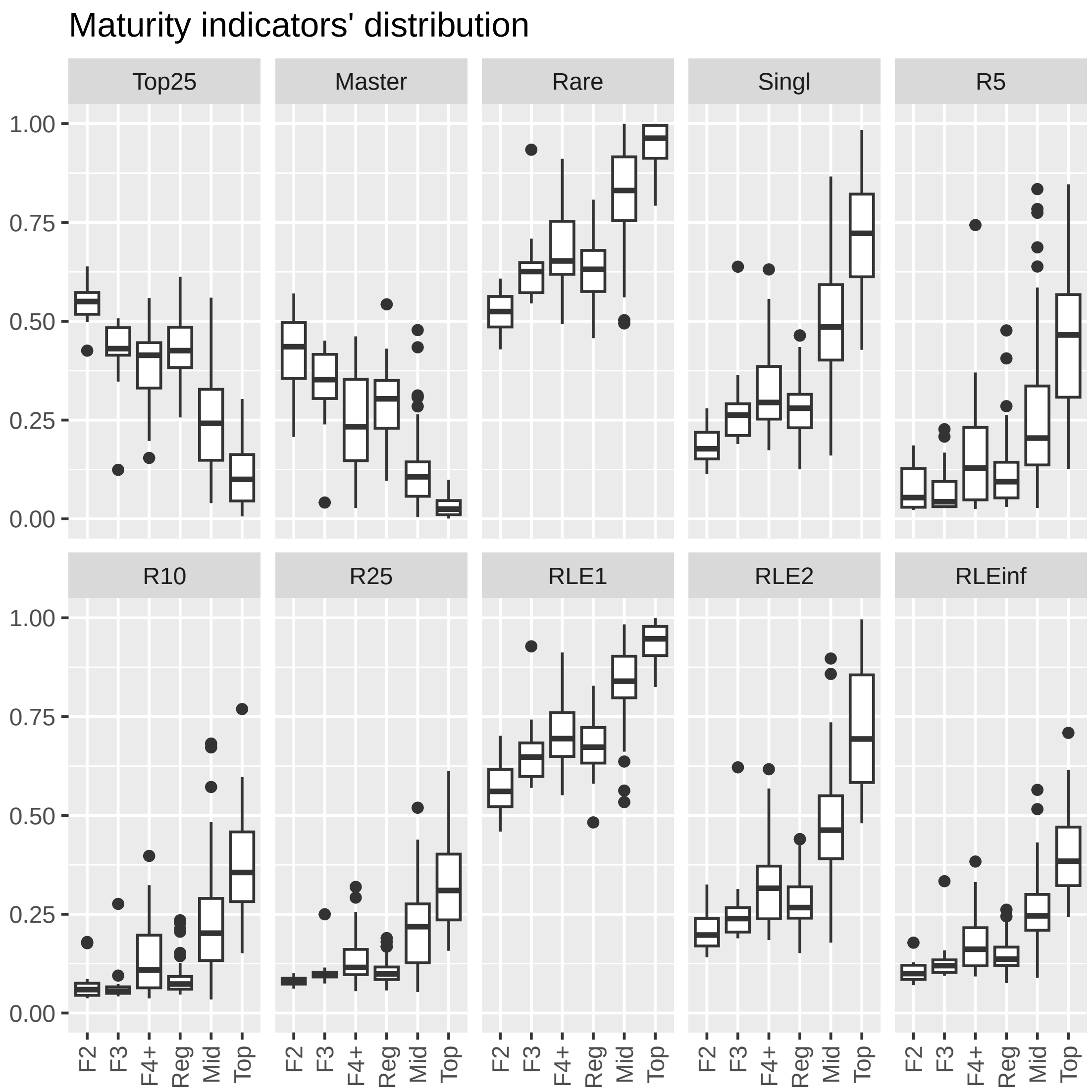

Figure 2: Full cohort maturity indicators' distribution by group.

Median values

Median indicator values per amplicon and group

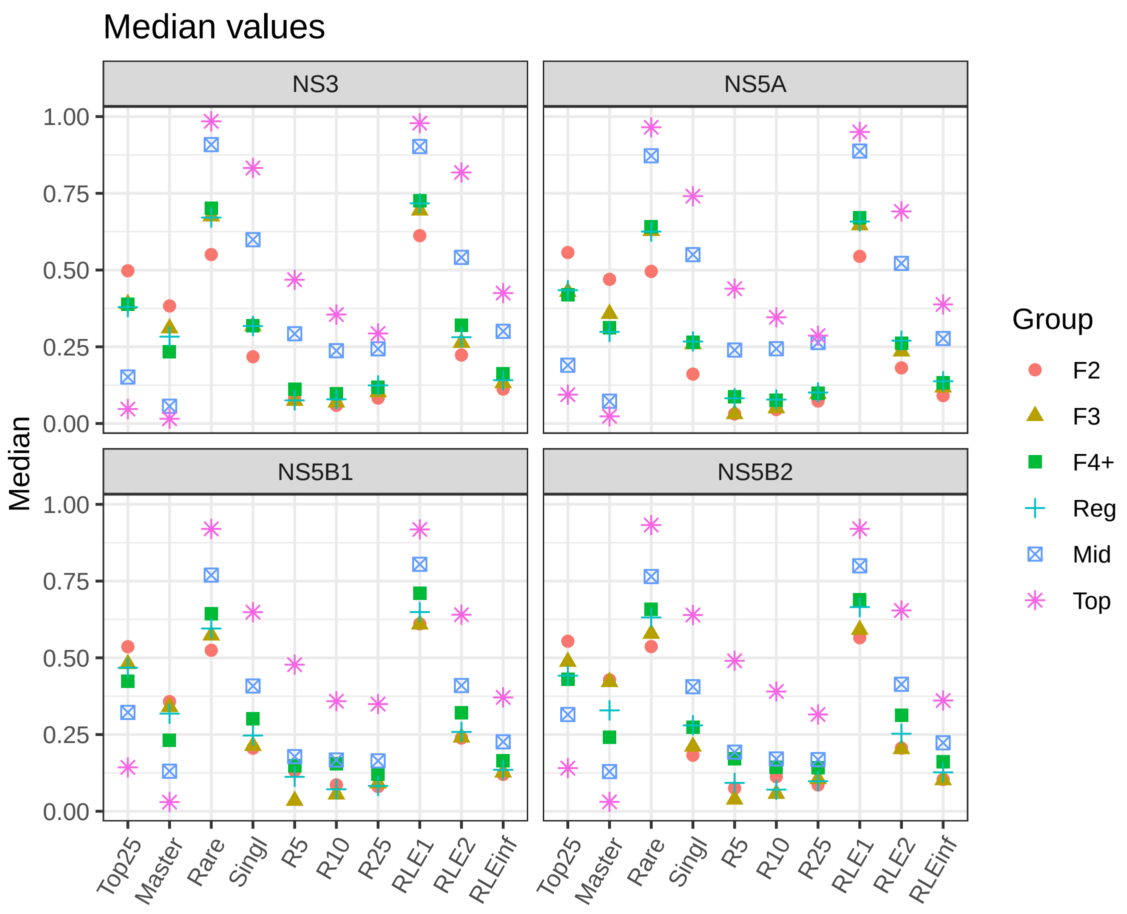

Figure 3: Median indicator values per amplicon and group.

Table 5: Median indicator values per amplicon and group

Ampl	Group	Master	Top25	Rare	Singl	RLE1	RLE2	RLEinf	R5	R10	R25
NS3	F2	0.3830	0.4980	0.551	0.218	0.612	0.223	0.1120	0.0826	0.0593	0.0829
NS3	F3	0.3100	0.3900	0.675	0.318	0.694	0.263	0.1320	0.0741	0.0689	0.1020
NS3	F4+	0.2330	0.3890	0.702	0.318	0.725	0.320	0.1610	0.1120	0.0971	0.1180
NS3	Reg	0.2830	0.3790	0.671	0.318	0.718	0.281	0.1410	0.0754	0.0789	0.1240
NS3	Mid	0.0563	0.1510	0.909	0.599	0.903	0.541	0.3000	0.2920	0.2370	0.2430
NS3	Top	0.0154	0.0471	0.985	0.832	0.979	0.818	0.4250	0.4680	0.3550	0.2930
NS5A	F2	0.4700	0.5570	0.496	0.161	0.545	0.181	0.0908	0.0312	0.0455	0.0735
NS5A	F3	0.3570	0.4290	0.628	0.259	0.646	0.235	0.1180	0.0310	0.0501	0.0964
NS5A	F4+	0.3120	0.4190	0.641	0.265	0.671	0.262	0.1330	0.0865	0.0758	0.0983
NS5A	Reg	0.2990	0.4340	0.625	0.267	0.658	0.270	0.1380	0.0823	0.0780	0.1010
NS5A	Mid	0.0726	0.1900	0.872	0.550	0.888	0.522	0.2770	0.2390	0.2440	0.2640
NS5A	Top	0.0236	0.0940	0.965	0.741	0.950	0.691	0.3880	0.4390	0.3460	0.2860
NS5B1	F2	0.3570	0.5360	0.525	0.205	0.611	0.238	0.1200	0.1320	0.0860	0.0804
NS5B1	F3	0.3400	0.4800	0.573	0.214	0.609	0.241	0.1270	0.0345	0.0550	0.0910
NS5B1	F4+	0.2320	0.4240	0.644	0.301	0.711	0.320	0.1650	0.1490	0.1540	0.1200
NS5B1	Reg	0.3180	0.4680	0.595	0.247	0.649	0.259	0.1350	0.1120	0.0717	0.0827
NS5B1	Mid	0.1310	0.3220	0.769	0.408	0.805	0.410	0.2260	0.1780	0.1670	0.1640
NS5B1	Top	0.0300	0.1430	0.920	0.649	0.918	0.640	0.3710	0.4780	0.3590	0.3490
NS5B2	F2	0.4280	0.5540	0.537	0.183	0.565	0.206	0.1040	0.0750	0.1130	0.0858
NS5B2	F3	0.4210	0.4870	0.579	0.211	0.592	0.203	0.1020	0.0385	0.0572	0.1050
NS5B2	F4+	0.2410	0.4300	0.658	0.274	0.689	0.313	0.1610	0.1710	0.1430	0.1420
NS5B2	Reg	0.3290	0.4420	0.632	0.280	0.665	0.253	0.1270	0.0925	0.0704	0.0979
NS5B2	Mid	0.1290	0.3150	0.765	0.406	0.800	0.414	0.2230	0.1920	0.1700	0.1680
NS5B2	Top	0.0306	0.1410	0.933	0.640	0.920	0.654	0.3610	0.4900	0.3900	0.3150

Correlation between indicators

The selected indicators measure specific characteristics of quasispecies structure, with two limiting cases. The first corresponds to the case of a quasispecies with a single genome, the second to a highly diverse quasispecies with a high number of haplotypes, all at similar frequencies. The first may be exemplified by the first stage of an acute infection caused by the transmission of a single virus. The second with a highly evolved quasispecies with multiple genomes expressing the same phenotype and alternative highly functional phenotypes, a flat-like quasispecies. The first has Master and Top25 at a value near 1, whereas the others near 0. The second has Master and Top 25 near 0, whereas the others near 1. These indicators despite measuring different well defined quasispecies characteristics are highly correlated.

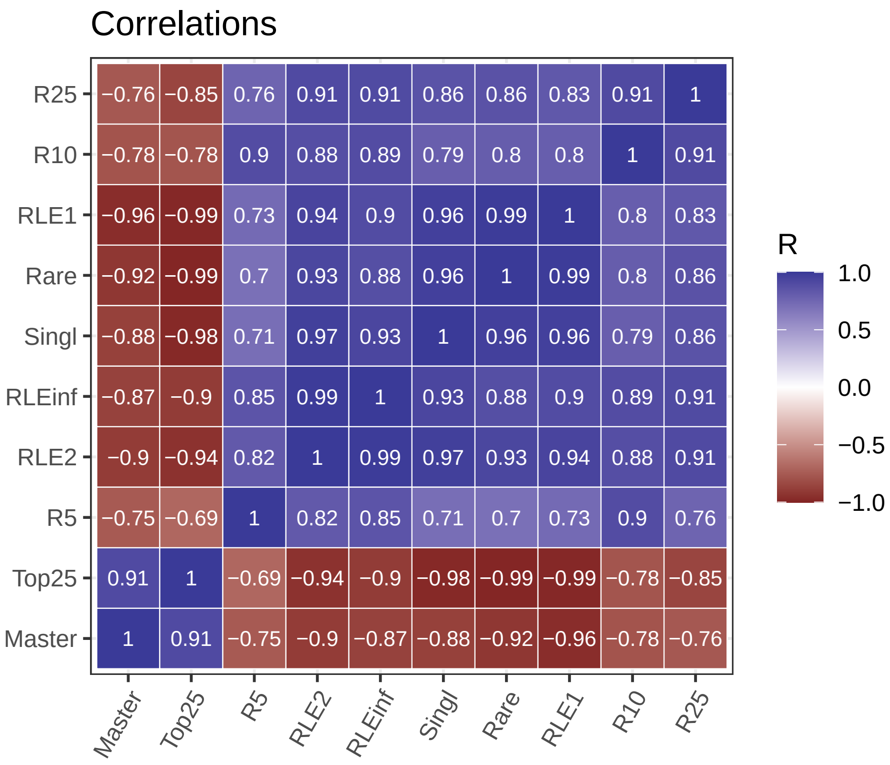

Figure 4: Maturity indicators correlation.

Principal Components Analysis

Principal Component Analysis (PCA) plays a significant role in observing unsupervised and non-parametric clustering of samples according to their labels. PCA reduces the dimensionality of the dataset while preserving as much variance as possible. PCA does not assume any specific distribution for the data, making it compatible with non-parametric clustering methods.

With our data the two main Principal Components, PC1 and PC2, account for 94.7% of the full variability, making the representation on the PC1/PC2 plane highly informative. This is caused by the high correlation between indicators. In the plot, each dot corresponds to the strand mean value of a single amplicon, with two to four amplicons per patient.

Table 6: Principal components importance

	PC1	PC2	PC3	PC4
Standard deviation	2.9765	0.7815	0.5124	0.3622
Proportion of Variance	0.8860	0.0611	0.0263	0.0131
Cumulative Proportion	0.8860	0.9470	0.9733	0.9864

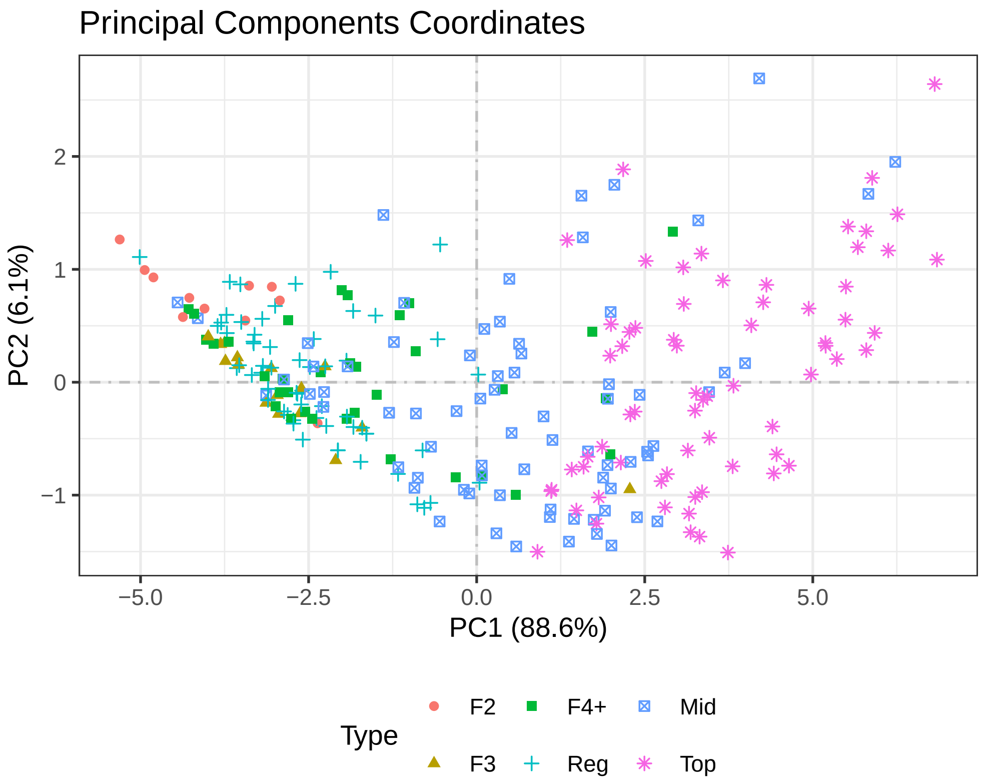

Figure 5: Scatterplot on principal components coordinates.

F2, F3 and several Reg samples cluster tightly along a line in the PC1/PC2 plane, indicating a common evolutionary path in the early stages. Conversely, F4+, Mid and mainly Top samples are more scattered, reflecting greater evolutionary heterogeneity.

The component coefficients of PC1 align with the expected evolutionary trend. Higher values of Master and Top25 indicators are characteristic of regular quasispecies profiles. These metrics typically represent viral populations with a highly dominant haplotype, or very few dominant haplotypes. Conversely, higher values of the other indicators are associated with higher diversity, evidenced by an increasing fraction of the rare haplotypes load. And finally, a flat-like quasispecies profile characterized by high evenness values, with low top haplotype frequencies.

According to PC1 component coefficients, samples placed to positive PC1 coordinates (PC1 scores) represent flatter quasispecies, whereas samples placed to negative PC1 coordinates represent more regular quasispecies. The component coefficients of PC2 show a vector of variation orthogonal to PC1, with indicators typical of the rare haplotypes fraction -Singl, Rare and RLE1- that oppose to top haplotypes evenness indicators represented by R5, R10, R25 and RLEinf.

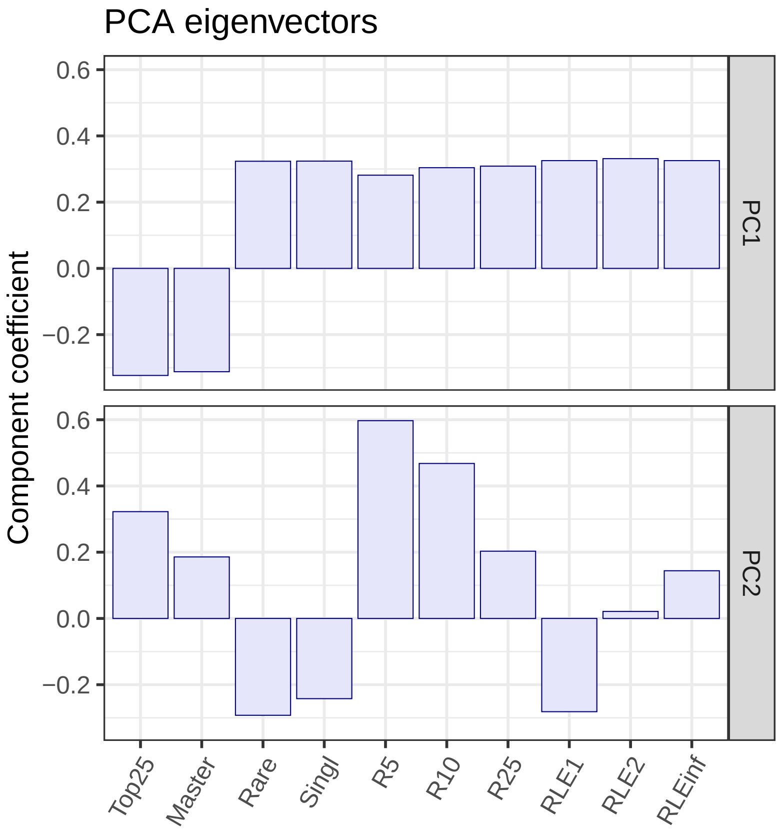

Figure 6: Principal components, coefficients of the two main eigenvectors.

Notably the absolute value of the contribution of each indicator -centered and scaled- to PC1 is almost equivalent.

The next table and plot show the median value of each indicator in each quadrant of the PC1/PC2 plane, including the center of coordinates, to ease the interpretation of the scatterplot.

Table 7: Median values in each PC1/PC2 plane quadrant

Q	N	Master	Top25	Rare	Singl	RLE1	RLE2	RLEinf	R5	R10	R25
(-,+)	75	0.350	0.484	0.579	0.229	0.631	0.241	0.122	0.111	0.068	0.090
(-,-)	60	0.263	0.392	0.673	0.306	0.712	0.292	0.151	0.083	0.074	0.109
(0,0)	0	0.178	0.296	0.767	0.458	0.788	0.451	0.246	0.246	0.208	0.197
(+,-)	74	0.056	0.144	0.917	0.637	0.914	0.569	0.302	0.263	0.254	0.255
(+,+)	54	0.027	0.147	0.928	0.633	0.921	0.653	0.381	0.543	0.444	0.351

The quasispecies evolution course is represented as Q2 (-,+) → Q3 (-,-) → Q4 (+,-) → Q1 (+,+)

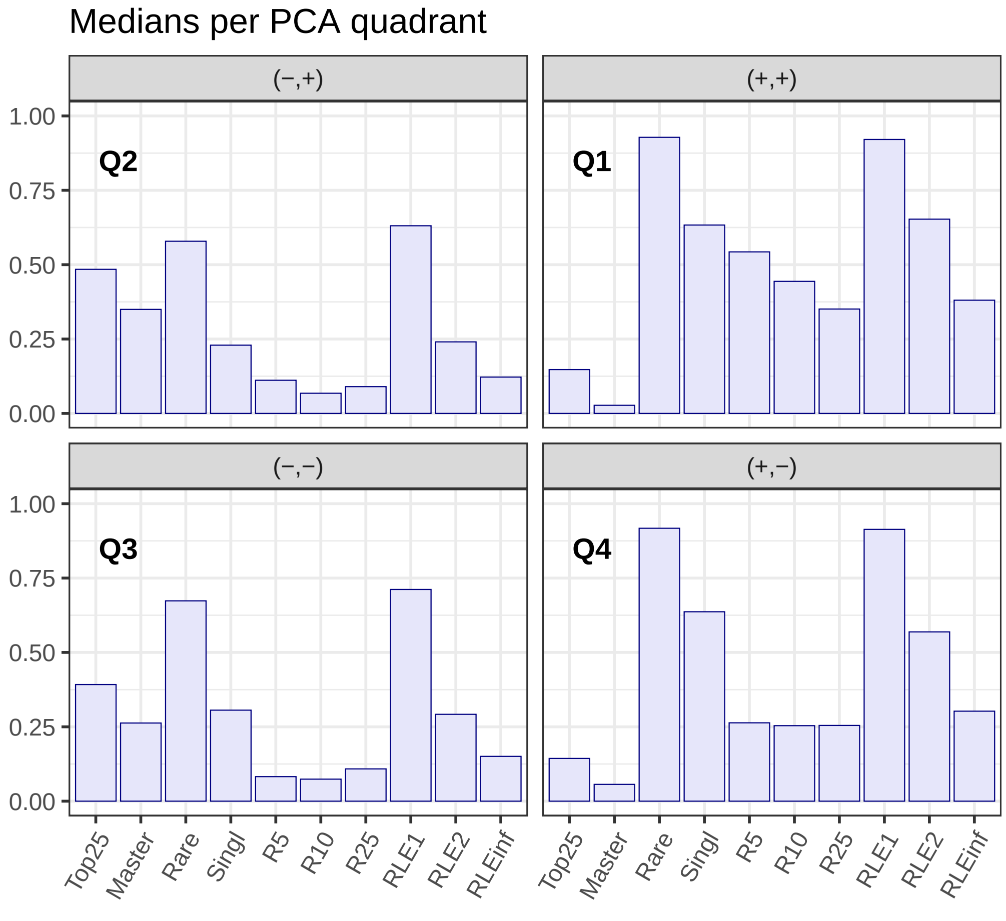

Figure 7: Median of maturity indicators per PC1/PC2 plane quadrant.

With Q2 (-,+) the most akin to a regular quasispecies, and Q1 (+,+) the most akin to a flat-like quasispecies.

Table 8: Quadrant transitions. Relative diferences (%) of quadrant medians

Trans	Master	Top25	Rare	Singl	RLE1	RLE2	RLEinf	R5	R10	R25
Q3-Q2	-24.9	-19.1	16.4	33.4	12.8	21.4	23.4	-25.9	9.3	20.6
Q4-Q3	-78.5	-63.3	36.2	108.2	28.4	94.9	100.8	219.3	242.8	134.5
Q1-Q4	-51.7	2.6	1.2	-0.6	0.8	14.7	25.8	106.1	74.9	37.8

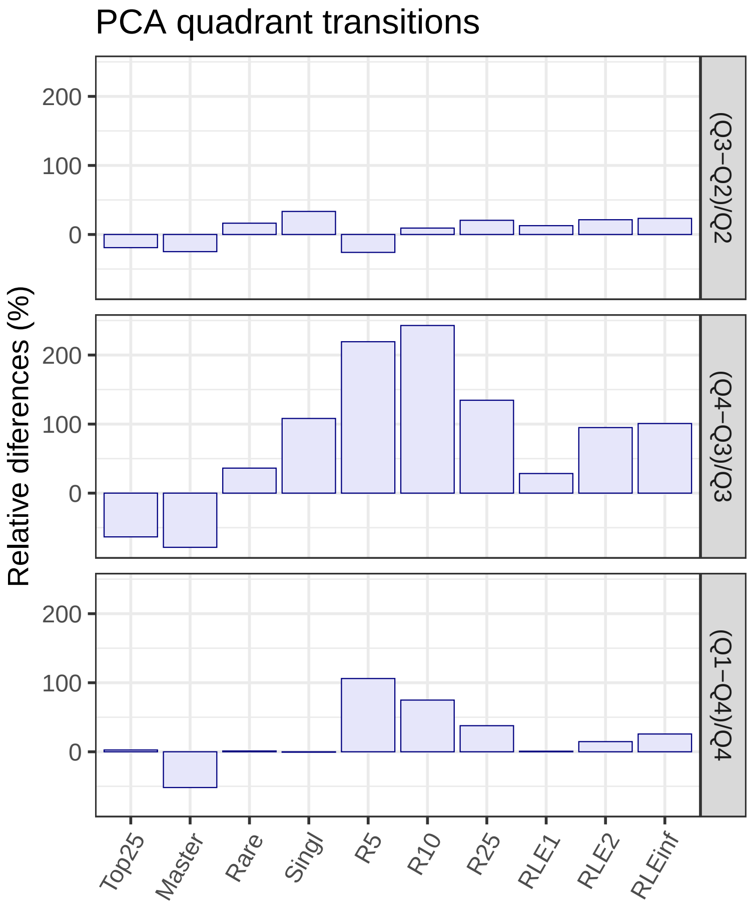

Figure 8: Relative differences in median values of maturity indicators, in the trasition between PC1/PC2 quandrants.

The transition Q2 to Q3 is characterized by an increase in the fractions of rare halotypes -Rare, Singl, RLE1- balanced by the decrease in top haplotypes fractions -Master and Top25-. The transition Q3 to Q4 shows the higher differences, and is characterized by a further decrease in Master and Top25, with an increase in all other indicators, mainly singletons and the top haplotypes evenness indices, including RLE2 and RLEinf. Finally, the transition Q4 to Q1 is characterized by an increase in the evenness indices, mainly of the top haplotypes -R5, R10, and R25- a further decrease in the master haplotype frequency, and very limited variations in the other indicators.

Table 9: Dispersion per quadrant. Mean PCA coordinates and mean distance to the PCA origin per quadrant

Q	mean.PC1	mean.PC2	mean.d
(-,+)	-2.977	0.478	3.047
(-,-)	-1.954	-0.425	2.084
(+,-)	2.061	-0.766	2.305
(+,+)	3.482	0.859	3.656

Ranks

Ranking samples by quasispecies maturity indicators allows for a standardized and objective assessment of patients and provides insights into the evolutionary dynamics of viruses. Our aim here is to locate the patients with no liver damage data among those having fibrosis scores. With this purpose, amplicons are ranked based on quasispecies maturity indicators and assigned percentiles according to these ranks. The median of the patient amplicons's percentile is the percentile taken for each patient. This method yields a median percentile rank for each patient, enabling the sorting of patients based on their quasispecies maturity indicators. The resulting non-parametric classification encompasses all patients, regardless of whether they have liver damage data available.

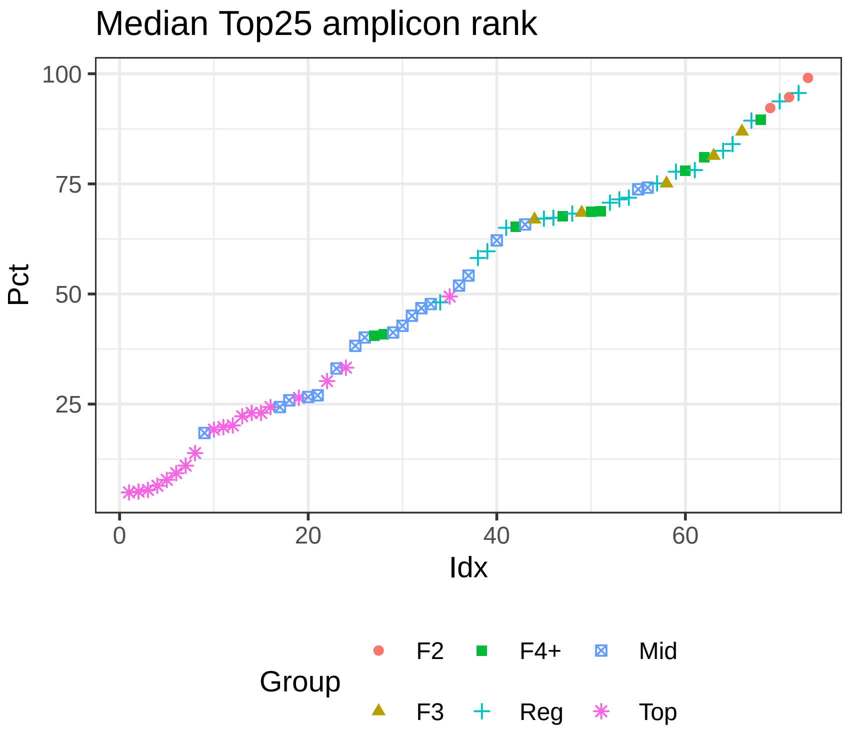

Figure 9: Patients ranked by median Top25 amplicon rank.

As another possibility the rank percentiles of all indicators may be averaged for each patient, this corresponds to giving equal weight to each indicator. Master and Top25 are multiplied previously by -1,

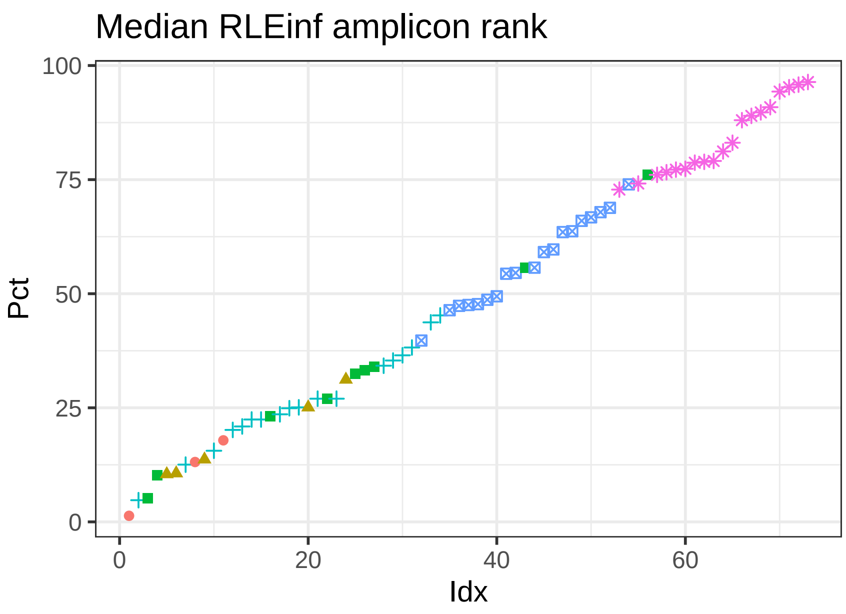

Figure 10: Patients ranked by median RLEinf amplicon rank.

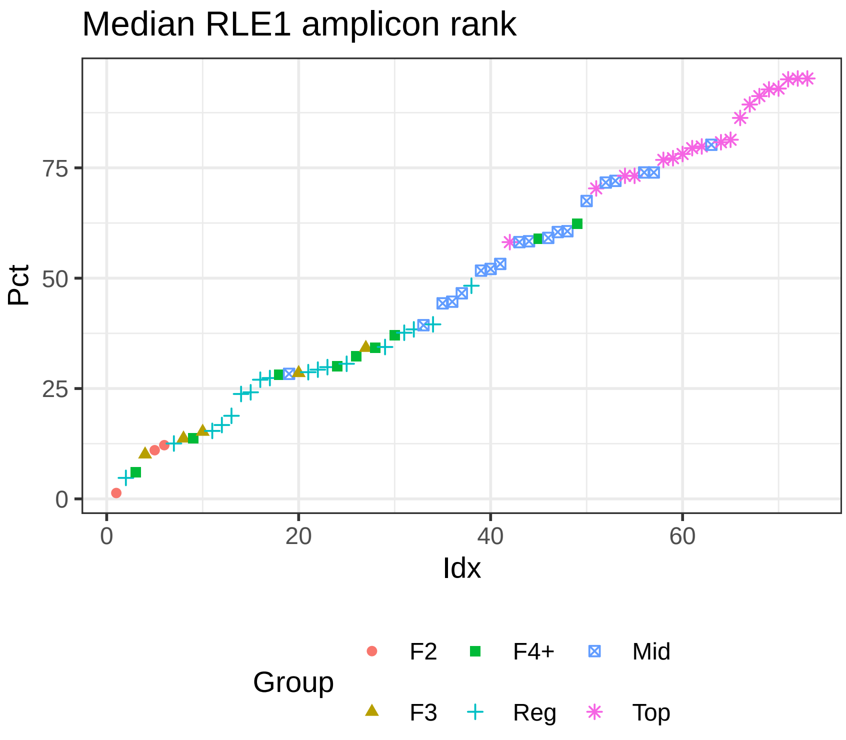

Figure 11: Patients ranked by median RLE1 amplicon rank.

so that all indicators show the same trend towards higher values as the quasispecies diversify.

The mean percentile rank per patient across all indicators and amplicons is computed as follows:

$$R_i = \frac{100}{M \cdot N \cdot A} \cdot \sum_{k=1}^{A} \sum_{j=1}^{M} \text{rank}_j(X_{ikj})$$

with patients $i = 1, \ldots, N$, amplicons $k = 1, \cdots, A$, indicators $j = 1, \ldots, M$, X_{ikj} the value of indicator j for patient i and amplicon k, and $\text{rank}_j(X_{ikj})$ the rank of X_{ikj} among all X_{ik} values for indicator j, that is the full set of values for indicator j.

As an average we obtain a smoothed sorting where variations within individual indicators are compensated. It integrates information from multiple quasispecies maturity indicators, providing a more holistic view of viral population dynamics, and helps mitigate the impact of outliers or extreme values in individual indicators, resulting in a more robust overall measure.

Similarly, the first principal component (PC1) accounts for 88.6% of our dataset's total variation, allowing patient ranking based on PC1 coordinates. These coordinates, a linear combination of all indicators, capture maximum variation by projecting the dataset onto the PC1 axis. This method resembles a weighted average, with indicators weighted by their PC1 coefficients. We multiply PC1 by -1 before ranking to align with our expected diversification profile. This approach summarizes complex, multidimensional data into a single score, encapsulating most of the original variation while considering interrelationships among all indicators.

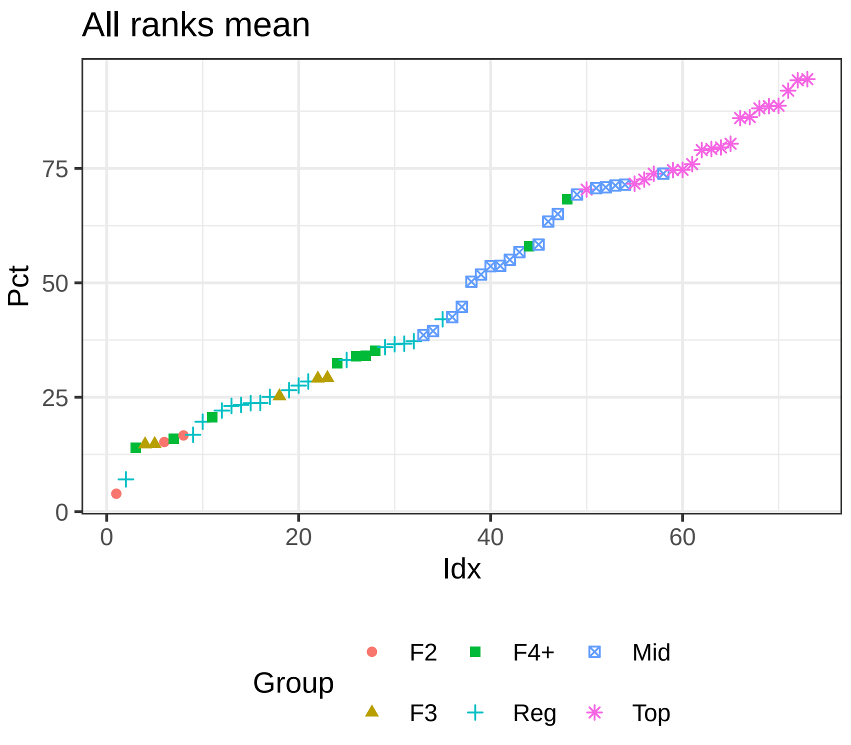

Figure 12: Patients ranked by all ranks mean.

The full averaged sorting by rank percentiles, or the ranks by PC1, show very similar results to those observed individually with each indicator, with Mid and Top above most F4+, and Reg merged between F2 to F4 samples.

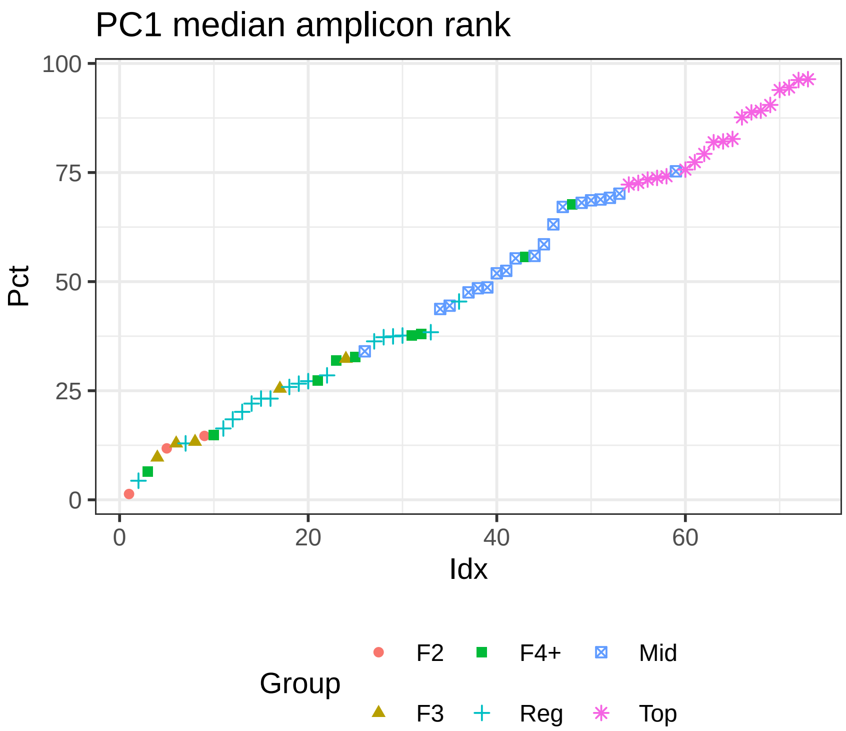

Figure 13: Patients ranked by PC1 median amplicon rank.

A summary quasispecies maturity score

As seen above, the PC1 coefficients of all indicators are all approximately equal, in absolute value, this suggests a linear combination of all indicators producing a summary maturity score. Let's take all coefficients in absolute value equal to $\sqrt{1/10}$ to preserve vector normality, with negative value for Master and Top25, and positive value for all other indicators, and divide each coefficient by the observed standard deviation of each indicator in the full cohort, to scale the values. The obtained coefficients are finally divided by the maximum of them, to have all coefficients in the range 0-1 in absolute value.

Table 10: Coefficients for a summary maturity score

	Coef
Master	-0.8268
Top25	-0.7349
Rare	0.7500
Singl	0.5510
RLE1	0.8799
RLE2	0.5719
RLEinf	0.9318
R5	0.6102
R10	0.7698
R25	1.0000

The patients in the cohort may be scored by the mean of the summary score values of its amplicons.

The distribution of scores by patient group corresponds to:

Table 11: Distribution of patient's maturity scores by group

Group	Min	Q1	Q2	Q3	Max
F2	0.3231	0.5784	0.8337	0.8522	0.8706
F3	0.8350	0.8645	1.1390	1.2539	1.3550
F4+	0.8210	0.9798	1.3867	1.4077	2.8254
Reg	0.4804	1.0577	1.1334	1.4215	1.7115
Mid	1.6384	2.0684	2.4010	2.8605	3.0398
Top	2.9516	3.0837	3.3596	3.8088	4.3129

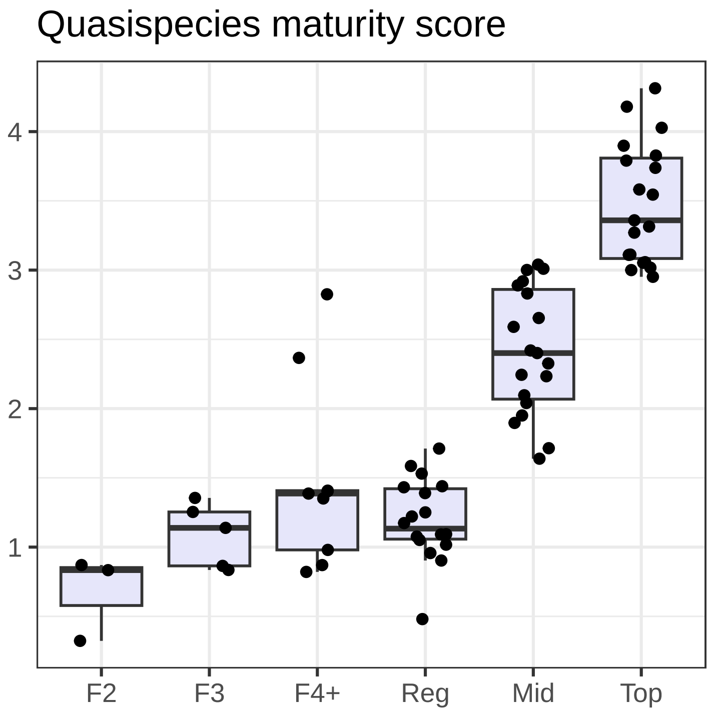

Figure 14: Distribution of quasispecies maturity score per group.

Summary maturity scores in the full cohort span from a minimum value of 0.3231 to a maximum value of 4.313.

Synonymity

The maturation process implies the incorporation of several haplotypes expressing a reduced set of functional phenotypes, which represents a high level of haplotype synonymity in the quasispecies. This characteristic may be measured by the ratio of the number of haplotypes to the number of phenotypes, Rn, the ratio of the frequencies of the master phenotype to the master haplotype, RMstr, and by extension by the ratio of frequencies of the top N phenotypes to the top N haplotypes, RTopN, with $N = \{1, 5, 10, 25\}$.

Table 12: Distribution of synonymity ratios per group

Group	Syn	Min	Q1	Q2	Q3	Max
F2	Rn	1.6489	1.6797	1.7526	2.0819	2.2409
F3	Rn	1.5527	1.7264	1.8341	2.0463	2.5505
F4+	Rn	1.6122	1.7125	2.0051	2.4897	3.5946
Reg	Rn	1.4458	1.7820	2.0058	2.3283	3.4819
Mid	Rn	1.2375	2.4046	2.8093	3.3434	5.4853
Top	Rn	2.2163	2.9994	3.2887	3.6391	5.1412
F2	RMstr	1.1549	1.2140	1.3565	1.5087	1.7221
F3	RMstr	1.2099	1.3229	1.4396	1.6561	2.9936
F4+	RMstr	1.2224	1.4200	1.6337	2.8651	10.1232
Reg	RMstr	1.1646	1.4269	1.6752	2.1419	4.7553
Mid	RMstr	1.0035	2.9348	4.2109	6.2448	102.4531
Top	RMstr	3.8859	7.4734	12.1037	23.8285	169.7600
F2	RTop5	1.1525	1.2050	1.2686	1.3927	1.5242
F3	RTop5	1.2139	1.3111	1.3946	1.5014	3.4939
F4+	RTop5	1.2041	1.4004	1.5344	2.1310	4.4362
Reg	RTop5	1.1640	1.3117	1.4552	1.7290	2.6041
Mid	RTop5	1.0159	1.9782	2.9059	4.7398	30.7962
Top	RTop5	3.3129	4.4457	8.3007	18.4365	111.5929
F2	RTop10	1.1471	1.2026	1.2429	1.3636	1.4276
F3	RTop10	1.2119	1.3055	1.3914	1.4540	4.0678
F4+	RTop10	1.1992	1.3731	1.5066	1.9596	4.0421
Reg	RTop10	1.1635	1.2929	1.4295	1.6118	2.4713
Mid	RTop10	1.0123	1.8452	2.5719	4.0892	19.2964
Top	RTop10	2.4844	4.0156	6.5133	15.9031	101.6615
F2	RTop25	1.1424	1.1961	1.2274	1.3024	1.4004
F3	RTop25	1.2076	1.2913	1.3821	1.4278	4.0665
F4+	RTop25	1.1980	1.3325	1.4219	1.7884	3.4111
Reg	RTop25	1.1611	1.2834	1.4126	1.5778	2.3130
Mid	RTop25	1.0123	1.6815	2.2411	3.3938	13.8369
Top	RTop25	1.8803	3.3788	5.3239	11.1295	80.4898

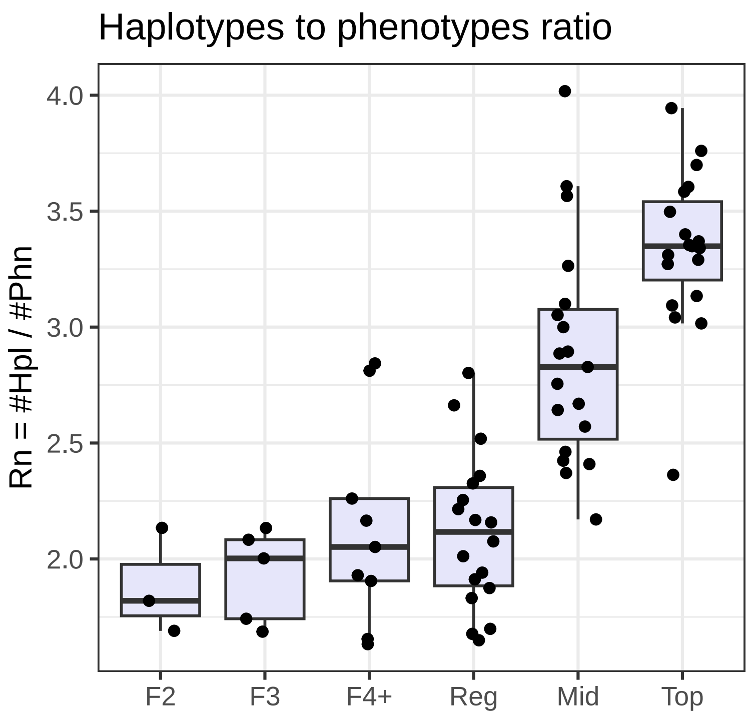

Figure 15: Haplotypes to phenotypes ratio as a measure of quasispecies synonymity.

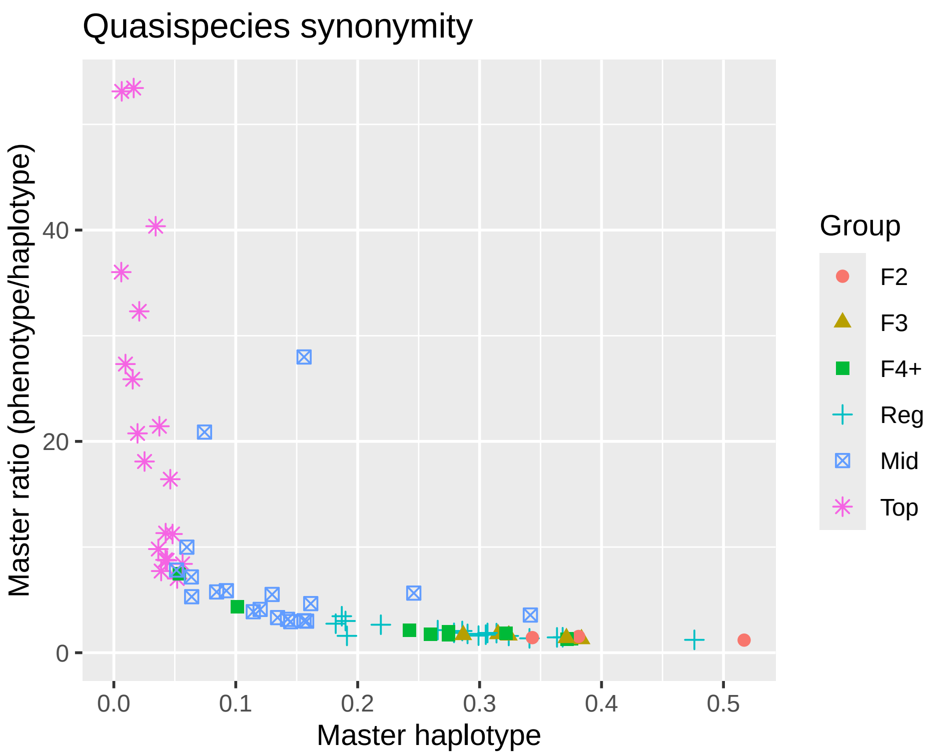

Figure 16: Master phenotype to master haplotype frequency ratio.

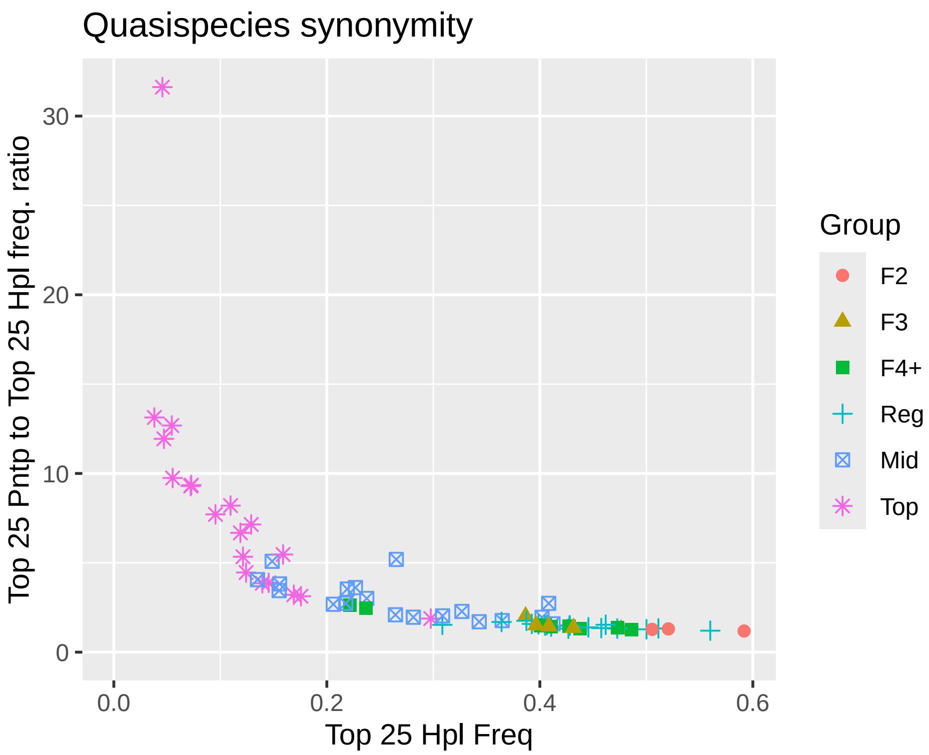

Figure 17: Ratio of the summed frequencies of the top 25 phenotypes to the top 25 haplotypes, as a mesure of synonymity.

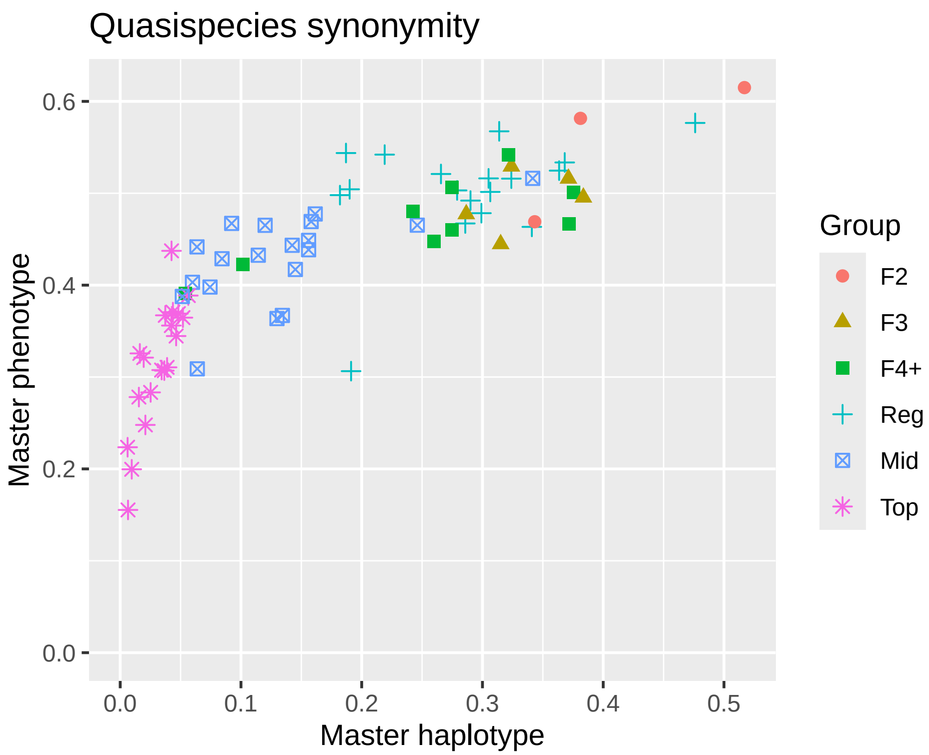

Figure 18: Master haplotype frequency versus master phenotype frequency.

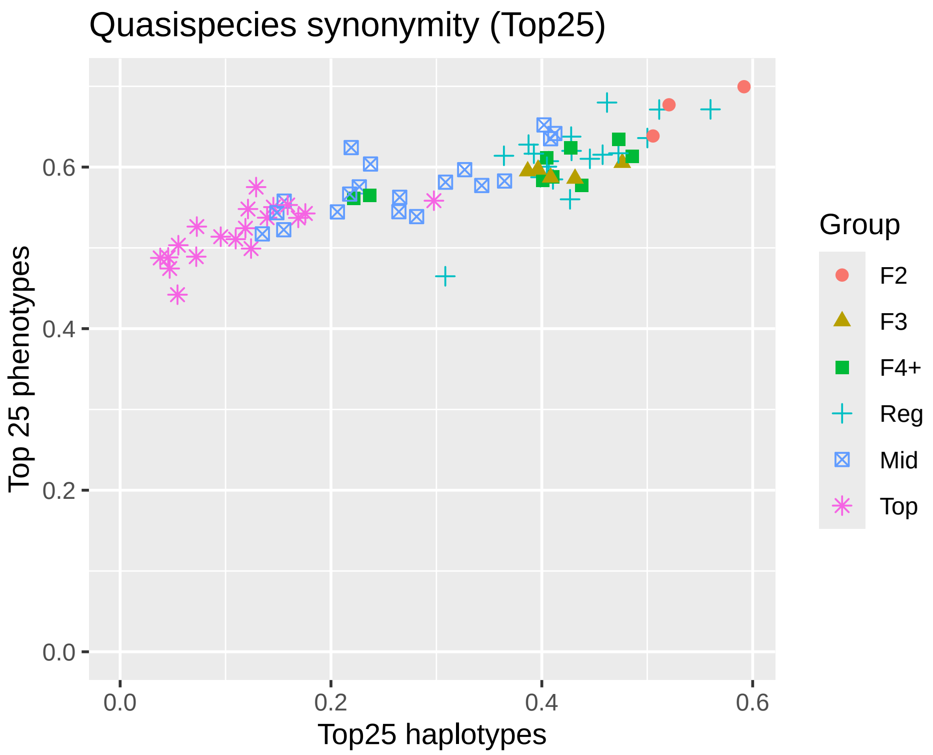

Figure 19: Aggregated frequency of the top 25 haplotypes versus the top 25 phenotypes.

Genetic space explored

If the center of a quasispecies genome cloud is the master haplotype, which is the currently most frequent haplotype in the quasispecies, the amount of genetic space explored by the quasispecies can be evaluated by the mean number of differences (substitutions) per read with respect to the master haplotype in each amplicon. This is observed in a pairwise alignment of all haplotypes relative to the master.

This approach provides a measure of genetic distance from the center, illustrating how far the quasispecies has expanded across the genetic space. It's important to note that this does not imply all substitutions were produced from the current master haplotype; rather, it's an evaluation of the genetic diversity generated around the current master haplotype.

Table 13: Mean substitutions per read, by group

Group	Min	Q1	Q2	Q3	Max
F2	0.5614	0.7191	0.8340	1.0231	1.6815
F3	0.7996	0.8887	1.0704	1.2071	6.4781
F4+	0.7729	1.1356	1.4619	2.2917	4.1540
Reg	0.6153	1.0939	1.3189	1.6792	3.5642
Mid	0.1099	2.0163	2.4697	3.4449	17.0047
Top	2.5506	3.4504	5.3278	7.4580	14.3199

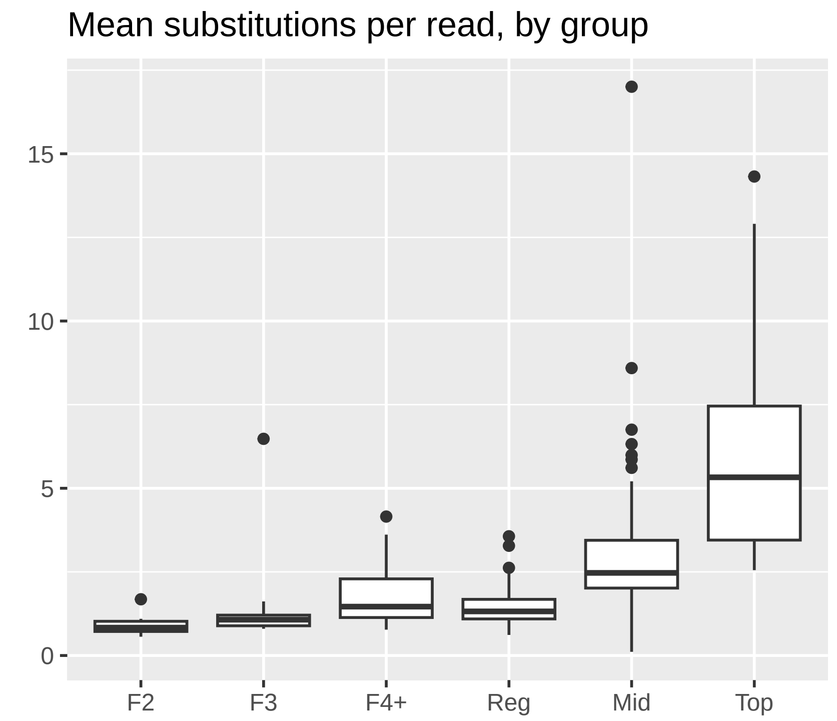

Figure 20: Mean number of substitutions per read relative to the master haplotype, as a measure of the explored genetic space.

A more comprehensive evaluation of the explored genetic space can be provided by analyzing the set of read fractions corresponding to increasing differences relative to the current master haplotype. This approach offers a nuanced view of the quasispecies' genetic diversity, allowing for a more detailed understanding of its population structure. This method provides a more granular assessment of the genetic landscape, complementing the mean genetic distance approach.

Table 14: Read fractions with increasing differences relative to the current master haplotype.

Group	m00	m01	m02	m03	m04	m05	m06	Ov6
F2	0.4356	0.3609	0.1504	0.0420	0.0089	0.0017	0.0002	0.0003
F3	0.3496	0.3600	0.1894	0.0689	0.0188	0.0045	0.0009	0.0080
F4+	0.2345	0.3309	0.2037	0.1076	0.0418	0.0126	0.0031	0.0658
Reg	0.3005	0.3450	0.2009	0.0952	0.0326	0.0088	0.0023	0.0147
Mid	0.1065	0.2004	0.2293	0.1707	0.1024	0.0598	0.0269	0.1041
Top	0.0240	0.0762	0.1150	0.1294	0.1287	0.1013	0.0774	0.3479

In the table above, m00 is the fraction of reads corresponding to the current master, m01 the fraction of reads corresponding to all haplotypes showing a single difference with respect to the master, and so on. With Ov6 the fraction of reads corresponding to haplotypes with more than 6 differences.

Note that, additionally, different subpopulations may coexist within the quasispecies, evolving around different subdominant haplotypes, and contributing to its overall genetic diversity.

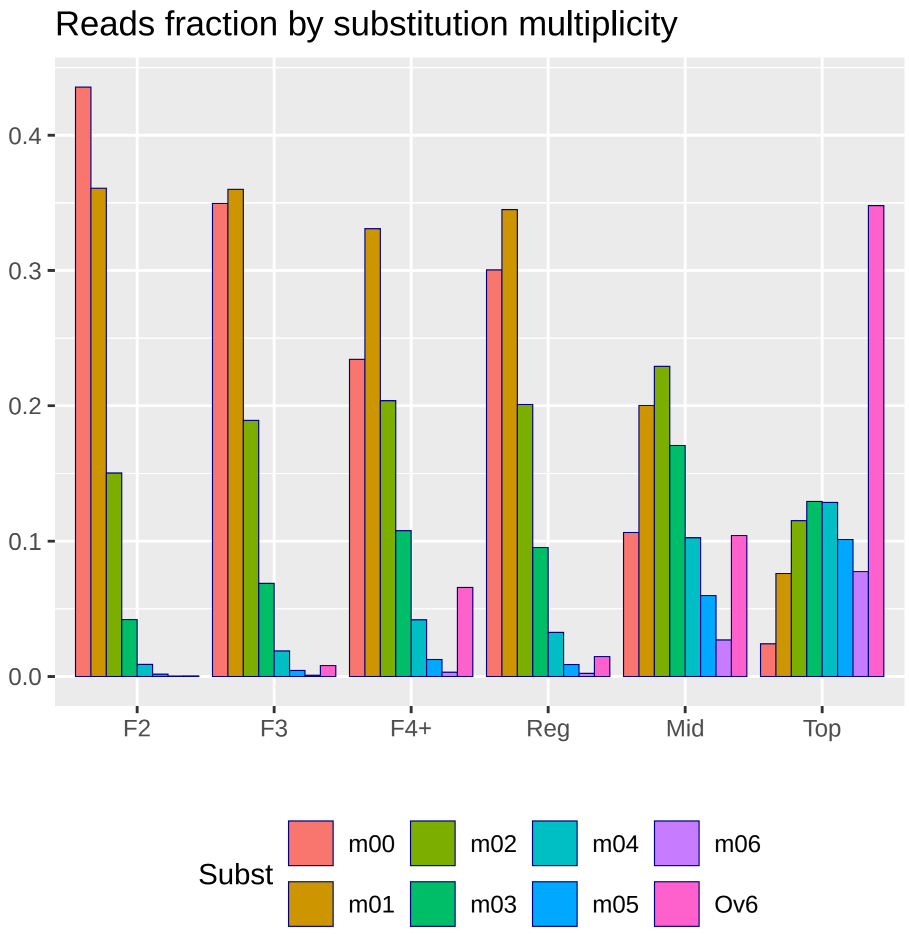

Figure 21: Read fractions with increasing differences relative to the current master haplotype.

Discussion

The association between fibrosis score and quasispecies maturity indices as assessed in the main text, is understood as a direct consequence of infection time. As liver damage progresses, so does quasispecies diversity. Nevertheless this is a simplification, because we know that several factors contribute to quasispecies diversification beyond infection time:

- Long infection duration: Chronic HCV infection persisting over many years allows more time for viral diversification and accumulation of variants.
- Large population size and rapid replication: HCV maintains large population sizes within infected individuals and has short generation times, leading to rapid mutation accumulation. Viral load is commonly used a a surrogate marker for the overall viral population size.
- Immune pressure: The host immune response exerts selective pressure on the virus, driving the evolution of escape mutants that can evade immune recognition.
- Liver microenvironment: The complex liver environment may provide different niches for viral replication, supporting the coexistence of multiple viral subpopulations.
- Bottleneck events: Periodic reductions in viral population size (bottlenecks) followed by expansion can shift the dominant quasispecies over time.
- Antiviral therapy: Treatment with antiviral drugs can exert selective pressure, potentially leading to the emergence of drug-resistant variants.

Despite the simplification, we expect that patients with more advanced liver damage, or longer infection times, will show more diversified quasispecies, and as longer the infection time the more akin to a flat-like quasispecies structure. The main text provided statistical support to this hypothesis.

The cumulative effect of these factors over time contributes to the variability observed in patients with long-lasting chronic HCV infections, often with multiple treatment failures in their clinical history. More advanced infections likely show higher variability in outcomes, as observed in the PCA.

Principal Component Analysis (PCA) on the matrix of quasispecies maturity indicators highlights biological and clinical differences between patient groups. The analysis shows that less advanced infections cluster more tightly, whereas the most advanced infections appear highly scattered. This scatter is primarily attributed to the cumulative effect of varying viral loads throughout disease progression and responses to previous failed treatments.

Patients labeled “Mid” and Top” show higher diversity than F4+ patients, reflecting the complex interaction of these factors, manifested mainly in late stages of chronicity as a cumulative effect.

Several Reg patients with more regular quasispecies have maturity indicator values among F2 to F4+ levels. The 38 patients labelled Mid and Top, with flatter quasispecies show values mainly above F4+ level.

A predictive model based on quasispecies maturity indicators could be developed using data from several hundred patients with known fibrosis scores and clinical information, though the potential benefits of such a model remain unexplored. Our primary objective was to investigate whether quasispecies in chronic HCV infections evolve with fluctuating changes, spiraling around a limited diversity with different master sequences, or if they progress towards flat-like structures with unlimited diversity, characterized by numerous genomes at very low frequencies —a phenomenon observed in two HEV patients with repeatedly failed ribavirin treatments (Colomer-Castell et al. 2023; Gregori, Colomer-Castell, et al. 2024). In this study, the comparison of quasispecies maturity indicators between patients with known liver damage and the rest of the cohort supports our hypothesis that as chronic infections advance, flat-like quasispecies characteristics become increasingly prevalent in the overall quasispecies structure.

A corollary to this discussion is that HCV patients with advanced chronic infections, will correspond

to flatter HCV quasispecies, associated with higher mean quasispecies fitness, and likely exhibit higher resistance to further treatments.

References

Colomer-Castell, S., J. Gregori, D. Garcia-Cehic, M. Riveiro-Barciela, M. Buti, A. Rando-Segura, J. Vico-Romero, et al. 2023. "In-Host HEV Quasispecies Evolution Shows the Limits of Mutagenic Antiviral Treatments." *Int J Mol Sci* 24 (24): 17185. https://doi.org/10.3390/ijms242417185.

Gregori, J., S. Colomer-Castell, M. Ibañez-Lligoña, D. Garcia-Cehic, C. Campos, M. Buti, M. Riveiro-Barciela, et al. 2024. "In-Host Flat-Like Quasispecies: Characterization Methods and Clinical Implications." *Microorganisms* 12 (5): 1011. https://doi.org/10.3390/microorganisms12051011.

Gregori, J., M. Ibañez-Lligoña, S. Colomer-Castell, D. Garcia-Cehic, C. Campos, and J. Quer. 2024. "Association of Liver Damage and Quasispecies Maturity in Chronic HCV Patients: The Fate of a Quasispecies." *Microorganisms* submitted (to appear).
